# Multi-omics reveals the DR5-FXR-CHPT1 axis governs hepatocyte pyroptosis via glycerophospholipid remodeling after major partial hepatectomy in male mice

**DOI:** 10.1038/s41467-026-78113-8

**Published:** 2026-10-07

**Authors:** Yongsheng Tang, Xu Lu, Xi Luo, Qinghai Lian, Yingqiu Song, Manqiu Kang, Zihan Qiao, Zhihua Rao, Linsen Ye, Zenan Yuan, Changchang Jia, Hua Li

**Affiliations:** 1 Department of Hepatic Surgery, Liver Transplantation Center, the Third Affiliated Hospital of Sun Yat-sen University, Guangzhou, China; 2 Department of Cell-Gene Therapy Translational Medicine Research Center, the Third Affiliated Hospital of Sun Yat-sen University, Guangzhou, China; 3 Department of Hepatobiliary and Pancreatic Surgery, Zhuhai Branch, Guangdong Provincial Hospital of Traditional Chinese Medicine, Zhuhai, China; 4 Department of Experimental Medicine, the Third Affiliated Hospital of Sun Yat-sen University, Guangzhou, China

**Keywords:** Mechanisms of disease, Lipid signalling, Protein translocation

## Abstract

Severe liver injury following major partial hepatectomy (PHx) carries high mortality, yet the mechanisms driving irreversible cell death remain poorly understood. Here we show that aberrant lipid metabolism drives inflammatory cell death in the remaining liver tissue. Integrating data from male mouse models and human tissues, we found that death receptor 5 (DR5) acts as a critical regulator. Mechanistically, DR5 impedes the nuclear entry of the Farnesoid X Receptor (FXR), repressing the production of phosphatidylcholine (PC), an essential structural lipid. This lipid deficiency compromises the integrity of cellular membranes, causing severe stress and lethal pyroptosis. Restoring this pathway via DR5 suppression, FXR activation, or PC supplementation significantly alleviated post-surgical liver injury. Overall, we uncover a non-canonical metabolic function of DR5 that disrupts cellular homeostasis. These findings suggest that modulating this metabolic pathway may offer potential avenues for investigating future management strategies for small-for-size syndrome (SFSS) and post-hepatectomy liver failure (PHLF).

## Abstract (web summary)

Here the authors report that after major partial hepatectomy in male mice, Death receptor 5 (DR5) disrupts lipid metabolism via the FXR-CHPT1 axis, triggering cellular stress and pyroptosis. Targeting this pathway may offer a therapeutic strategy to prevent liver failure.

## Introduction

Partial hepatectomy (PHx), split-liver transplantation, viral hepatitis, alcoholic hepatitis, and drug-induced liver injury all can induce acute liver injury^1,2^. This susceptibility is particularly pronounced in marginal donor organs characterized by aging, steatosis, or prolonged cold ischemia time. Clinically, R0 resection remains the most effective strategy for treating benign liver tumors and select malignant hepatic lesions. However, insufficient residual liver function following massive PHx often progresses to small-for-size syndrome (SFSS) and post-hepatectomy liver failure (PHLF). The pathological mechanisms underlying “insufficient regeneration” versus “irreversible damage” differ significantly: the former typically manifests as arrested cell cycle initiation or sluggish kinetics-specifically, the inability of hepatocytes to sufficiently regenerate and restore or maintain a critical functional liver mass^3,4^; conversely, the latter is often accompanied by severe programmed cell death, leading to a continuous and precipitous decline in functional hepatocytes within the remnant liver, ultimately resulting in hepatic failure. Although substantial research has accumulated on strategies to promote liver regeneration, their translational outcomes have fallen short of expectations. This suggests that reliance on pro-regenerative approaches alone may be insufficient to overcome the bottleneck of acute liver injury induced by major PHx^5^. Accordingly, research focus has shifted toward “injury-reducing” strategies that identify and intercept the primary drivers of severe acute liver injury. Synergistically integrating these approaches aims to enhance remnant liver viability and ultimately reduce clinical mortality.

Crucially, PHx is not merely a localized surgical event but a trigger for extensive systemic metabolic reprogramming. Immediate post-operative demand for energy and membrane components initiates rapid adipose tissue mobilization, flooding the remnant liver with free fatty acids. This results in Transient Regeneration-Associated Steatosis (TRAS)^6,7^, a phenomenon traditionally viewed as a vital, temporary energy reservoir fueling the high metabolic costs of hepatocyte proliferation. However, when this lipid influx exceeds the liver’s processing capacity or persists abnormally, it may transition from a physiological fuel source to a pathological driver of injury^8,9^. Pyroptosis is a form of pro-inflammatory programmed cell death mediated by the Gasdermin protein family^10,11^, which has emerged as a key executioner in this transition. Upon stimulation, NOD-like receptor protein 3 (NLRP3) recruits apoptosis-associated speck-like protein containing a CARD (ASC) to assemble the inflammasome, mediating Caspase-1 activation and the subsequent release of IL-1β^12,13^. Previous reports have demonstrated that inhibiting NLRP3 reduces hepatocyte pyroptosis in acetaminophen (APAP)-induced liver injury models^14^. Furthermore, NLRP3 knockout or pharmacological blockade has been shown to significantly promote liver recovery following PHx^15^. Endoplasmic reticulum stress (ERS), acting as a central hub of cellular stress injury, plays a pivotal role in triggering pyroptosis^16^. The sudden surge in lipid metabolic load during TRAS, compounded by severe ischemia-reperfusion injury^17^, and continuous hypoxic hemodynamic shear stress serve as potent disruptors of endoplasmic reticulum (ER) homeostasis^18,19^. Consequently, sustained and intense ERS may transition the liver from a regenerative state into one of irreversible injury.

Glycerophospholipid metabolism serves as the foundation for maintaining ER membrane structural homeostasis. When the proportions of key components such as PC or phosphatidylethanolamine (PE) become dysregulated, ER membrane fluidity is altered^20^, directly activating stress sensors like PERK and IRE1α to induce ERS^21^. Although the dynamic changes in PC following PHx are relatively well-documented^22^, and a decreased hepatic PC/PE ratio has been identified as a predictive marker for survival following PHx, existing studies are largely confined to phenomenological observations. Therefore, the upstream drivers of these glycerophospholipid metabolic alterations, their underlying molecular mechanisms, and the specific impact of glycerophospholipid metabolites on injury in the remnant liver require further investigation.

Multi-omics integration provides an analytical approach for dissecting the metabolic remodeling of complex diseases^23^. Joint analysis of transcriptomics, metabolomics, and lipidomics data suggests that bile acid metabolism mediated by FXR (Nuclear Receptor Subfamily 1 Group H Member 4, NR1H4) plays a critical role in post-hepatectomy injury. FXR exerts significant regulatory control over glycerophospholipid metabolism, and while its agonists demonstrate protective effects in liver diseases, they are primarily utilized for primary biliary cholangitis (PBC). Expanding their application requires a deeper understanding of the molecular mechanisms governing their function. Furthermore, death receptor 5 (DR5, also known as TNFRSF10B, TRAIL-R2, or CD262) is a transmembrane receptor whose activation can occur intracellularly, independent of its extracellular ligands or TNF-related apoptosis-inducing ligands (TRAIL)^24^. Activation of DR5 regulates downstream effectors-such as FADD, caspase-8, Bid, and caspase-3-playing a vital role in cell injury signal transduction. While DR5 has frequently been targeted in therapies for solid tumors^25,26^ (including melanoma, breast, lung, and colon cancers), its role in benign liver disease models remains less defined^27^.

Here, we show that DR5 acts as a key feature gene regulating glycerophospholipid metabolism after partial hepatectomy. Utilizing a massive hepatectomy animal model combined with multi-omics data and a machine learning model optimized through 15 algorithms and 207 parameters, we identify core genes regulating this process. We observe that the upregulation of DR5 in the remnant liver hinders the nuclear translocation of FXR, thereby impeding the transcription of Choline Phosphotransferase 1 (*Chpt1*), a key enzyme in glycerophospholipid metabolism. This results in a PC imbalance, which exacerbates ERS and ultimately leads to hepatocyte pyroptosis. Furthermore, we demonstrate that interfering with DR5 expression, activating FXR, and providing supplemental PC all effectively attenuate pyroptosis, thereby ameliorating acute liver injury following 70% PHx. In summary, this study elucidates the molecular mechanisms of irreversible damage after PHx from the perspective of metabolic remodeling and provides therapeutic targets for clinical interventions against unpredictable SFSS or PHLF.

## Results

### Severe pyroptosis accompanied by extensive lipid metabolism dysregulation in the acute injury phase following extensive PHx

**Fig. 1:**
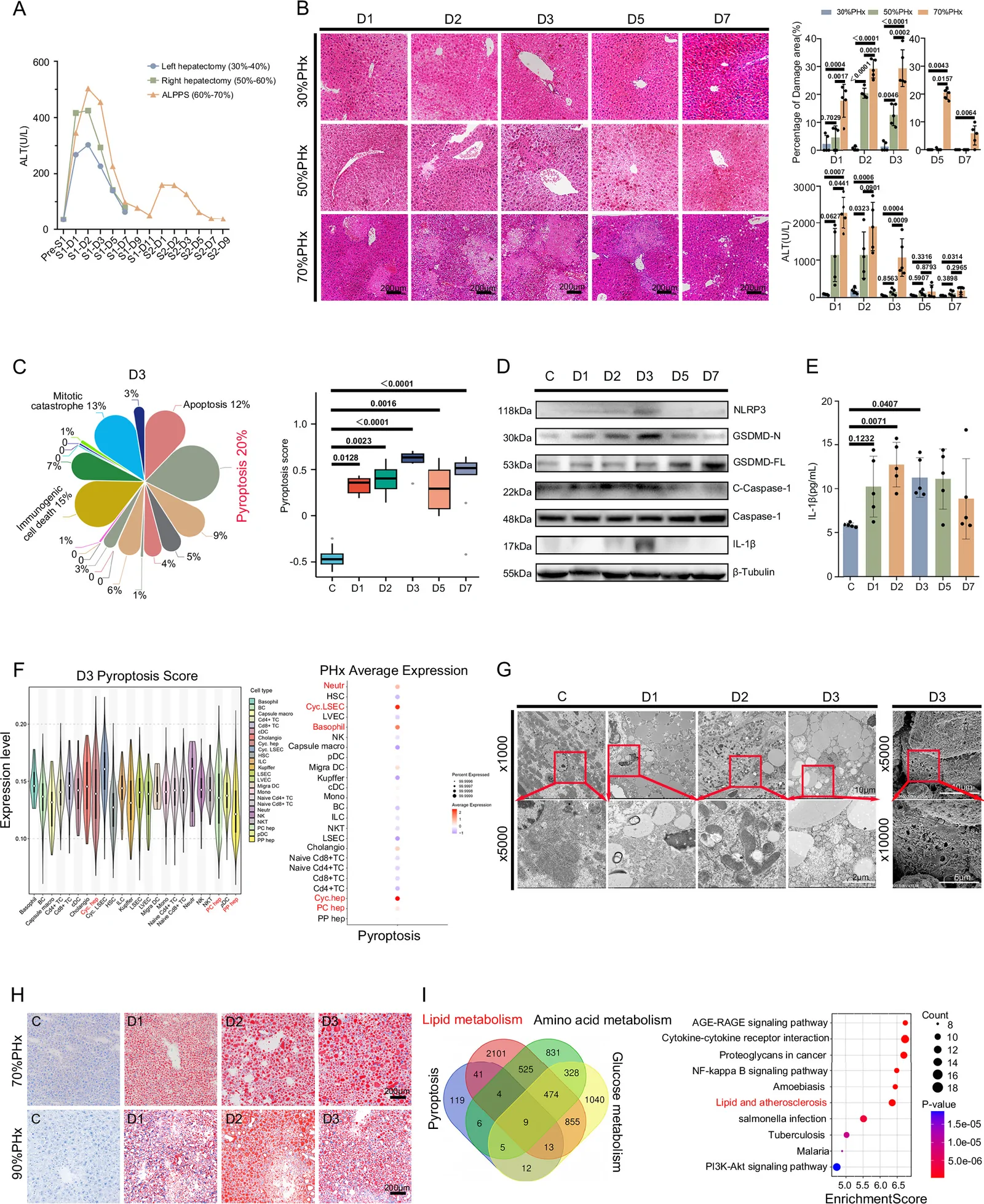
Severe pyroptosis accompanied by extensive lipid metabolism dysregulation in the acute injury phase following 70% PHx. **A** Serum ALT levels following anatomical left hemihepatectomy (*n* = 174 patients), right hemihepatectomy (*n* = 145 patients) and ALPPS (*n* = 52 patients). **B** Representative H&E staining (Scale bar=200 μm, *n* = 5 independent experiments), liver damage area quantification, and serum ALT in mice with 30%, 50%, and 70% resection volumes (*n* = 5 mice per group). Damage area: D1-D3 (mean ± SD; one-way ANOVA/Tukey’s test), D5-D7 (median with IQR; Kruskal-Wallis/Dunnett’s test). ALT: mean ± SD; D1 analyzed via Welch’s ANOVA/two-sided Games-Howell test, others via one-way ANOVA/Tukey’s test. **C** Cell death pattern classification from D3 following 70% PHx RNA-seq DEGs (left panel). GSVA scores for DEGs and PRGs (right panel; box plots: center line=median, limits=25th/75th percentiles, whiskers=1.5 x IQR, outliers plotted individually; *n* = 6 mice per group for D1-D3, *n* = 8 for D5, *n* = 9 for D7; one-way ANOVA/Dunnett’s test). **D** Representative western blot of pyroptosis-related proteins post-70% PHx (*n* = 5 independent experiments). **E** Serum IL-1β ELISA post-70% PHx (*n* = 5 mice per group. mean ± SD; one-way ANOVA/Dunnett’s test). **F** ScRNA-seq pyroptosis scores at D3 (violin plots: median, IQR, and 1.5 x IQR shown as white dots, thick bars, and thin lines) and average expression across hepatic clusters post-70% PHx using UCell (*n* = 5 mice per group). **G**TEM and SEM images post-70% PHx (Scale bar=10 μm, *n* = 3 independent experiments). **H** Oil Red O staining post-70% and 90% PHx (Scale bar=200 μm, *n* = 5 independent experiments). **I** Venn diagram of upregulated PRGs across lipid, glucose, and amino acid pathways (left panel), and KEGG enrichment of upregulated PRGs (right panel) from 70% PHx RNA-seq. Abbreviations: ALT Alanine Aminotransferase; ALPPS, associating liver partition and portal vein ligation for staged hepatectomy; PHx, partial hepatectomy; H&E, Hematoxylin and Eosin; IQR, interquartile range; DEGs, differentially expressed genes expressed genes; PRGs, pyroptosis-related genes; GSVA, gene set variation analysis; ELISA, Enzyme-Linked Immunosorbent Assay; ScRNA-seq, single-cell RNA sequencing; SEM, scanning electron microscopy; TEM, transmission electron microscopy; KEGG, Kyoto Encyclopedia of Genes and Genomes. All *p* values are shown in the figure, Source data are provided as a Source Data file.

To investigate the injury dynamics of the remnant liver tissue following PHx, we retrospectively collected data from 371 cases, including anatomical left hemihepatectomy (resection volume 30–40%, *n *= 174 patients), anatomical right hemihepatectomy (resection volume 50–60%, *n* = 145 patients), and associating liver partition and portal vein ligation for staged hepatectomy (ALPPS) (resection volume 60–70%, *n* = 52 patients). We systematically analyzed postoperative liver enzyme changes after these resection volumes (Fig. 1A, Supplementary Fig. 1A). The results showed that Alanine Aminotransferase (ALT) and Aspartate Aminotransferase (AST) levels peaked within 48–72 h (postoperative day 3, D3) following left hemihepatectomy, right hemihepatectomy, and ALPPS Step-1, indicating an acute injury phase within the first 3 days. Notably, although the future liver remnant (FLR) regenerates sufficiently after ALPPS Step-1, ALT and AST levels also remained elevated within the first 3 days following Step-2. Other critical indicators of liver function, activated partial thromboplastin time (APTT), albumin (ALB), and total bilirubin (TB), showed significant alterations during this period (Supplementary Fig. 1B), confirming an acute injury phase. The probability of developing SFSS or PHLF increased with larger resection volumes (Supplementary Fig. 1C). If the histopathological features and molecular pathological alterations during acute liver injury can be elucidated, and the key molecules and pathways critically influencing this injury identified, then targeted interventions could be applied to alleviate damage to the remnant liver following PHx, thereby substantially reducing the incidence of unpredictable death.

Subsequently, we established 30%, 50% and 70% PHx models in C57BL/6 mice (Fig. 1B, Supplementary Fig. 1D). Histopathological and serum liver function analyses revealed that the 30% PHx group exhibited minimal injury and minor enzyme fluctuations. The 50% PHx group showed significantly increased injury areas and functional impairment within 3 days compared to the 30% PHx group. The 70% PHx group demonstrated the most severe injury and functional impairment, which progressively worsened over the first 3 days, indicating an acute injury phase. We hypothesized that the progressive exacerbation of remnant liver injury is likely dominated by a specific form of cell death. Therefore, analysis of transcriptomic data across 21 cell death modes revealed that pyroptosis-related genes (PRGs) were among the most significantly upregulated differentially expressed genes (DEGs) (Fig. 1C, Supplementary Fig. 1E). Subsequent GSVA scoring based on these PRGs further confirmed that pyroptosis activity was markedly elevated following 70% PHx at the acute injury phase, reaching its peak at D3 compared to the control and earlier postoperative time points (D1, D2) (Fig. 1C). Western Blot (WB) analysis confirmed significant upregulation of pyroptosis-associated proteins at the acute injury phase (Fig. 1D, Supplementary Fig. 1F). ELISA results confirmed that IL-1β and IL-18 were significantly upregulated at the acute phase (Fig. 1E, Supplementary Fig. 1G). To further identify the primary effector cells undergoing pyroptosis following extensive PHx, we conducted an in-depth analysis of Single-cell RNA sequencing (scRNA-seq) data. Evaluation using the UCell algorithm revealed a significant and highly specific elevation in pyroptosis pathway scores within hepatocyte subpopulations across all evaluated postoperative time points (D1-D3) (Fig. 1F, Supplementary Fig. 1H). Multiplex immunofluorescence (mIF) staining corroborated these findings, demonstrating that the core pyroptotic executioner GSDMD-N highly colocalized with the hepatocyte marker HNF4α, but to a lesser extent with endothelial (CD31) or myeloid (CD11b) cell markers (Supplementary Fig. 1I). Collectively, these results strongly indicate that hepatocytes as the primary effector cells undergoing pyroptosis following extensive PHx. Scanning Electron Microscopy (SEM) revealed extensive membrane pore formation on hepatocytes in the remnant liver at D3; Transmission Electron Microscopy (TEM) indicated severe disruption of mitochondrial and endoplasmic reticulum (ER) structures compared to controls both organelles exhibited marked swelling and membrane rupture (Fig. 1G). Specifically, D3 samples showed severe destruction of mitochondrial structures and ER disintegration.

Having thoroughly characterized the pyroptotic process in the classical 70% PHx model, we next sought to eliminate the possibility of model-specific bias and determine whether this mechanism is universally applicable to more severe, lethal PHLF. To this end, we performed a parallel, systematic validation of our core findings using a 90% PHx model. Strikingly consistent with the trends observed in the 70% PHx model, WB analysis revealed that the expression of core pyroptosis pathway proteins was significantly upregulated in a time-dependent manner, peaking at D3 (Supplementary Fig. 1J, K). Concurrently, ELISA confirmed a drastic and progressive systemic release of the downstream proinflammatory cytokines IL-1β and IL-18 (Supplementary Fig. 1L). Furthermore, TEM and SEM unambiguously captured the hallmark ultrastructural features of pyroptosis (Supplementary Fig. 1M). Collectively, these parallel validation results conclusively demonstrate that the NLRP3/Caspase-1/GSDMD-mediated pyroptosis is not an idiosyncrasy of the 70% resection model, but rather a universal and critical pathological driver of acute liver injury following extensive hepatectomy. Furthermore, scRNA-seq on the D3 revealed that, compared with 70% PHx, hepatocyte injury was substantially increased in the remnant liver after 90% PHx, with prominent upregulation of PRGs in hepatocytes. Consistently, the GSVA pyroptosis score in hepatocytes was markedly elevated relative to that in the 70% PHx group (Supplementary Fig. 1N). These findings suggest that excessive activation of pyroptosis likely drives more severe liver injury.

Prompted by the observation of pronounced vacuolization in H&E staining sections and increased lipid droplets under TEM, we performed Oil Red O staining, which confirmed a significant accumulation of intrahepatic lipids following PHx (Fig. 1H, Supplementary Fig. 1O). Furthermore, RNA-seq analysis of the 70% PHx model revealed that significantly upregulated PRGs were also involved in regulating lipid metabolism pathways (Fig. 1I). Consistent findings were observed among the upregulated PRGs in hepatocytes identified by scRNA-seq in the 90% PHx compared to the 70% PHx (Supplementary Fig. 1P). These results strongly suggest that severe pyroptosis occurs, accompanied by significant dysregulation of lipid metabolism in the acute injury phase following extensive PHx.

### Disruptions in lipid metabolism may contribute to the induction of pyroptosis

**Fig. 2:**
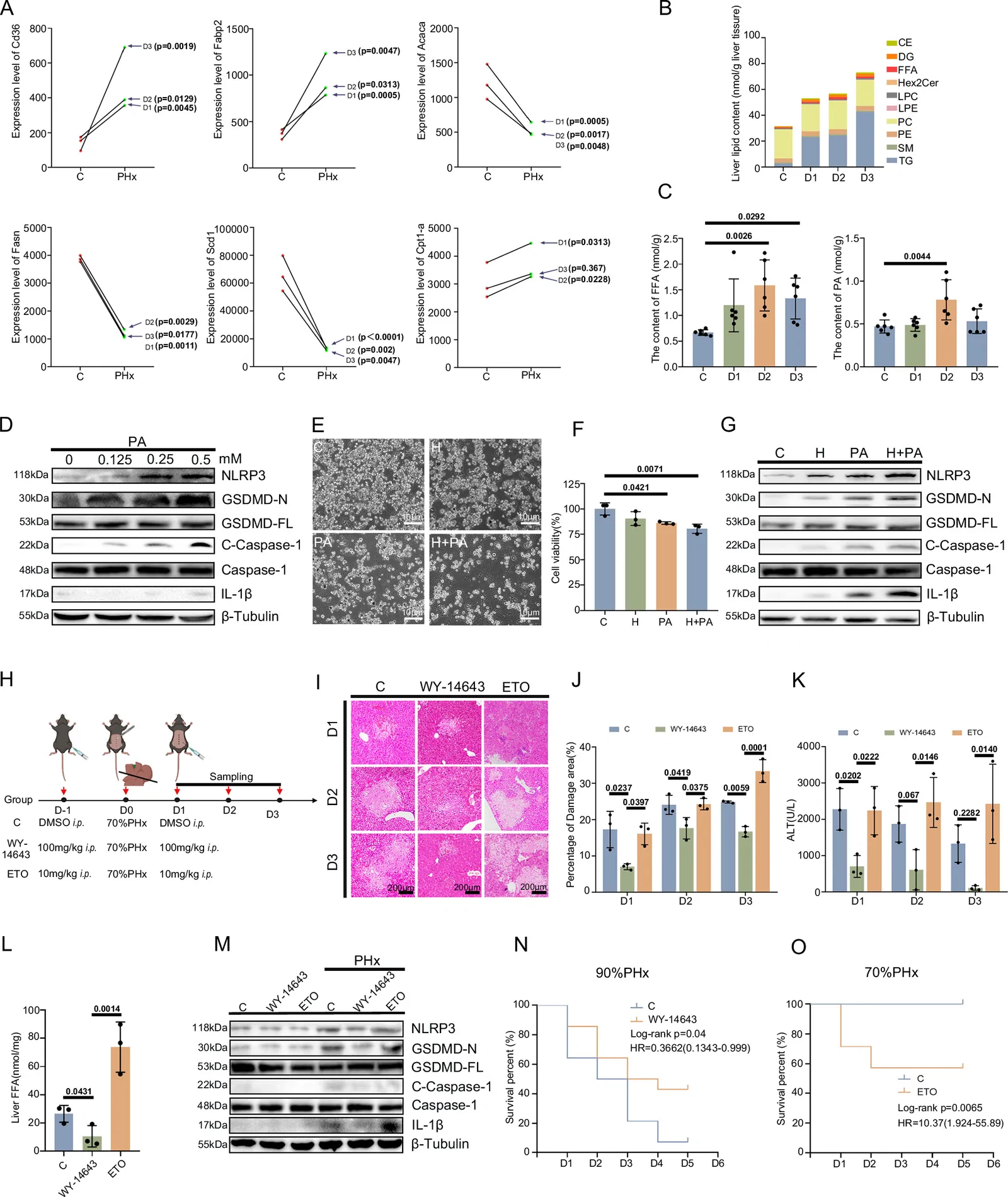
Disordered lipid metabolism triggers pyroptosis following 70% PHx. **A** Expression trends of lipid metabolism-related genes post-70% PHx (*n* = 6 mice per group. mean ± SD; two-tailed paired Student’s *t* test; Fabp2 at D2 via two-sided Wilcoxon matched-pairs signed rank test). **B** Lipidomic profiling of lipid contents post-70% PHx (*n* = 6 mice per group). **C** FFAs and PA contents (*n* = 6 mice per group. mean ± SD; one-way ANOVA/Dunnett’s test). **D** Representative western blot analysis of pyroptosis-related proteins in cells treated with different concentrations of PA (*n* = 3 independent experiments. Representative images are shown from one of three independent experiments with similar results). **E** Representative images of cell morphology and density under combined H + PA treatment (Scale bar = 10 μm). **F** Cell viability assessed by CCK-8 assay (*n* = 3 independent experiments. mean ± SD; one-way ANOVA/Tukey’s test). **G** Representative western blot of pyroptosis-related proteins in the cell model (*n* = 3 independent experiments). **H** Schematic diagram of experimental design (Created in BioRender. Jia, C. (2026) https://BioRender.com/ixquskv) (*n* = 3 mice per group). **I** Representative H&E images of liver tissue post-70% PHx (Scale bar=200 μm, *n* = 3 independent experiments). **J** Quantification of liver damage area post-70% PHx (*n* = 3 mice per group. mean ± SD; one-way ANOVA/Tukey’s test). **K** Serum ALT levels post-70% PHx (*n* = 3 mice per group. mean ± SD; one-way ANOVA/Dunnett’s test). **L** FFAs content at D3 post-70% PHx (*n* = 3 mice per group. mean ± SD; one-way ANOVA/Tukey’s test). **M** Representative western blot of pyroptosis-related proteins at D3 post-70% PHx (*n* = 3 independent experiments). **N** Survival curve post-90% PHx with WY-14643 (*n* = 14 mice per group. Kaplan-Meier method/Log-rank test). **O** Survival curve post-70% PHx with ETO (*n* = 14 mice per group. Kaplan-Meier method/Log-rank test). Abbreviations: C Control, PHx partial hepatectomy, FFAs free fatty acids, H Hypoxia, PA palmitic acid, CCK-8 Cell Counting Kit-8, H&E Hematoxylin and Eosin, ALT Alanine Aminotransferase, ETO Etomoxir. All *p* values are shown in the figure, Source data are provided as a Source Data file.

As the body’s primary metabolic organ, the liver plays a pivotal role in maintaining systemic homeostasis, and its dysfunction can exert extensive and far-reaching effects on whole-body physiological stability. Therefore, we further explored the acute-phase pathophysiological alterations and identify potential triggers of pyroptosis. The expression of lipid import genes (*Cd36, Fabp2*) and lipid droplet formation genes (*Plin2*) significantly increased, while lipid synthesis genes (*Acaca, Fasn, Scd1*) significantly decreased. Conversely, genes related to lipid consumption (*Cpt1-α, Acadm, Acads, Crat, Hadhb*) were elevated (Fig. 2A, Supplementary Fig. 2A–G). Integrating our experimental findings with prior literature, we conclude that energy requirements are primarily fulfilled through β-oxidation of imported lipids, without a corresponding increase in de novo lipid synthesis following 70% PHx. This imbalance leads to substantial lipid accumulation in remnant hepatocytes, and such dysregulation of lipid metabolism is highly likely to underlie the observed injury. LC-MS quantitative lipidomics at various time points showed a significant elevation in free fatty acids (FFAs) within 3 days post-PHx, with Palmitic Acid (PA) occupying the largest proportion (Fig. 2B, C). To simulate these conditions in vitro, THLE-2 hepatocytes were cultured with increasing concentrations of PA. Higher PA concentrations significantly inhibited cell proliferation (Supplementary Fig. 2H–J) and induced progressive pyroptosis (Fig. 2D, Supplementary Fig. 2K). Considering the ischemia-reperfusion injury and continuous hypoxic portal flow from the gut during PHx, we added hypoxia treatment to the 0.25 mmol/L PA condition to better mimic the hepatic microenvironment. Light microscopy revealed exacerbated cell injury in the Hypoxia+PA group (Fig. 2E), and CCK-8 assays showed significantly reduced viability (Fig. 2F). WB results confirmed more severe pyroptosis in the Hypoxia+PA group (Fig. 2G, Supplementary Fig. 2L). To validate whether lipid burden causes pyroptosis, we treated 70% PHx models with WY-14643 (to reduce intracellular FFAs) and Etomoxir (ETO, to inhibit β-oxidation and increase intracellular FFAs) (Fig. 2H). Remarkably, WY-14643 treatment significantly reduced the injury area and improved liver function, whereas ETO treatment significantly increased the injury area (Fig. 2I, J) and worsened liver function (Fig. 2K, Supplementary Fig. 2M). Consistent with their mechanisms, WY-14643 significantly lowered intrahepatic FFAs levels, while ETO elevated them (Fig. 2L). WB analysis showed that WY-14643 downregulated, while ETO upregulated, pyroptosis-related proteins (Fig. 2M, Supplementary Fig. 2N). To evaluate the hepatoprotective effects of WY-14643 under extreme stress, we employed a lethal 90% PHx model, demonstrating that the treatment effectively rescued the survival limit (Fig. 2N). While ETO significantly reduced survival in the 70% PHx model (Fig. 2O). These findings suggest that lipid metabolism disorder is a likely cause of pyroptosis post-hepatectomy.

### PC supplementation effectively mitigates ERS, thereby reducing hepatocyte pyroptosis following 70% PHx

**Fig. 3:**
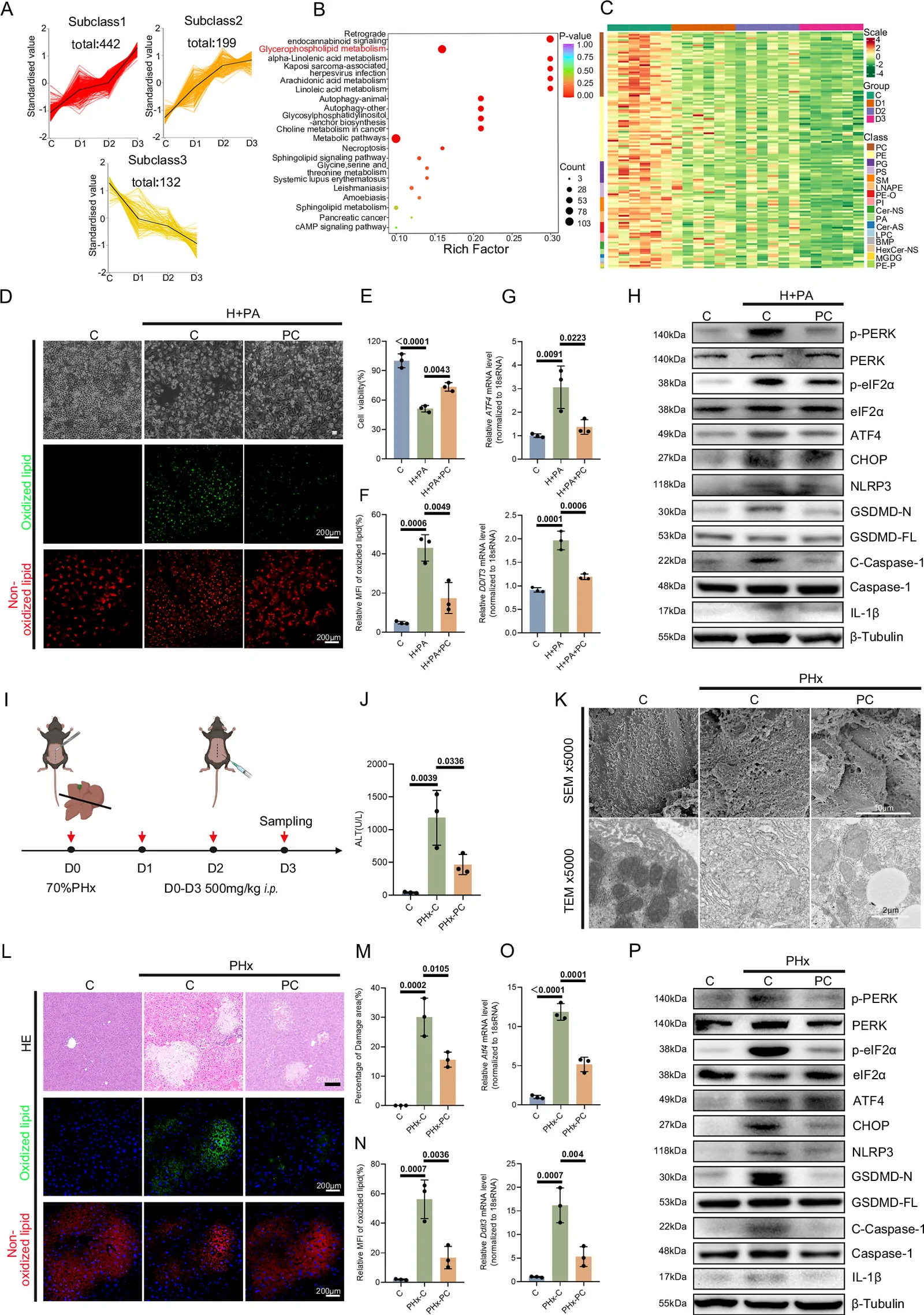
PC supplementation alleviates ERS and reduces post-70% PHx pyroptosis. K-Means clustering of temporal expression profiles for differential lipids (**A**), KEGG pathway enrichment of Subclass 3 (**B**), and heatmap of Subclass 3 (**C**) following 70% PHx (*n* = 6 mice per group). In vitro model (*n* = 3 independent experiments): Representative morphological and C11 BODIPY lipid staining images (**D**, Scale bar=200 μm), CCK-8 cell viability (**E**), relative MFI of C11 BODIPY staining (**F**), relative mRNA expression of ERS-related genes (**G**), and western blot analysis of ERS- and pyroptosis-related proteins (**H**). **I** Experimental design schematic (Created in BioRender. Jia, C. (2026) https://BioRender.com/ixquskv) (*n* = 3 mice per group). In vivo assessments at D3 post-70% PHx (*n* = 3 mice per group or *n* = 3 independent experiments): Serum ALT levels (**J**), SEM (Scale bar=10 μm) and TEM (Scale bar=2 μm) images (**K**), H&E and C11 BODIPY lipid staining images (**L**, Scale bar=200 μm), quantification of liver damage area percentage (**M**), relative MFI of C11 BODIPY staining (**N**), relative mRNA expression of ERS-related genes (**O**), and western blot analysis of ERS- and pyroptosis-related proteins (**P**). Abbreviations: C Control, KEGG Kyoto Encyclopedia of Genes and Genomes, H Hypoxia, PA Palmitic acid, PHx partial hepatectomy, PC Phosphatidylcholine, ALT Alanine Aminotransferase, MFI Mean fluorescence intensity, ERS Endoplasmic reticulum stress, SEM scanning electron microscopy, TEM transmission electron microscopy. All quantitative data are presented as Mean ± SD. Statistical significance was determined using one-way ANOVA followed by Tukey’s multiple comparisons test. All *p* values are shown in the figure, and Source data are provided as a Source Data file.

During the early phase of hepatectomy (within 48 h), surgical trauma prompts the release of damage-associated molecular patterns (DAMPs), such as HMGB1, to activate the NF-κB signaling pathway, ultimately triggering NLRP3-mediated pyroptosis^28–30^ (Supplementary Fig. 3A–C). However, the mechanisms underlying the subsequent exacerbation of pyroptosis warrant deeper exploration. A crucial clue emerged from our preceding observation that lipid remodeling extensively influences the occurrence of pyroptosis following 70% PHx. To pinpoint the specific lipid types affecting pyroptosis, we analyzed lipidomics data. Of 1474 detected lipids, 733 showed significant daily changes within the first 3 days post-70% PHx, with glycerolipids emerging as particularly prominent (Supplementary Fig. 3D). The K-Means clustering algorithm was applied to the abundance matrix of significantly differential lipids, for investigating the dynamic temporal patterns of lipid profiles during the acute injury phase. Using Euclidean distance measurements, metabolites sharing highly congruent dynamic trajectories were automatically partitioned into 3 subclasses (Fig. 3A): Subclass1 was dominated by rising PE (Supplementary Fig. 3E), Subclass2 showed similar PC and PE changes (Supplementary Fig. 3F); Subclass3 showed the most significant downregulation of PC, correlating closely with glycerophospholipid metabolism (Fig. 3B, C). As a key component of cellular membranes, PC deficiency likely compromises membrane fluidity and stability, triggering ERS and cellular injury. In vitro supplementation with exogenous PC effectively protected cells against hypoxia and lipid accumulation-induced injury, reduced oxidative lipids, increased viability (Fig. 3D, E), alleviated ERS (Fig. 3F, G), and consequently reduced pyroptosis (Fig. 3H, Supplementary Fig. 3G). Consistent with these in vitro findings, in vivo PC supplementation ameliorated functional deterioration in the remnant liver after 70% PHx (Fig. 3I, J, Supplementary Fig. 3H). SEM revealed reduced membrane pore formation, while TEM demonstrated mitigated mitochondrial swelling with preserved membrane and cristae architecture; ER dilation was markedly relieved, yielding clearer overall ultrastructure compared to controls (Fig. 3K). H&E staining showed a significant decrease in the proportion of injured tissue and reduced intracellular oxidative lipids following PC supplementation (Fig. 3L, M). RT-qPCR and WB further confirmed that PC supplementation alleviated ER stress and consequently diminished pyroptosis (Fig. 3O, P, Supplementary Fig. 3I). Collectively, PC depletion exerts an extensive influence on pyroptosis, and its supplementation effectively mitigates ER stress, thereby reducing hepatocyte pyroptosis after PHx.

### DR5 is a key gene driving pyroptosis after 70% PHx

**Fig. 4:**
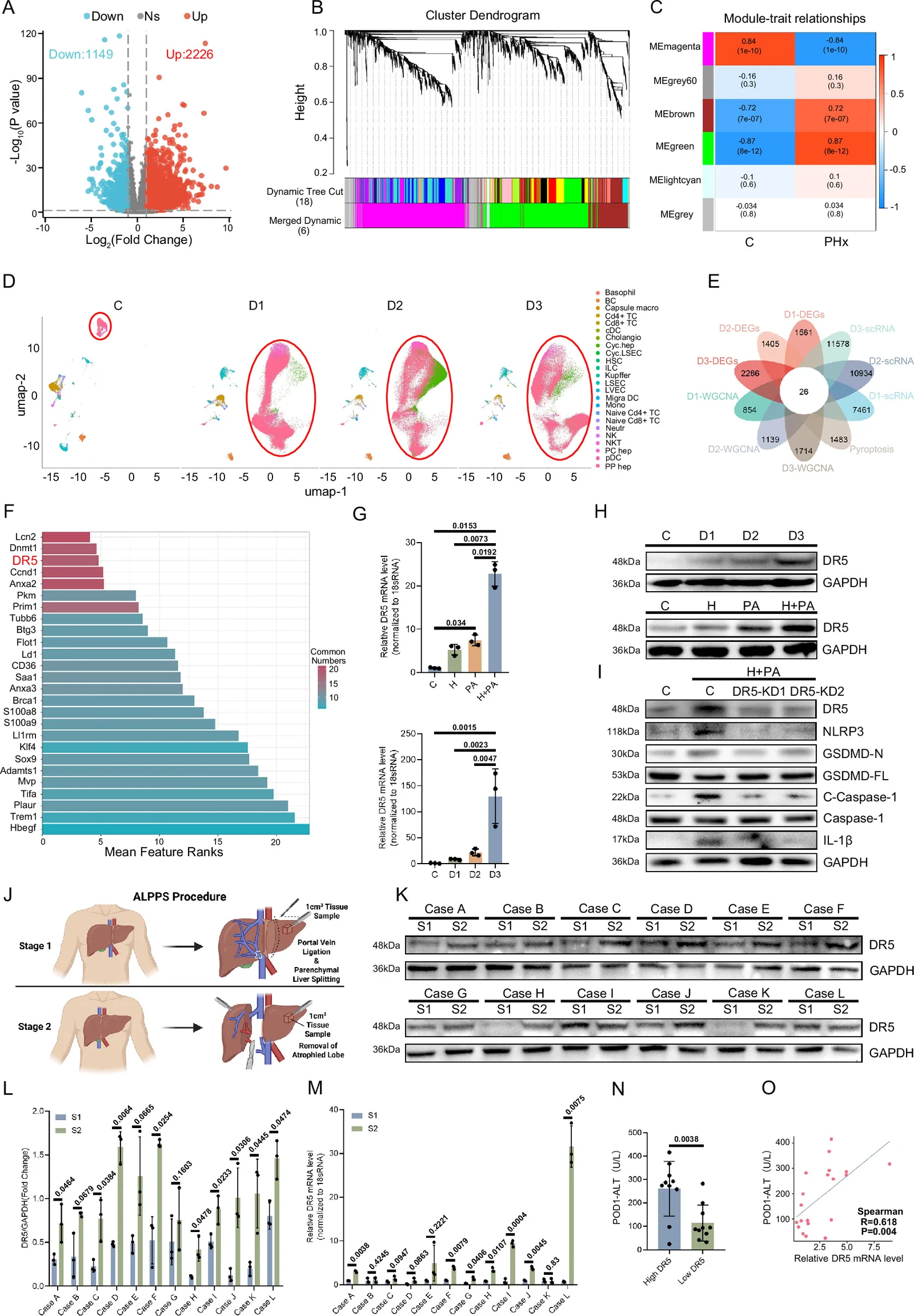
DR5 is a potential key gene driving pyroptosis following 70% PHx. RNA-seq and WGCNA in the first 3 days (*n* = 36 samples from 18 mice): Volcano plot of DEGs (**A**), cluster dendrogram (**B**), and module-trait relationships heatmap (**C**). **D** ScRNA-seq hepatocyte clustering (*n* = 5 mice per group). Venn diagram intersecting RNA-seq DEGs (*n* = 36 samples from 18 mice), scRNA-seq DEGs (CNP0002310), WGCNA modules (*n* = 36 samples from 18 mice), and pyroptosis-related genes (**E**), and machine learning-based key gene importance ranking (**F**). DR5 relative mRNA expression (**G**, mean ± SD; one-way ANOVA/Tukey’s test) and western blot (**H**) in cell models (top panel) and post-70% PHx liver tissue (bottom panel) (*n* = 3 independent experiments). Western blot of pyroptosis proteins following DR5 knockdown (DR5-KD) in cell models (**I**, *n* = 3 independent experiments). Clinical ALPPS analysis: Schematic diagram of the ALPPS procedure and liver tissue sampling at two stages (**J**) (Created in BioRender. ld, l8. (2026) https://BioRender.com/wy9dm5g). DR5 western blot (**K**), quantification (**L**), and mRNA expression (**M**) in ALPPS stage 1 (S1) and 2 (S2) liver tissues from 12 cases (*n* = 3 technical replicates, mean ± SD; two-tailed paired Student’s *t* test). Postoperative serum ALT in low vs. high DR5 ALPPS-S2 groups (**N**, *n* = 10 patients per group. mean ± SD; two-tailed unpaired Student’s *t* test) and Spearman correlation between S2 DR5 mRNA and peak postoperative ALT (**O**, *n* = 20 patients; two-sided Spearman’s rank correlation test). Abbreviations: WGCNA Weighted Gene Co-expression Network Analysis, ScRNA-seq single-cell RNA sequencing, DEGs differentially expressed genes expressed genes, ALT alanine aminotransferase. All *p* values are shown in the figure. Source data are provided as a Source Data file.

To identify the key gene driving pyroptosis and providing a therapeutic target, we analyzed transcriptomic data, identifying 3375 DEGs (2226 upregulated, 1149 downregulated) in the first 3 days (Fig. 4A). Weighted Gene Co-expression Network Analysis (WGCNA) grouped all genes into 18 modules, which were merged into 6 distinct modules based on similarity (Fig. 4B). The Brown and Green modules showed high Pearson correlation coefficients (>0.7) with PHx (Fig. 4C), subsequent biological functional analysis revealed that these modules are highly associated with postoperative injury and repair responses (Supplementary Fig. 4A). Consequently, they were prioritized for the screening of key genes. Using scRNA-seq data from a public database (CNP0002310)^31^, we identified DEGs in hepatocytes across time points (Fig. 4D) and intersected them with transcriptomic DEGs and PRGs, yielding 26 candidate genes (Fig. 4E). Machine learning algorithms were employed to determine the most critical gene (Supplementary Fig. 4B, C). The top 10 genes by importance were *Lcn2, Dnmt1, DR5, Ccnd1, Anxa2, Pkm, Prim1, Tubb6, Btg3, and Flot1* (Fig. 4F). Death Receptor 5 (DR5) consistently ranked high across multiple models, highlighting its potential causal link to liver injury. A similar approach identified 20 lipid metabolism-related genes (Supplementary Fig. 5A–C), where DR5 also ranked highly (Supplementary Fig. 5D). Validation via RT-qPCR and WB showed elevated DR5 expression in both animal and cell models (Fig. 4G, H, Supplementary Fig. 5E). We established two distinct DR5-knockdown (DR5-KD) THLE-2 cell lines (Supplementary Fig. 5F) and observed that DR5-KD significantly reduced pyroptosis-related protein expression in vitro (Fig. 4I, Supplementary Fig. 5G). In human ALPPS samples, DR5 expression was significantly higher in liver tissue from the second stage surgery compared to the first (Fig. 4J–M). Retrospective analysis revealed that patients with high DR5 expression had significantly elevated ALT and AST levels following Step 2 (Fig. 4N, Supplementary Fig. 5H), and DR5 expression positively correlated with serum ALT and AST on postoperative day 1 (POD1) (Fig. 4O, Supplementary Fig. 5I).

### DR5 promotes pyroptosis by impeding the nuclear translocation of FXR

**Fig. 5:**
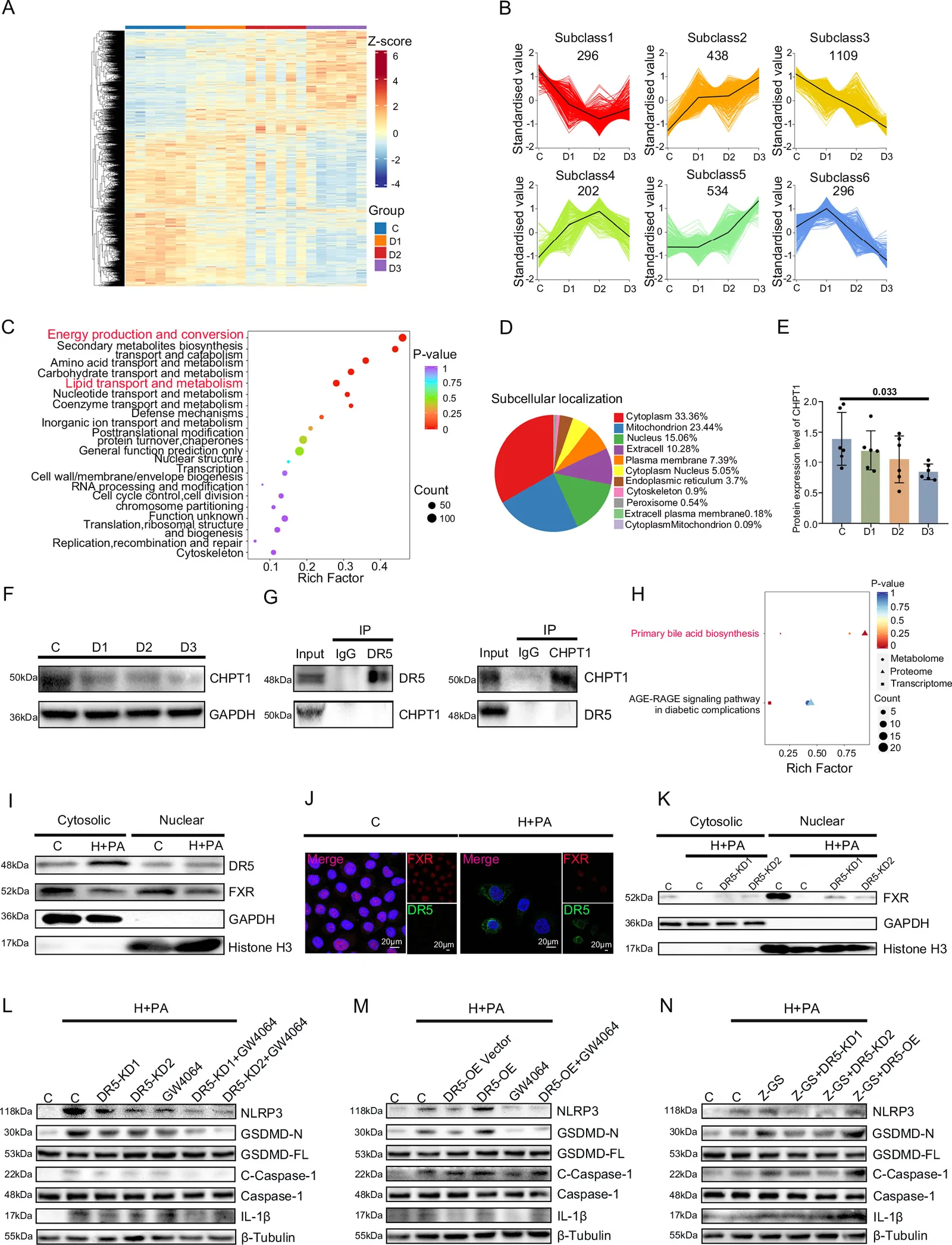
DR5 promotes pyroptosis by impeding FXR nuclear translocation following 70% PHx. Proteomic analysis following 70% PHx: Heatmap (**A**) and K-Means clustering of temporal expression profiles within the differential liver proteome (**B**) (*n* = 6 mice per group). KOG functional (**C**) and subcellular localization (**D**) analyses of Subclass 3. CHPT1 differential expression from proteomics (**E**, *n* = 6 mice per group. mean ± SD; one-way ANOVA/Dunnett’s test) and western blot validation post-70% PHx (**F**, *n* = 3 independent experiments). **G** Co-IP assay detecting DR5 and CHPT1 interaction (*n* = 3 independent experiments). **H** Joint multi-omics (transcriptomics, lipidomics, proteomics) functional enrichment analysis. Representative western blot of cytoplasmic and nuclear DR5 and FXR (**I**) and immunofluorescence of DR5 and FXR (**J**, Scale bar=20 μm) in cell models (*n* = 3 independent experiments). Representative western blot analyses in cell models (*n* = 3 independent experiments): Cytoplasmic and nuclear FXR expression post-DR5 knockdown (DR5-KD) (**K**); Pyroptosis-related proteins following DR5-KD combined with FXR agonist GW4064 (**L**), DR5 overexpression (DR5-OE) combined with GW4064 (**M**), and FXR inhibitor Z-GS combined with DR5-KD or DR5-OE (**N**). Abbreviations: C Control, H Hypoxia, PA Palmitic acid, KOG euKaryotic Orthologous Groups, Co-IP Co-immunoprecipitation, DR5 Death receptor 5, FXR Farnesoid X receptor, CHPT1 Choline phosphotransferase 1. All *p* values are shown in the figure. Source data are provided as a Source Data file.

To elucidate the molecular mechanism by which DR5 drives pyroptosis, we performed proteomics profiling of the remnant liver at multiple time points post-PHx. A total of 6579 proteins were detected, of which 2875 exhibited significant daily fluctuations within the first 3 days (Fig. 5A). Functions were enriched in lipid transport and metabolism, predominantly localizing to the cytoplasm (Supplementary Fig. 5A). Similarly, K-Means clustering algorithm was applied to the temporal expression profiles of the differentially expressed proteins. Based on their temporal trajectories, these proteins were stratified into six Subclasses (Fig. 5B, Supplementary Fig. 6B, C). Subclass3, which mirrored the PC trend, was closely related to lipid metabolism (Fig. 5C) and cytoplasmic localization (Fig. 5D). Notably, CHPT1 (Choline Phosphotransferase 1), a key enzyme for PC synthesis, belonged to Subclass 3 (Fig. 5E). WB confirmed sustained downregulation of CHPT1 post-PHx (Fig. 5F, Supplementary Fig. 6D). CO-IP assays indicated no direct interaction between DR5 and CHPT1 (Fig. 5G). Multi-omics integration (Metabolomics + Proteomics + Transcriptomics) pointed towards primary bile acid synthesis as a central functional node (Fig. 5H, Supplementary Fig. 6E, F). This suggested FXR (a regulator of bile acid synthesis) as a potential link. Nuclear-cytoplasmic fractionation assays revealed that as cytoplasmic DR5 levels rose, nuclear FXR significantly decreased (Fig. 5I, Supplementary Fig. 6G). MIF confirmed that increased DR5 expression in cell models correlated with reduced nuclear FXR (Fig. 5J). Conversely, DR5-KD restored FXR nuclear translocation (Fig. 5K, Supplementary Fig. 6H). Functionally, both DR5-KD and the FXR agonist GW4064 effectively reduced pyroptosis in THLE-2 cells, with synergistic effects when combined (Fig. 5L). In parallel, we established a DR5-overexpressing (DR5-OE) THLE-2 cell line (Supplementary Fig. 6I, J), DR5-OE increased pyroptosis, which was mitigated by GW4064 (Fig. 5M). The FXR antagonist Z-GS exacerbated pyroptosis, an effect attenuated by DR5-KD but aggravated by DR5-OE (Fig. 5N). These findings confirm that DR5 promotes pyroptosis by hindering FXR nuclear translocation.

### The DR5-FXR-CHPT1 axis regulates pyroptosis after 70% PHx

**Fig. 6:**
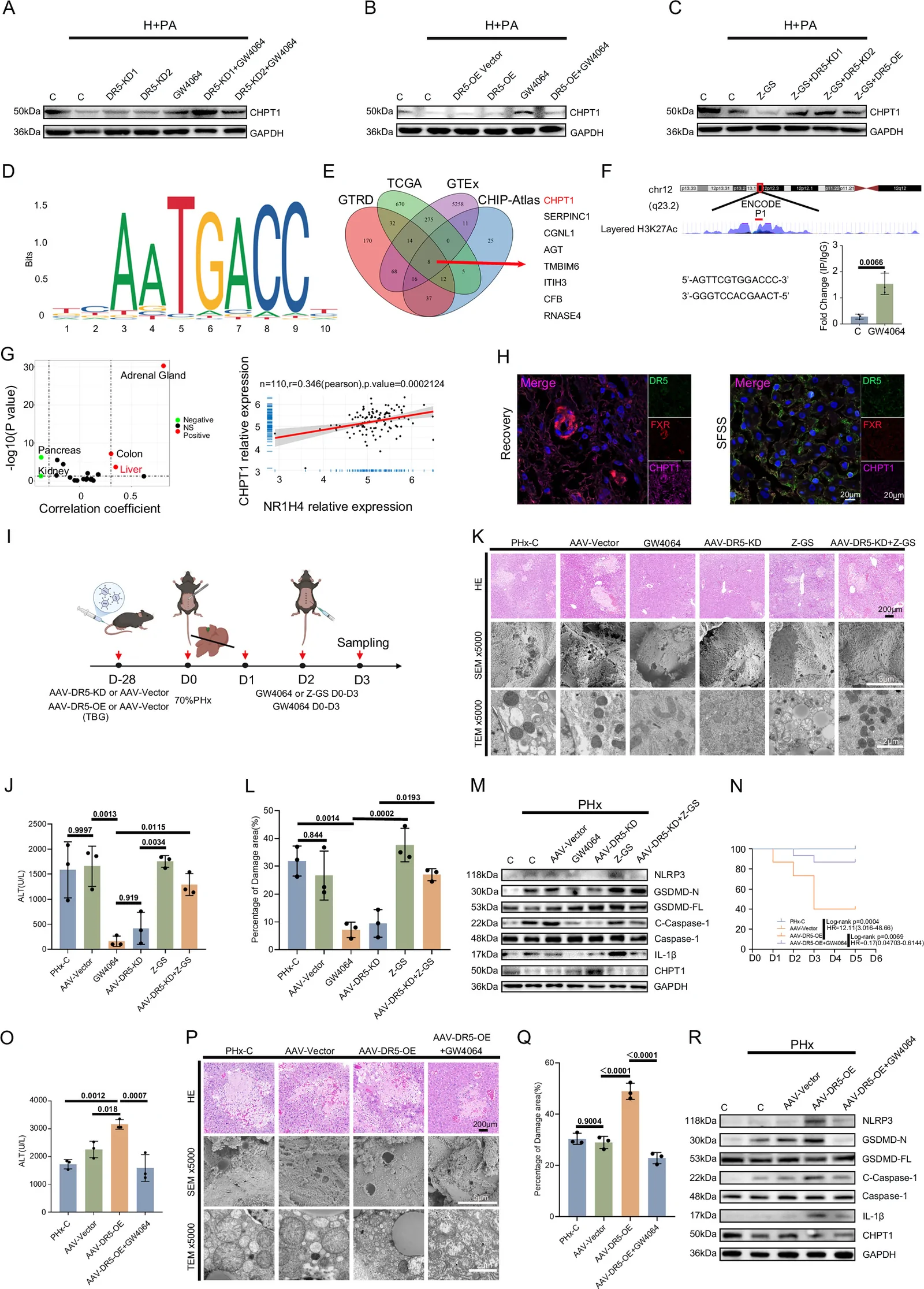
The DR5-FXR-CHPT1 axis regulates pyroptosis following 70% PHx. **A**–**C** Western blot analysis of CHPT1 in cell models treated with FXR agonist GW4064 or inhibitor Z-GS combined with DR5 knockdown (DR5-KD) or overexpression (DR5-OE) (*n* = 3 independent experiments). FXR regulates Chpt1 transcription: FXR DNA binding motif (**D**), Venn diagram of predicted target databases (**E**), Chpt1 promoter binding mapping and ChIP-qPCR validation (**F**, *n* = 3 independent experiments; two-tailed Student’s *t* test), and CHPT1/NR1H4 (FXR) correlation in GTEx liver database (**G**, two-sided Pearson correlation test, shaded band represents 95% confidence interval). **H** Representative immunofluorescence of DR5, FXR, and CHPT1 in clinical ALPPS liver tissues (recovery vs. SFSS) (Scale bar=20 μm, *n* = 3 independent experiments). **I** Schematic of in vivo AAV and drug treatments in 70% PHx mice (Created in BioRender. Jia, C. (2026) https://BioRender.com/ixquskv). Post-70% PHx evaluations with AAV-DR5-KD and Z-GS: Serum ALT (**J**, *n* = 3 mice per group; one-way ANOVA/Tukey’s test), H&E (Scale bar=200 μm), SEM (Scale bar=5 μm), and TEM (Scale bar=2 μm) images (**K**, *n* = 3 independent experiments), liver damage area quantification (**L**, *n* = 3 mice per group; one-way ANOVA/Tukey’s test), and western blot of CHPT1 and pyroptosis proteins (**M**, *n* = 3 independent experiments). Post-70% PHx evaluations with AAV-DR5-OE and GW4064: Survival curve (**N**, *n* = 15 mice per group; Kaplan-Meier method/Log-rank test), serum ALT (**O**, *n* = 3 mice per group; one-way ANOVA/Dunnett’s test), morphological images matching **K** (**P**, *n* = 3 independent experiments), damage area quantification (**Q**, *n* = 3 mice per group; one-way ANOVA/Tukey’s test), and western blot analysis (**R**, *n* = 3 independent experiments). Abbreviations: GTRD Gene Transcription Regulation Database, TCGA The Cancer Genome Atlas; GTEx Genotype-Tissue Expression, H&E Hematoxylin and eosin; SEM Scanning electron microscopy; TEM Transmission electron microscopy. All quantitative data are mean ± SD. All *p* values are shown in the figure. Source data are provided as a Source Data file.

We next investigated how FXR regulates *Chpt1*. In cell models, DR5-KD or GW4064 treatment significantly increased CHPT1 protein levels (Fig. 6A, Supplementary Fig. 7A). Conversely, DR5-OE downregulated CHPT1, an effect rescued by GW4064 (Fig. 6B, Supplementary Fig. 7B). The FXR antagonist Z-GS potentiated the DR5-OE-inducing downregulation of CHPT1 (Fig. 6C, Supplementary Fig. 7C), confirming that DR5 regulates CHPT1 via FXR activity. FXR is a transcription factor, JASPAR analysis visualized its preferred DNA binding motif (Fig. 6D). Integration of GTRD, TCGA, GTEx, and ChIP-Atlas databases indicated *Chpt1* is a target of FXR (Fig. 6E). We identified a clear FXR binding peak in the *Chpt1* promoter region enriched with the H3K27Ac enhancer mark. ChIP-qPCR confirmed that GW4064 treatment significantly upregulated *Chpt1* transcription, verifying direct positive regulation (Fig. 6F). Analysis of GTEx, TCGA, and CCLE databases showed a significant positive correlation between FXR (NR1H4) and CHPT1 expression in normal tissues (Fig. 6G, Supplementary Fig. 7D, E). In human ALPPS samples, SFSS patients exhibited significantly upregulated DR5 and reduced FXR/CHPT1 colocalization and expression compared to those with smooth recovery (Fig. 6H). To evaluate this axis in vivo, mice were administered an AAV-DR5-KD vector featuring a TBG promoter for hepatocyte-specific targeting. We confirmed its precise liver tropism and potent silencing efficiency in the 70% PHx model (Fig. 6I, Supplementary Fig. 7F–J). Treatment with AAV-DR5-KD or GW4064 significantly decreased ALT and AST levels (Fig. 6J, Supplementary Fig. 7K) and reduced liver injury (Fig. 6K–L). However, FXR inhibition with Z-GS exacerbated injury and nullified the protective effects of AAV-DR5-KD and GW4064. SEM and TEM showed that Z-GS treatment induced severe membrane pore formation, sinusoidal congestion, and mitochondrial swelling, whereas AAV-DR5-KD or GW4064 maintained organelle integrity. WB showed that Z-GS downregulated CHPT1 and upregulated pyroptosis markers, while AAV-DR5-KD or GW4064 upregulated CHPT1 and suppressed pyroptosis (Fig. 6M, Supplementary Fig. 7L).

To further substantiate these findings, an AAV-DR5-OE vector with verified liver-specific tropism and high transduction efficiency was employed in the 70% PHx model (Supplementary Fig. 7M–Q). AAV-DR5-OE treatment markedly decreased mouse survival, whereas FXR activation via GW4064 co-treatment mitigated this escalating mortality trend (Fig. 6N). Concurrently, AAV-DR5-OE-treated mice exhibited drastically elevated serum ALT and AST levels (Fig. 6O, Supplementary Fig. 7R) alongside a substantially increased area of hepatic damage (Fig. 6P, Q). Activation of FXR with GW4064 markedly ameliorated these liver injuries, partially abrogating the detrimental impacts of DR5 overexpression. SEM and TEM imaging further confirmed that GW4064 effectively maintained organelle integrity, counteracting the AAV-DR5-OE-induced aggravation of plasma membrane poration, sinusoidal congestion, and mitochondrial swelling. Mechanistically, WB analysis revealed that AAV-DR5-OE suppressed CHPT1 expression and triggered the massive upregulation of pyroptosis markers (NLRP3, GSDMD-N, Cleaved Caspase-1, and IL-1β). Notably, GW4064 intervention reversed these molecular alterations, restoring CHPT1 levels while effectively dampening the pyroptotic protein signature (Fig. 6R, Supplementary Fig. 7S).

**Fig. 7:**
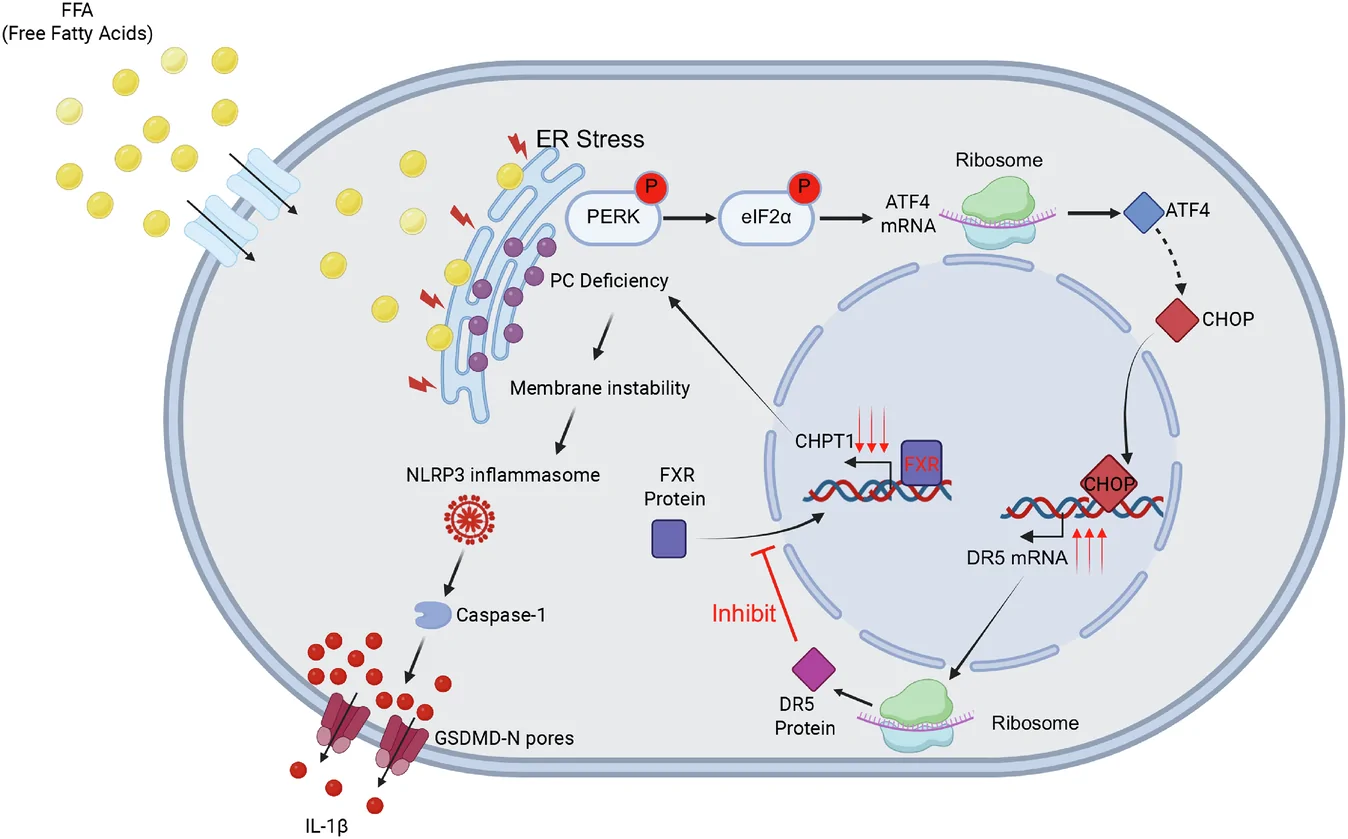
Following 70% PHx, systemic lipid mobilization leads to massive lipid influx into hepatocytes, triggering ERS. This upregulates death receptor 5 (DR5) expression, which impedes farnesoid X receptor (FXR) nuclear translocation, thereby reducing *Chpt1* (Cholinephosphotransferase 1) transcription and diminishing phosphatidylcholine (PC) synthesis. Consequently, ER stress is progressively exacerbated, ultimately culminating in lethal pyroptosis (Created in BioRender. Jia, C. (2026) https://BioRender.com/oelctbq).

In summary, activating FXR increases the transcription of CHPT1, thereby enhancing PC generation, balancing ERS, and inhibiting hepatocyte pyroptosis (Fig. 7). The DR5-FXR-CHPT1 axis is a potent hepatoprotective pathway regulating injury after 70% PHx.

## Discussion

In this study, we employed a large-volume PHx animal model combined with multi-omics analysis and machine learning to elucidate the potential causes and molecular mechanisms underlying the unpredictable and severe injury (PHLF and SFSS) following liver resection. We revealed that pyroptosis driven by endoplasmic reticulum stress (ERS) is a critical factor leading to functional decompensation of the remnant liver. Crucially, we identified and validated the metabolic-inflammatory regulatory axis driven by DR5: the aberrant upregulation of DR5 induced by massive PHx impedes FXR nuclear translocation and inhibits CHPT1 transcription. We reveal the DR5-FXR-CHPT1 axis as the core pathway orchestrating hepatic PC metabolic reprogramming post-hepatectomy. Importantly, we show that severe PC depletion triggers lethal endoplasmic reticulum stress (ERS), subsequently activating GSDMD-mediated pyroptosis. This disrupts glycerophospholipid metabolic homeostasis and compromises ER membrane stability, thereby exacerbating ERS and progressing to lethal hepatocyte pyroptosis. These findings have prompted us to expand the intervention strategy for postoperative PHLF and SFSS from purely “promoting regeneration” to “reducing injury,” further breaking the bottleneck in treating acute liver injury induced by large-volume hepatectomy.

PHx triggers systemic metabolic changes, particularly adipose tissue mobilization^32,33^. While transient regeneration-associated steatosis (TRAS) is recognized as an energy source for regeneration^7,34^, our lipidomics data further reveal a persistent and substantial lipid burden in the liver during the first three days following 70% PHx. This burden is exacerbated by perioperative pathophysiological changes associated with liver resection, including ischemia-reperfusion injury, whereby the remnant liver must rapidly assume full functional load; elevated portal venous pressure^35^; dysregulated blood flow redistribution in the remnant liver^36^, leading to increased perfusion with hypoxic blood, and potential effective circulating volume insufficiency due to reduced hepatic vascular bed^17,37^. These factors collectively predispose the remnant liver to irreversible injury. When we reduced this lipid burden using WY-14643, remnant liver injury was mitigated, and survival in 90% PHx models improved. Conversely, exacerbating the lipid burden with ETO worsened injury and survival. Sustained excessive lipid input brings a massive lipid burden that first triggers ERS by reducing ER membrane fluidity, disrupting calcium homeostasis, and inducing oxidative damage, which directly interferes with protein folding. When the stressed ER attempts to restore balance, it often paradoxically activates lipid synthesis transcription factors, leading to further intracellular lipid accumulation^20^. This pathological feedback loop of “lipotoxicity-induced stress, and stress-exacerbated accumulation” likely culminates in severe damage to remnant hepatocytes^38^.

We found that pyroptosis is the primary type of cell death leading to severe remnant hepatocyte injury. This form of programmed cell death, mediated by caspase-1 and accompanied by the release of IL-1β and IL-18, triggers a robust inflammatory response. Under physiological homeostasis, the expression of NLRP3 inflammasome components in hepatocytes is indeed maintained at a low basal level^39^. However, our findings reveal that extensive PHx robustly upregulates their expression to trigger hepatocyte pyroptosis. We postulate that damage-associated molecular patterns (DAMPs) released by surgical trauma, sudden shifts in hemodynamic shear stress, and the acute, massive influx of lipids post-surgery collectively serve as ‘priming signals’. These signals activate the intracellular NF-κB cascade, thereby transcriptionally inducing Nlrp3 and Pro-IL-1β^28–30^. Nevertheless, the mechanisms driving the subsequent and more critical exacerbated phase of pyroptosis warrant further in-depth exploration. While its participation in tissue injury during liver ischemia-reperfusion and steatosis has been confirmed^40,41^, its role in hepatocyte injury induced by lipid metabolism imbalance after massive PHx remains unclear, particularly its association with glycerophospholipid metabolic disorders. Glycerophospholipids are not only major components of cell and organelle membranes but are also linked to neuroinflammatory levels^42^; maintaining glycerophospholipid metabolic homeostasis in the brain is key to inhibiting neuroinflammation and delaying cognitive decline. Upregulated PC levels help improve lipid deposition in non-alcoholic fatty liver disease (NAFLD) and reduce hepatic steatosis, indicating that the stability of glycerophospholipid metabolism is an important pathway for maintaining metabolic homeostasis^43^. Our lipidomics data show that glycerophospholipid metabolism is very important in 70% PHx. Notably, exogenous PC supplementation in the 70% PHx markedly attenuated remnant hepatocyte pyroptosis. Severe ERS triggered by glycerophospholipid dysregulation likely promotes NLRP3 inflammasome assembly and Caspase-1 activation. This mechanism not only explains the frequent association of SFSS with severe systemic inflammatory response syndrome in clinical practice but also accounts for the limited efficacy of anti-inflammatory therapies alone, as persistent inflammation arises from uncorrected upstream defects in membrane lipid metabolism. Our findings align with previous reports that NLRP3 knockout enhances liver regeneration while providing the missing upstream metabolic trigger.

Through the integration of multi-omics data and machine learning, we precisely identified DR5 as a core gene regulating glycerophospholipid metabolism, filtering out noise inherent in traditional screening. This data-driven approach allowed us to redefine the role of DR5 in non-neoplastic liver injury. Unlike its classical role as a receptor for TRAIL in inducing tumor apoptosis^25,44^, we demonstrate that in the context of post-hepatectomy pathology, DR5 acts as a key upstream “molecular switch” for metabolic reprogramming. Crucially, we found that DR5 activation after PHx occurs intracellularly, independent of extracellular ligands. This non-canonical function expands the biological understanding of the death receptor family and explains why DR5 exerts significant pathological control even in sterile surgical injury lacking exogenous immune attack.

At the mechanistic level, we established a causal link between signal transduction and organellar membrane homeostasis via the “DR5–FXR–CHPT1” axis. Dynamic balance between PC and PE ratios is essential for membrane fluidity and ER function. Our data show that DR5-mediated impairment of FXR nuclear translocation directly suppresses transcription of CHPT1, the rate-limiting enzyme in PC synthesis. Intriguingly, the FXR agonist GW4064 markedly upregulated both CHPT1 transcription and protein expression. Comprehensive validation using DR5-KD/OE combined with FXR agonists and inhibitors confirmed the functional integrity of the “DR5–FXR–CHPT1” axis. Injury arising from metabolic substrate deficiency is more refractory to reversal than conventional protein folding overload, leading to sustained hyperactivation of stress sensors and conversion of the normally adaptive unfolded protein response (UPR) into toxic pro-death signaling^45^.

From a translational perspective, this study offers different metabolism-targeted strategies for preventing and treating PHLF and SFSS, conditions currently lacking specific therapeutic targets. Our findings suggest that targeting the DR5–FXR axis or directly modulating glycerophospholipid metabolism holds therapeutic promise. Exogenous PC supplementation or pharmacological FXR activation to restore CHPT1 expression could stabilize ER membrane architecture in the remnant liver, thereby interrupting the vicious “ERS–pyroptosis” cycle. We validated this pathway in animal models and human ALPPS specimens. However, SFSS involves more complex portal hemodynamics and inter-individual metabolic variability^46^; thus, the clinical relevance of the DR5–FXR axis requires further validation in larger cohorts. Additionally, whether glycerophospholipid regulation of pyroptosis is hepatocyte-specific and involves interactions with the hepatic immune microenvironment remains to be clarified^47^. The optimal therapeutic window for intervening in this pathway also warrants future investigation.

While our integrative analysis successfully prioritized DR5, we must acknowledge the limitations of the machine learning approach utilized in this study. The algorithms were trained on a relatively small cohort of 36 samples with 26 input genes, which inherently restricts the robustness and broad generalizability of the predictive models. Consequently, the machine learning results should be interpreted as an adjunctive screening tool rather than standalone definitive evidence. Nevertheless, our core mechanistic conclusions regarding the DR5-FXR-CHPT1 axis remain highly robust, as they are independently and strongly substantiated by comprehensive multi-omics differential expression, scRNA-seq, and extensive in vivo and in vitro experimental validations.

In summary, this study uncovers a different mechanism whereby DR5 disrupts ER membrane homeostasis and induces hepatocyte pyroptosis by suppressing the key glycerophospholipid synthetic enzyme CHPT1. The identification of the DR5–metabolism–pyroptosis axis provides a different theoretical framework for understanding impaired injury recovery after massive PHx, nominates potential molecular targets for preventing and treating lethal PHLF, and lays a foundational basis for developing novel metabolism-targeted hepatoprotective therapeutics.

## Methods

This research complies with all relevant ethical regulations. The clinical study was approved by the Clinical Research Ethics Committee of the Third Affiliated Hospital of Sun Yat-sen University (Approval No.Ⅱ2025-316-1). All animal procedures were approved by the Institutional Animal Care and Use Committee (IACUC) of Sun Yat-Sen University (Approval No. SYSU-IACUC-2025-001978) and were conducted in compliance with the National Institutes of Health’s Guide for the Care and Use of Laboratory Animals (Approval No. #JENNIO-IACUC-2024-A062).

### Clinical specimen collection and data analysis

Clinical data and postoperative liver function parameters (alanine aminotransferase [ALT], aspartate aminotransferase [AST], albumin [ALB], prothrombin time [PT] and international normalized ratio [INR]) were retrospectively collected from the Electronic Medical Record System of the Third Affiliated Hospital, Sun Yat-sen University. All participants were informed about the study and provided written informed consent before inclusion.

### Animals and mouse models

Male wild-type C57BL/6J mice aged 8 weeks were obtained from Jiangsu Gempharmatech Biological Science and Technology Ltd (Nanjing, Jiangsu, China) and bred at the Sun Yat-sen University Laboratory Animals Center. All the experimental mice had free access to water and standard rodent chow and were housed in a specific pathogen-free (SPF) environment with a temperature of 25 °C, a humidity of 40–70%, and a 12 h light-dark cycle. The animal procedures were carried out in compliance with Chinese legislation regarding the use of experimental animals. For the PHx procedure, 8-week-old male mice were subjected to liver mass resection^48^ (The resection extents were defined as the left lateral lobe (30%), the left lateral plus half of the median lobe (50%), the left lateral and entire median lobes (70%), and the left lateral, median, and right lobes combined (90%)). Mice were anesthetized with 2% isoflurane (RWD, Shenzhen, China) via induction chamber and maintenance mask. Following a midline incision and exposure of the liver, the bases of the corresponding lobes were ligated with silk thread. Once hemostasis was secured, the lobes were excised. The abdomen was sutured, and the animals were monitored on a warming pad until they fully recovered. Mice were subsequently returned to cages and sacrificed at specific intervals after surgery, with untreated animals utilized as controls.

### Oil Red O staining

Frozen liver sections were fixed with 4% paraformaldehyde, pretreated briefly with 60% isopropanol, and stained using a freshly filtered Oil Red O working solution (Beyotime) for 10–15 min at room temperature in the dark. Following differentiation in 60% isopropanol and thorough washing with distilled water, the sections were counterstained with hematoxylin, mounted with an aqueous medium, and examined under a light microscope.

### Western blot analysis

Total protein was extracted from the liver tissue and THLE-2 cell line following the manufacturer’s protocol. The samples were then separated on 12% sodium dodecyl sulfate-polyacrylamide gels (Bio-Rad, Hercules, CA, USA) and transferred onto polyvinylidene fluoride membranes (Millipore, Bedford, MA, USA). The membranes were blocked with 5% skim milk powder at 25 °C for 1 h and incubated overnight at 4 °C with the following primary antibodies targeting the following proteins: NLRP3 (ab263899, Abcam), Gasdermin D (#39754, CST), IL-1β (ab283818, Abcam), Cleaved-Caspase-1 (#89332mouse, #4199human, CST), PERK (#3192, CST), P-PERK (AP1501, ABclonal), eIF2α (#5324, CST), P-eIF2α (AP0692, ABclonal), ATF4 (DF6008, Affinity), CHOP (AF6277, Affinity), DR5 (ab8416, Abcam), FXR/NR1H4 (#72105, CST), CHPT1 (A16137, ABclonal), TLR4(#14358, CST), NF-κB(#6956, CST), P-NF-κB(#3033, CST), Caspase-1(#83383, CST)and incubated at room temperature for 1 h with an appropriate secondary antibody conjugated to horseradish peroxidase. The protein signals were visualized with an ECL Western Blotting kit (P10300, NCM, Suzhou, China). ImageJ was used to calculate protein relative expression by plot every gel lane. All protein expression was normalized by loading control (β-Tubulin (#2146, CST), Histone H3 (#4499, CST), or GAPDH (#2118, CST)) (Detailed information is provided in Supplementary Table 1 of the Supplementary Information).

### Real-time quantitative polymerase chain reaction (RT-qPCR)

Total RNA was isolated using TRIzol (15596026, ThermoFisher), while cDNA synthesis was carried out using the Hiscript III RT Supermix for QPCR (+gDNA Wiper) kit, following the manufacturer’s instructions. Subsequently, the cDNA was quantified using a LightCycler 480 high-throughput real-time fluorescent quantitative PCR instrument. The mRNA expression level was normalized to 18 s rRNA level. Primers for qRT-PCR were synthesized by Tsingke Company in Wuhan (Detailed information is provided in Supplementary Table 1 of the Supplementary Information).

### Evaluation of liver injury

ALT and AST levels were measured as indicators of liver injury using an automatic analyzer (Antech Diagnostics). The liver tissues were fixed in 4% paraformaldehyde (PFA), embedded in paraffin, and sectioned to 4-μm thickness, then stained with hematoxylin and eosin (H&E). The damaged area was measured using Image J software. For every sample, five random fields were imaged.

### Cell culture and cell model

Culture flasks were pre-coated with Rat tail collagen type I (C8062, Solarbio) at a concentration of 50 μg/mL dissolved in 20 mM acetic acid. The THLE −2 cell line was obtained from ATCC. THLE-2 cell line was cultured in THLE-2 Cell Complete Medium (CM-0833, Procell) in a humidified atmosphere of 5% CO_2_ at 37 °C. Hypoxic conditions (H): hypoxic conditions (1% oxygen) for 4 h, and subsequently cultured in normal medium under normoxic conditions for 24 h. Palmitic acid (PA; Kunchuang Biotechnology) was added to the culture medium according to the corresponding concentration. Hypoxic+Palmitic acid (H + PA) cell model: THLE-2 initially placed in glucose-free DMEM (Gibco) were cultured under hypoxic conditions (1% oxygen) for 4 h, and subsequently cultured in normal medium supplemented with 0.25 mM PA under normoxic conditions for 24 h.

### Genetic modification

DR5 expression was silenced using a lentiviral CRISPR interference (CRISPRi) system, leveraging the recruitment of the KRAB transcriptional repressor to the target locus. THLE-2 cells were transduced with lentiviral particles generated from an all-in-one vector co-expressing dCas9-KRAB and human DR5-specific sgRNAs (targeting the TSS-proximal promoter region; sgRNA-1: 5’-GACAGGACTATAGCACTCAC-3’; sgRNA-2: 5’-GTGGACACAATCCCTCTGAG-3’). The overexpression vectors were verified by Sanger sequencing using the forward primer EF1α-F (5’-TCAAGCCTCAGACAGTGGTTC-3’) and the reverse primer seq-pLenti-SFB-R (5’-CCGGCTCCGCTGTCCATG-3’). The vector incorporated a Woodchuck Hepatitis Virus Posttranscriptional Regulatory Element (WPRE) to optimize transgene expression. Following transduction, stable cell pools were established through puromycin selection (2 μg/mL for 7 days). The KD/OE efficiency was validated by RT-qPCR and western blot, confirming a significant and sustained change.

### Enzyme-linked immunosorbent assay

IL-1β (Elabscience, Wuhan), IL-18 (Elabscience, Wuhan) and HMGB1 (Abclonal, Wuhan) levels in blood were quantified using commercially available ELISA kits, following the manufacturer’s protocols.

### Animal adeno-associated virus-8 (AAV8) injection and processing

Hepatocyte-specific DR5-KD in mice was achieved via tail vein injection of shRNA-carrying adeno-associated virus serotype 8 (mouse-AAV8-shDR5, 1 × 10^13^ viral genomes/mL). The shRNA sequence targeting DR5 in mice was GCACAGTCTGTAAGGAAGATA; For in vivo overexpression of DR5, the full-length coding sequence (CDS) of mouse Tnfrsf10b (NCBI Reference Sequence: NM_020275.4) was synthesized and cloned into an Adeno-associated virus (AAV) vector under the control of the TBG promoter. AAV viral vectors were generated by PackGene Biotech (Guangzhou, China). After 4 weeks, the PHx model was established with indicated AAV8-treated mice.

### Chromatin immunoprecipitation (ChIP) qPCR

The ChIP assays were performed using the ChIP Assay Kit (17-10086, Merck Millipore) following the manufacturer’s protocol. Briefly, over 1 × 10^7^ THLE-2 cells were cross-linked with 1% formaldehyde at 37 °C for 10 min, followed by quenching with 1X Glycine Solution at room temperature for 5 min. After two washes with PBS containing 1 mM PMSF, the cells were lysed in SDS buffer with 1 mM PMSF on ice (10 min). Chromatin was fragmented by sonication (10% intensity, 5-s pulses) and centrifuged (4 °C, 14,000 *g*, 5 min). The supernatant was diluted with 2 mL ChIP Dilution Buffer containing 1 mM PMSF. While 10% of each sample was reserved as input, the remainder was immunoprecipitated overnight at 4 °C with either NR1H4 antibody or control IgG. After centrifugation (4 °C, 1000 *g*, 1 min), the pellets were sequentially washed with Low Salt, High Salt, and LiCl Immune Complex Wash Buffers, followed by TE Buffer (4 °C, 5 min each). Finally, the precipitated DNA was purified for subsequent ChIP-qPCR analysis using *Chpt1* promoter-specific primers (Supplementary Table 1), with mouse GAPDH serving as the negative control.

### Immunofluorescence staining

Cells were washed with PBS and fixed with 4% paraformaldehyde (PFA) for 30 min at room temperature. After permeabilization with 0.1% Triton X-100 for 10 min, followed by blocking in 10% bovine serum albumin (BSA) in PBS for 30 min. Subsequently, the samples were incubated with primary antibodies against DR5 and NR1H4 overnight at 4 °C. Following three washes with PBS, the cells were incubated with secondary antibodies, including Alexa Fluor 546-conjugated goat anti-mouse IgG (Invitrogen, Carlsbad, CA, USA) and Alexa Fluor 488-conjugated goat anti-rabbit IgG (Invitrogen, Carlsbad, CA, USA), for 1 h at room temperature in the dark. Nuclei were counterstained with DAPI. Fluorescence images were captured using a laser scanning confocal microscope (Zeiss LSM 880).

### FFA concentration measurement

The levels of free fatty acids (FFA) in liver tissues were quantified using a commercial Free Fatty Acid Assay Kit (Solarbio, Beijing, China), following the manufacturer’s instructions. Briefly, liver tissue samples were weighed and homogenized in the extraction buffer on ice. The homogenates were centrifuged at 8000 × *g* for 10 min at 4 °C, and the supernatant was collected for analysis. The extraction solution was mixed with the working reagents, and the reaction mixture was incubated at room temperature for 10 min. The absorbance was measured at 550 nm using a microplate reader. The FFA concentration was calculated based on a standard curve and normalized to the tissue weight of the samples.

### ScRNA-seq

#### 70%PHx single-cell RNA sequencing

The dataset CNP0002310^31^ was sourced from CNGB. The h5ad file was first converted to a Seurat object, preserving the original author’s cell type annotations, which were set as the default identity. Rapid quality control was performed, followed by dimensionality reduction and visualization (prioritizing pre-computed Umap embeddings when available) and validation using canonical marker genes. Additional analyses included evaluation of temporal changes in cell type proportions across time points and comprehensive identification of marker genes for all cell types.

#### 90%PHx single-cell RNA sequencing

##### Preparation of Single-Cell Suspensions

Freshly harvested liver tissues were transferred into Petri dishes containing pre-chilled, RNase-free, calcium- and magnesium-free 1× PBS. The tissues were minced into small fragments (approximately 0.5 mm²) and washed to remove residual blood, lipids, and other debris. The washed tissue fragments were dissociated using an enzyme cocktail in a 37 °C water bath for 30–60 min. Digestion was terminated by adding RPMI 1640 medium (Gibco) supplemented with 2% fetal bovine serum (FBS). The resulting suspension was filtered through a 70-µm cell strainer and centrifuged at 300 × *g* for 5 min at 4 °C. After discarding the supernatant, the cell pellet was resuspended. To remove erythrocytes, cells were treated with 3 mL of Red Blood Cell Lysis Buffer (Solarbio, Beijing, China) on wet ice or at room temperature for 2–10 min. Following lysis, the suspension was centrifuged at 300 × *g* for 5 min at 4 °C. The cell pellet was washed twice by resuspending in 1× PBS containing 0.04% bovine serum albumin (BSA) followed by centrifugation at 300 × *g* for 3 min at 4 °C. Finally, the cells were resuspended in 1× PBS (0.04% BSA). Cell viability was assessed using AO/PI staining, with a viability threshold of >85% required for downstream processing. Cell counting was performed using a Countess II Automated Cell Counter. When necessary, cell suspensions were optimized using dead cell or debris removal kits (Miltenyi Biotec) to ensure high sample quality.

##### scRNA-seq Library Construction and Sequencing

Single-cell RNA-seq libraries were constructed using the DNBelab C4 system. Cell capture, cDNA amplification, and library preparation were performed using the DNBelab C Series High-throughput Single-cell RNA Library Preparation Kit V3.0 (MGI Tech) following the manufacturer’s protocol, with a target capture of approximately 10,000 cells per sample. The concentration of the libraries was quantified using a Qubit Fluorometer, and fragment integrity was assessed using a Qsep Bio-Fragment Analyzer. The final libraries were sequenced on a DNBSEQ-T7 platform (MGI Tech) using a paired-end 150 bp (PE150) strategy. These services were provided by Guangzhou Angte Biotechnology Co., Ltd. (Guangzhou, China).

##### Single-cell pyroptosis score inference using UCell

For single-cell-resolution inference of pyroptosis activity, we employed the UCell R package (version 2.6.2), leveraging its distinct non-parametric scoring capability based on the Mann-Whitney U statistic^49^. Raw count matrices from the integrated single-cell dataset were utilized as input, encompassing all identified cell types across different time points (D1-D3). A pre-defined pyroptosis-related gene signature (detailed in Source Data-Cell death-related gene) was curated for the analysis. For each individual cell, gene-wise ranks within the signature were computed based on their relative expression levels. The final pyroptosis score reflected the collective enrichment of the pyroptosis gene set. This UCell-based score provided a quantitative, robust assessment of pyroptosis activity that was relatively independent of dataset size or sequencing depth, enabling comparisons of specific cell subsets at distinct injury stages.

### RNA-seq

Total RNA was extracted from the mouse liver using TRIzol (15596026, Thermo Fisher). Bulk RNA-seq libraries were prepared using MGIEasy RNA Library Prep Set (MGI, 1000006384) according to the manufacturer’s instructions and sequenced on an MGISEQ-2000 sequencer at CNGB with paired-end 100 bp. RNA-seq data for gene expression were quantified in an RSEM-based pipeline. DEGs were determined by DESeq2 (v.1.30.1) and defined as having an absolute fold change of >1.5 and *P* value of <0.05. Functional annotation was performed with Metascape (v.3.5)84.

### Drug

Phosphatidylcholine (PC) Supplementation: To enrich cellular membranes with PC, a bovine serum albumin (BSA)-complexed lipid supplementation method. Briefly, L-α-phosphatidylcholine (P5394, Sigma-Aldrich) was first dissolved in chloroform and dried under a stream of nitrogen gas to form a thin lipid film. The film was resuspended in a 10% (w/v) fatty acid-free BSA solution (prepared in PBS) and incubated at 37 °C with continuous vortexing for 1 h until a clear PC-BSA complex was formed. The complex was then sterile-filtered (0.22 μm) and added to the cell culture medium to achieve a final PC concentration of 50 μM^50^. Cells were incubated for 24 h to allow for metabolic incorporation into the membrane bilayers; 500 mg/kg for mice in vivo, i.p. β-oxidation agonist: WY-14643(HY-16995, MCE; 100 mg/kg for mice in vivo, i.p.)^51^; β-oxidation inhibitor: Etomoxir(ETO, S8244, Selleck; 10 mg/kg for mice in vivo, i.p.)^52^; FXR agonist: GW4064(HY-50108, MCE; 10 μm^53^; 30 mg/kg for mice in vivo, i.p.^53,54^); FXR inhibitor: (Z)-Guggulsterone(Z-GS)(HY-110066, MCE; 20 μm; 30 mg/kg for mice in vivo, i.p.)^55^ (Detailed information is provided in Supplementary Table 1 of the Supplementary Information).

### Proteomics analysis by LC-MS/MS

A total of 24 samples, comprising *n* = 6 biologically independent replicates per group, were analyzed. Tissue samples were ground into powder in liquid nitrogen. The powder was lysed in lysis buffer containing 8 M urea, 1 mM PMSF, and 2 mM EDTA, followed by sonication on ice for 5 min. After centrifugation at 15,000 × *g* for 10 min at 4 °C, the protein concentration in the supernatant was determined using a BCA assay kit. For digestion, 100 μg of protein was adjusted to a volume of 200 μL with 8 M urea. Samples were reduced with 5 mM dithiothreitol (DTT) for 45 min at 37 °C and alkylated with 11 mM iodoacetamide (IAA) for 15 min at room temperature in the dark. The samples were then diluted with 800 μL of 25 mM ammonium bicarbonate and digested with trypsin (Promega, Madison, WI, USA) overnight at 37 °C. The digested peptides were acidified to pH 2–3 with 20% trifluoroacetic acid (TFA), desalted using C18 resin (Millipore, Billerica, MA, USA), and quantified using the Pierce™ Quantitative Peptide Assay Kit (Thermo Fisher Scientific).

Peptides were separated using a Vanquish Neo UHPLC system (Thermo Fisher Scientific). A trap-and-elute strategy was employed using a PepMap Neo Trap Cartridge (300 μm × 5 mm, 5 μm) and an Easy-Spray™ PepMap™ Neo UHPLC analytical column (150 μm × 15 cm, 2 μm). The mobile phases consisted of 0.1% formic acid in water (A) and 0.1% formic acid in acetonitrile (B). The flow rate was set to 2.5 μL/min with an effective gradient of 6.9 min (total runtime 8 min). After LC separation, the peptides were analyzed by an Orbitrap Astral high-resolution mass spectrometer (Thermo Fisher Scientific) in Data-Independent Acquisition (DIA) mode. The instrument operated in positive ion mode with a precursor scan range of 380–980 m/z and a resolution of 240,000 (at 200 m/z). The normalized AGC target was 500% with a maximum injection time (IT) of 5 ms. For MS2 DIA acquisition, 299 scan windows were used with an isolation window of 2 Th, HCD collision energy of 25%, normalized AGC target of 500%, and maximum IT of 3 ms.

Raw MS data were processed using DIA-NN software (version 1.8.1) with a library-free approach. A spectral library was generated in silico from the mouse proteome database (UniProt UP000000589, 54,910 sequences) using deep learning algorithms. For the database search, the protease was set to trypsin with a maximum of two missed cleavages. Carbamidomethylation of cysteine was set as a fixed modification, while oxidation of methionine and N-terminal acetylation were set as variable modifications. The minimum peptide length was set to 7 amino acids. Mass tolerances for precursor and fragment ions were determined automatically by DIA-NN. The Match Between Runs (MBR) option was enabled. The False Discovery Rate (FDR) was controlled at <1% at both the precursor and protein levels. Protein identification required a minimum of 1 unique peptide (Detailed information is provided in Supplementary Data 1).

#### Lipidomics analysis by LC-MS/MS

Prior to extraction, tissue samples (20 mg) were snap-frozen in liquid nitrogen and stored at −80 °C without undergoing any freeze-thaw cycles to ensure lipid integrity. Samples were ground in liquid nitrogen and extracted with 1 mL of solvent mixture (MTBE:MeOH = 3:1, v/v) containing a mixture of internal standards. After vortexing for 15 min and adding 200 μL of ultrapure water, the mixture was centrifuged at 12,000 rpm for 10 min. The upper organic layer (200 μL) was collected and dried under vacuum. The residue was reconstituted in 200 μL of acetonitrile/isopropanol (ACN:IPA = 1:1, v/v) for LC-MS/MS analysis.

Lipid profiling was performed using an ExionLC AD UPLC system coupled with a QTRAP® 6500+ mass spectrometer (Sciex, Framingham, MA, USA). Separation was achieved on a Thermo Accucore™ C30 column (2.6 μm, 2.1 mm × 100 mm) at 45 °C. The mobile phases were (A) acetonitrile/water (60:40, v/v) and (B) acetonitrile/isopropanol (10:90, v/v), both containing 0.1% formic acid and 10 mM ammonium formate. The gradient elution was performed at a flow rate of 0.35 mL/min. The ESI source parameters were set as follows: source temperature 500 °C; ion spray voltage 5500 V (positive) and −4500 V (negative); gas settings GS1 45 psi, GS2 55 psi, and curtain gas 35 psi.

Data acquisition and processing were conducted using Analyst 1.6.3 software and the MetWare database (MetWare Biotechnology Co., Ltd., Wuhan, China). Lipid identification was based on the proprietary MWDB (Metware database), utilizing retention time (RT) and specific precursor-to-product ion transitions for structural confirmation. Lipid quantification was performed in Multiple Reaction Monitoring (MRM) mode. Quantitative values were determined using a relative response method based on the area ratio of the target lipid to the corresponding internal standard. The absolute content (nmol/g) was calculated using the formula: *X* = 0.001 * *R* * *c* * *F* * *V*/*m*, where R is the peak area ratio of the target lipid to the internal standard, *c* is the internal standard concentration, *F* is the relative response factor, *V* is the extraction volume, and *m* is the sample weight (Detailed information is provided in Supplementary Data 2). To evaluate the stability and repeatability of the LC-MS/MS analytical system, a mixed QC sample was prepared by pooling equal aliquots from all experimental samples. During the instrumental analysis, one QC sample was inserted into the analytical sequence after every 12 test samples.

#### K-Means clustering analysis

To elucidate the dynamic evolutionary patterns of lipid and protein profiles during the acute injury phase following PHx, K-Means clustering of temporal expression profiles^56,57^ was performed using the R stats package (version 4.1.2). Expression matrices comprising significantly differentially expressed lipids and proteins across Control (C) and early post-PHx time points (D1, D2, and D3) were utilized as input features. The optimal number of clusters (K) was determined by integrating the Elbow method with biological interpretability. The algorithm partitioned metabolites and proteins into distinct subclasses based on the Euclidean distance of their multi-time-point expression trajectories, clustering features with highly similar dynamic patterns. Iterative calculations of cluster centroids were executed until convergence criteria were met. This temporal clustering approach enabled the identification of specific expression modules, facilitating the precise characterization of disease and providing a comprehensive systems biology context for post-operative injury.

### Nuclear and cytoplasmic fractionation

Nuclear and cytoplasmic proteins were extracted using the Nuclear and Cytoplasmic Protein Extraction Kit (Cat# P0028, Beyotime Biotechnology, Shanghai, China) according to the manufacturer’s protocol. Briefly, cells were harvested, washed with ice-cold PBS, and resuspended in Cytoplasmic Extraction Reagent A supplemented with 1 mM PMSF. After incubation on ice for 15 min, Cytoplasmic Extraction Reagent B was added, and the mixture was vigorously vortexed for 5 s and incubated on ice for 1 min. The samples were then centrifuged at 12,000 × *g* for 5 min at 4 °C. The resulting supernatant (cytoplasmic fraction) was collected. The remaining pellet was resuspended in Nuclear Extraction Reagent containing PMSF and vortexed intermittently for 30 min on ice. Following centrifugation at 16,000 × *g* for 10 min at 4 °C, the supernatant (nuclear fraction) was collected. Protein concentrations were determined using a BCA Protein Assay Kit.

### Co-Immunoprecipitation (Co-IP)

Cell lysates were prepared using IP lysis buffer and incubated with specific antibodies against DR5/CHPT1 or control IgG overnight at 4 °C. The immune complexes were captured using Protein A/G Magnetic Beads (Elabscience, Wuhan, China) for 2 h. After extensive washing with a magnetic separator, the bound proteins were eluted by boiling in SDS sample buffer and subjected to Western blot analysis to detect the interaction with CHPT1/DR5.

### Cell Counting Kit-8 assay (CCK-8)

Adherent primary hepatocytes were plated in 96-well plates, and each group was spread with 3–5 holes. Each hole was filled with 200 μL medium or configured drugs. During the detection, the culture medium was replaced, 10 μL CCK-8 (KGA317-2, KeyGEN) and 100 μL culture medium (Gibco) were added to each well, and incubated at 37 °C for 2 h. Finally, the absorbance at 450 nm was measured using a microplate reader.

### Machine learning models

#### Machine learning model objective and feature selection

To identify key regulators of pyroptosis and lipid metabolism, a binary classification machine learning framework was constructed^23,58^. The primary objective was to screen for genes that optimally discriminate between pre-operative control samples and post-hepatectomy injury samples across different surgical stages, and to rank these genes based on their predictive importance. The output variable was defined as a binary label: the positive class (1) represented disease states (post-partial hepatectomy [PHx] samples at D1, D2, or D3 after 70% PHx), while the negative class (0) represented pre-surgery controls (C). The input features consisted of the expression profiles of 26 candidate genes, derived from the intersection of differentially expressed genes (DEGs) from bulk RNA-seq, single-cell RNA-seq (scRNA-seq), Weighted Gene Co-expression Network Analysis (WGCNA) modules, and well-defined pyroptosis-related genes. A parallel evaluation utilizing a subset of 20 candidate genes was also performed.

#### Training, validation, and testing strategy

Model training was conducted using an internal bulk RNA-seq dataset comprising 36 samples (*n* = 36 paired samples derived from 18 mice). To rigorously evaluate the generalizability of the models, four independent external datasets (GSE151309, GSE20426, GSE181476, and CNP0002310) were utilized as independent test sets. For single-cell datasets within this testing cohort, data were converted to pseudo-bulk expression profiles prior to model evaluation. During the training phase, a 5-fold cross-validation (CV) strategy was employed, wherein the training data was iteratively partitioned into 80% for model fitting and 20% for internal validation. Prior to downstream classification, Lasso (Least Absolute Shrinkage and Selection Operator) regression (alpha=1, via 5-fold CV) was applied to select an optimal subset of informative features and reduce dimensionality.

#### Algorithms and hyperparameter tuning

A comprehensive panel of 15 machine learning algorithms was implemented. For algorithms necessitating hyperparameter optimization, grid search strategies were executed within the cross-validation framework. Specifically, the Multi-Layer Perceptron (MLP) neural network was tuned for batch size, learning rate, dropout rate, and cutoff values. Extreme Gradient Boosting (XGBoost) models were optimized across learning rates (eta: 0.025-0.3), maximum depth (2–6), and iterations (nrounds: 50–1000). Support Vector Machine (SVM) models evaluated linear, polynomial, and radial kernels alongside cost/gamma parameters. Gradient Boosting Machine (GBM) parameter grids included interaction depth, number of trees, shrinkage, and minimum observations in terminal nodes. Random Forest models were tuned by iterating mtry values corresponding to the 25th, 50th, and 75th percentiles of the feature count, while k-Nearest Neighbors (KNN) evaluated k values ranging from 1 to 5. Other algorithms, including Logistic Regression, Linear Discriminant Analysis (LDA), Quadratic Discriminant Analysis (QDA), Ridge Regression, Elastic Net, Decision Trees, and Naive Bayes, were applied using default configurations or evaluated across standard probability thresholds (0.25, 0.5, 0.75).

#### Performance evaluation and model interpretability

Model performance across both the internal training cohort and external test sets was quantified using Accuracy, Recall (Sensitivity), and F1-score. To ensure biological interpretability and pinpoint key regulatory genes, feature importance was extracted and aggregated from multiple high-performing models. This included the Mean Decrease in Gini index from the Random Forest model, the gain metrics from the XGBoost algorithm, and the non-zero coefficients derived from the Lasso regression. The candidate genes were subsequently ranked based on their integrated importance scores, consistently identifying DR5 among the top three key regulators across the utilized methodologies. Data: GSE151309 (data2), GSE20426 (data3), GSE181476 (data4), and CNP0002310 (data5).

### Multiplex immunofluorescence (mIF) staining

Multiplex immunofluorescence (mIF) staining was performed on formalin-fixed, paraffin-embedded liver sections using a 4-color Multiplex Fluorescent Immunohistochemistry Kit (Servicebio, Wuhan, China). Following deparaffinization and microwave-based antigen retrieval in citrate or EDTA buffer, endogenous peroxidase was blocked, and sections were incubated with primary antibodies overnight at 4 °C. A sequential staining protocol was employed based on Tyramide Signal Amplification (TSA) technology, where each cycle consisted of HRP-conjugated secondary antibody incubation, TSA-fluorophore reaction, and heat-induced antibody stripping. Target proteins were grouped into distinct panels on serial sections, utilizing antibodies against CD206 (#24595, CST), iNOS (ab3523, Abcam), F4/80 (ab300421, Abcam), CD3 (ab135327, Abcam), CD19 (ab245235, Abcam), NCR-1 (ab233558, Abcam), DR5 (ab8416, Abcam), HMGB1 (ab79823, Abcam), HNF4α (ab201460, Abcam), CD31 (ab281583, Abcam), CD11b (ab133357, Abcam), GSDMD-N (A24059, ABclonal) and NR1H4 (#72105, CST, Danvers, MA, USA), as well as CHPT1 (A16137, ABclonal). Finally, nuclei were counterstained with DAPI, and images were captured using a laser scanning confocal microscope (Zeiss LSM 880) (Detailed information is provided in Supplementary Table 1 of the Supplementary Information).

### Transmission electron microscopy (TEM)

The anesthetized mice were perfused with 0.9% saline, and 1 mm^3^ liver tissue blocks were obtained. Next, the fresh tissue blocks were immersed into fixative for TEM (Servicebio) at 4 °C for 4 h. They were then treated with 1% OsO4 in 0.1 mol/L PBS for 2 h at room temperature, followed by a dehydration process with gradient alcohol. Subsequently, the sections were embedded and underwent baking in an oven at 60 °C for 48 h and cut into ultrathin sections (60 nm) using an ultramicrotome. Finally, the ultrastructure of the tight junctions of the liver was observed using a transmission electron microscope (HITACHI).

### Scanning electron microscopy (SEM)

Liver tissues were cut into 5 mm^3^ blocks, washed with PBS, and fixed in EM fixative for 2 h at room temperature (stored at 4 °C). Samples were washed in 0.1 M PBS (pH 7.4) and post-fixed with 1% OsO_4_ for 1–2 h in the dark. Then, tissues were dehydrated in a graded ethanol series (30–100%) and substituted with isoamyl acetate. Samples were critical-point dried, mounted on conductive carbon tape, and sputter-coated with gold for 30 s prior to SEM (HITACHI) imaging.

### Statistics and reproducibility

For the in vivo experimental models, male C57BL/6J mice of similar age and body weight were randomly allocated into experimental groups (e.g., Control, D1, D2, and D3) prior to surgical procedures. For the multi-omics (proteomics and lipidomics) analyses, a sample size of *n* = 6 biologically independent animals per group was used (a total of 24 samples). Data collection and analysis were not performed blind to the conditions of the experiments. All experiments were performed independently to ensure reproducibility. Specifically, for all representative micrographs, western blots, and flow cytometry plots presented in this study, the displayed images are representative of at least three independent experiments that yielded similar results.

All quantitative data are presented as the mean ± SD, mean ± SEM or median with IQR, as specified in the respective figure legends. Statistical analyses were performed using GraphPad Prism (version 8.0.1; GraphPad Software, La Jolla, CA, USA) and R software (version 4.2.0). Data normality was assessed using the Shapiro-Wilk test. For data that passed the normality test, comparisons between two independent groups were performed using a two-tailed unpaired Student’s *t* test, while comparisons between paired groups were analyzed using a two-tailed paired Student’s *t* test. Comparisons among three or more groups were evaluated using one-way analysis of variance (ANOVA), followed by Dunnett’s or Tukey’s multiple-comparisons test. For non-normally distributed data, comparisons between two groups were performed using the nonparametric Mann-Whitney U test. For multi-omics functional enrichment analyses, one-sided hypergeometric tests were used, with *P* values adjusted for multiple comparisons using the Benjamini-Hochberg method to control the false discovery rate (FDR). Exact *P* values, specific statistical methods, and precise n values for each experiment are detailed in the respective figure legends and figures. Source data are provided in the Source Data file.

### Reporting summary

Further information on research design is available in the Nature Portfolio Reporting Summary linked to this article.

## Supplementary information

Supplementary information

Description of Additional Supplementary Files

Supplementary Data 1

Supplementary Data 2

Reporting Summary

Transparent Peer Review file

## Source data

Source data

## Data Availability

All RNA-seq and ScRNA-seq data have been deposited in the Sequence Read Archive (SRA) under accession number PRJNA1513559 and PRJNA1490015 (https://www.ncbi.nlm.nih.gov/sra/?term=PRJNA1513559; https://www.ncbi.nlm.nih.gov/sra/?term=PRJNA1490015). Proteomics data have been deposited to the ProteomeXchange Consortium via the iProX partner repository^59,60^ with the dataset identifier PXD080626 (http://proteomecentral.proteomexchange.org/cgi/GetDataset?ID=PXD080626; https://www.iprox.cn/page/project.html?id=IPX0018189000). Lipidomics generated in this study have been deposited to MetaboLights repository^61^ with the study identifier MTBLS14980. All data supporting the findings of this study are included in the main text, the Supplementary Information, or the Source Data file. Source data are provided with this paper.

## Code availability

All R scripts utilized for WGCNA, machine learning, multi-omics integration, and single-cell transcriptomic analyses are publicly accessible via GitHub (https://github.com/tys980915/code.git), accompanied by a README guide to facilitate the reproduction of the analytical pipeline. To ensure long-term preservation and citability, this repository has also been archived on the Zenodo platform (https://doi.org/10.5281/zenodo.21936844).
