# Supplementary material for: Multi-omics reveals the DR5-FXR-CHPT1 axis governs hepatocyte pyroptosis via glycerophospholipid remodeling after major partial hepatectomy in male mice
Source: Nat Commun. 2026 Oct 7;17:11423. doi: 10.1038/s41467-026-78113-8 (PMC13737082; doi:10.1038/s41467-026-78113-8)
Supplement: Supplementary file 1 [file 41467_2026_78113_MOESM1_ESM.pdf]

Supplementary Information

Multi-omics reveals the DR5-FXR-CHPT1 axis governs hepatocyte pyroptosis via glycerophospholipid remodeling after major partial hepatectomy in male mice

Correspondence to: Linsen Ye, ye_linsen@163.com; Zenan Yuan, yuanzn5@mail.sysu.edu.cn; Changchang Jia, jiachch3@mail.sysu.edu.cn; Hua Li, lihua3@mail.sysu.edu.cn.

This PDF file includes:

Figs. S1 to S7

Supplementary table 1 (Key resources table)

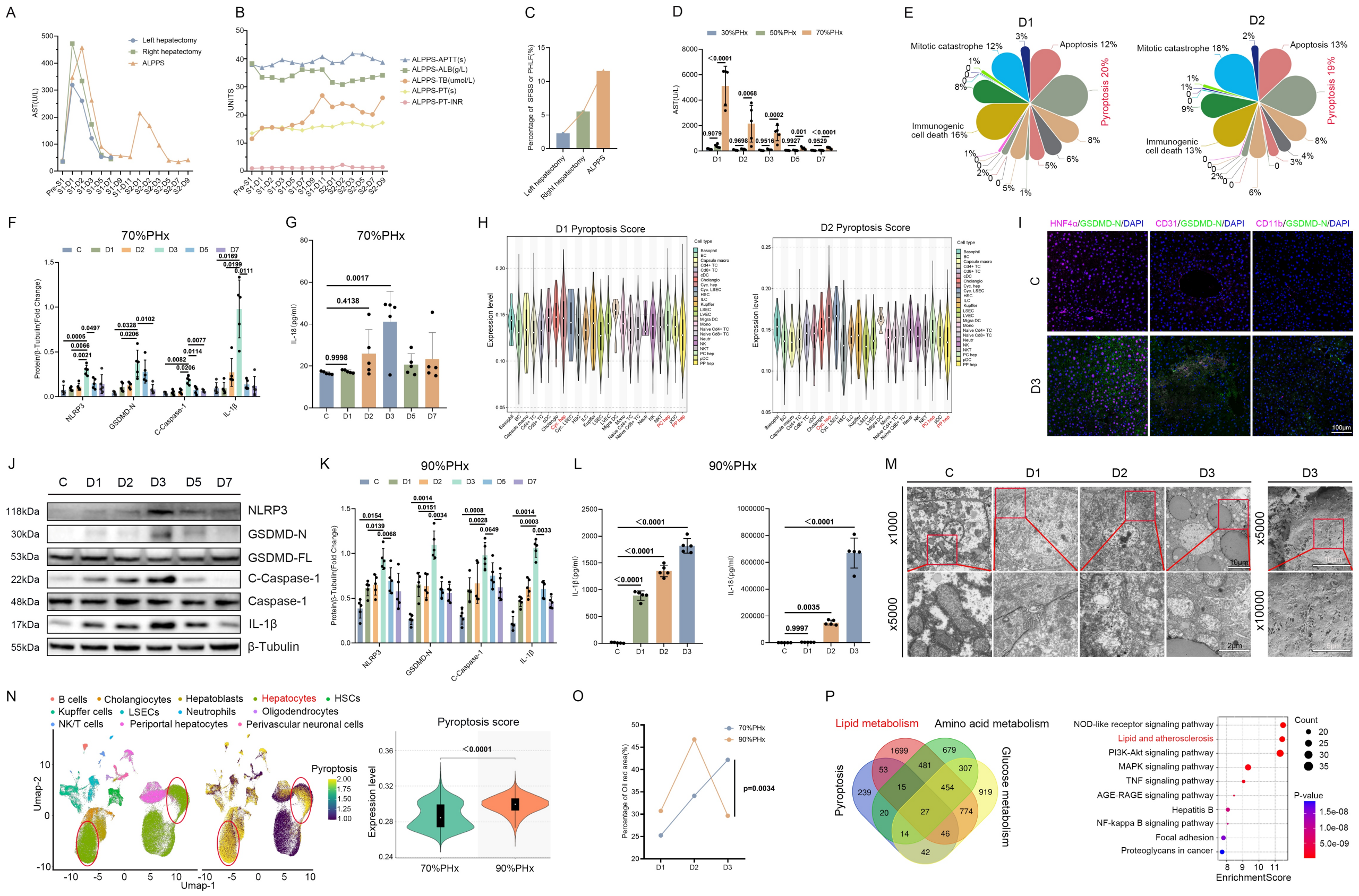

Supplementary Figure 1. Prediction and validation of cell death modalities following major PHx.

A: Serum AST levels following anatomical left hemihepatectomy (n=174 patients), anatomical right hemihepatectomy (n=145 patients) and ALPPS (n=52 patients). B: The level of APTT, ALB, PT, TB, and PT-INR following ALPPS (n=52 patients). C: Comparison of the incidence rates of SFSS or PHLF following anatomical left hemihepatectomy, anatomical right hemihepatectomy and ALPPS. D: Serum AST levels in mice with different resection volumes at various time points following 70% PHx (n=5 mice per group. Data for D1, D3, D5 and D7 are presented as mean ± SD. Statistical significance was analyzed using one-way ANOVA followed by Tukey’s multiple-comparisons test. Data for D2 are presented as mean ± SD. Statistical significance was determined using Welch's one-way ANOVA followed by

Games-Howell's multiple comparisons test). E: Classification of cell death patterns derived from RNA-seq DEGs of liver tissue at D1 and D2 following 70% PHx. F: Quantitative statistical analysis of protein expression levels corresponding to Figure 1D (n=5 mice per group. Data are presented as mean ± SD. Statistical significance was determined using ordinary one-way ANOVA followed by Dunnett's multiple comparisons test). G: Serum levels of IL-18 cytokines measured by ELISA following 70% PHx (n=5 mice per group. Data are expressed as mean ± SD. Statistical significance was analyzed using one-way ANOVA followed by Dunnett's multiple comparisons test). H: scRNA-seq analysis showing pyroptosis scores at D1 and D2 after 70% PHx across distinct hepatic cellular clusters by using the UCell method (CNP0002310). I: Representative immunofluorescence co-staining of the major cell types undergoing pyroptosis in liver tissues following 70% PHx (n=3 independent experiments). J: Representative western blot images of pyroptosis-related proteins in liver tissue following 90% PHx (n=5 independent experiments). K: Quantitative statistical analysis of protein expression levels corresponding to Supplementary Figure 1J (n=5 mice per group. Data are expressed as mean ± SD. Statistical significance was analyzed using one-way ANOVA followed by Dunnett's multiple comparisons test). L: Serum levels of IL-1β and IL-18 cytokines measured by ELISA following 90% PHx (n=5 mice per group. Data are expressed as mean ± SD. Statistical significance was analyzed using one-way ANOVA followed by Tukey's multiple-comparisons test). M: Representative Transmission Electron Microscopy (TEM) and Scanning Electron Microscopy (SEM) images of liver tissue following 90%

PHx (Scale bar=10 μm. n=3 independent experiments). N: Umap of scRNA-seq from the remnant liver at D3 of 90% PHx, (left panel) Global cell clustering colored by major cell types; (right panel) Umap highlighting cells with enriched PRG signatures; GSVA scores of PRGs sets in hepatocytes from 70% PHx and 90% PHx. O: Quantitative statistical analysis of Oil Red O staining corresponding to Figure 1H. P: Venn diagram identifying upregulated PRGs involved in lipid, glucose, and amino acid metabolism in the 90% PHx scRNA-seq relative to the 70% PHx scRNA-seq. Q: KEGG enrichment analysis of upregulated PRGs in the 90% PHx scRNA-seq compared to the 70% PHx scRNA-seq. Abbreviations: C, Control; AST, Aspartate aminotransferase; ALPPS, Associating liver partition and portal vein ligation for staged hepatectomy; PHx, Partial hepatectomy; RNA-seq, RNA sequencing; DEGs: Differentially expressed genes; Supplementary Figure 1E (D1): Pyroptosis, 20%; Immunogenic cell death, 16%; Mitotic catastrophe, 12%; Apoptosis, 12%; Anoikis, 8%; Ferroptosis, 8%; Autophagy , 6%; Necroptosis, 5%; Parthanatos, 5%; MPT (mitochondrial permeability transition-driven necrosis), 3%; Lysosome-dependent cell death, 2%; Disulfidptosis, 1%; Cuproptosis, 1%; NETosis, 1%; Entosis, 0%; Paraptosis, 0%; Entotic cell death, 0%; Methuosis, 0%; Alkaliptosis, 0%; Oxeiptosis, 0%; Netotic cell death, 0%. Supplementary Figure 1E (D2): Pyroptosis, 19%; Mitotic catastrophe, 18%; Immunogenic cell death, 13%; Apoptosis, 13%; Anoikis, 9%; Ferroptosis, 8%; Parthanatos, 6%; Autophagy (autophagic cell death), 4%; Necroptosis, 3%; MPT (mitochondrial permeability transition-driven necrosis), 2%; Lysosome-dependent cell death, 2%; Disulfidptosis, 1%; Entosis, 1%; Cuproptosis,

0%; NETosis, 0%; Paraptosis, 0%; Alkaliptosis, 0%; Oxeiptosis, 0%; Methuosis, 0%; Entotic cell death, 0%; Netotic cell death, 0%. All p values are shown in the figure, Source data are provided as a Source Data file.

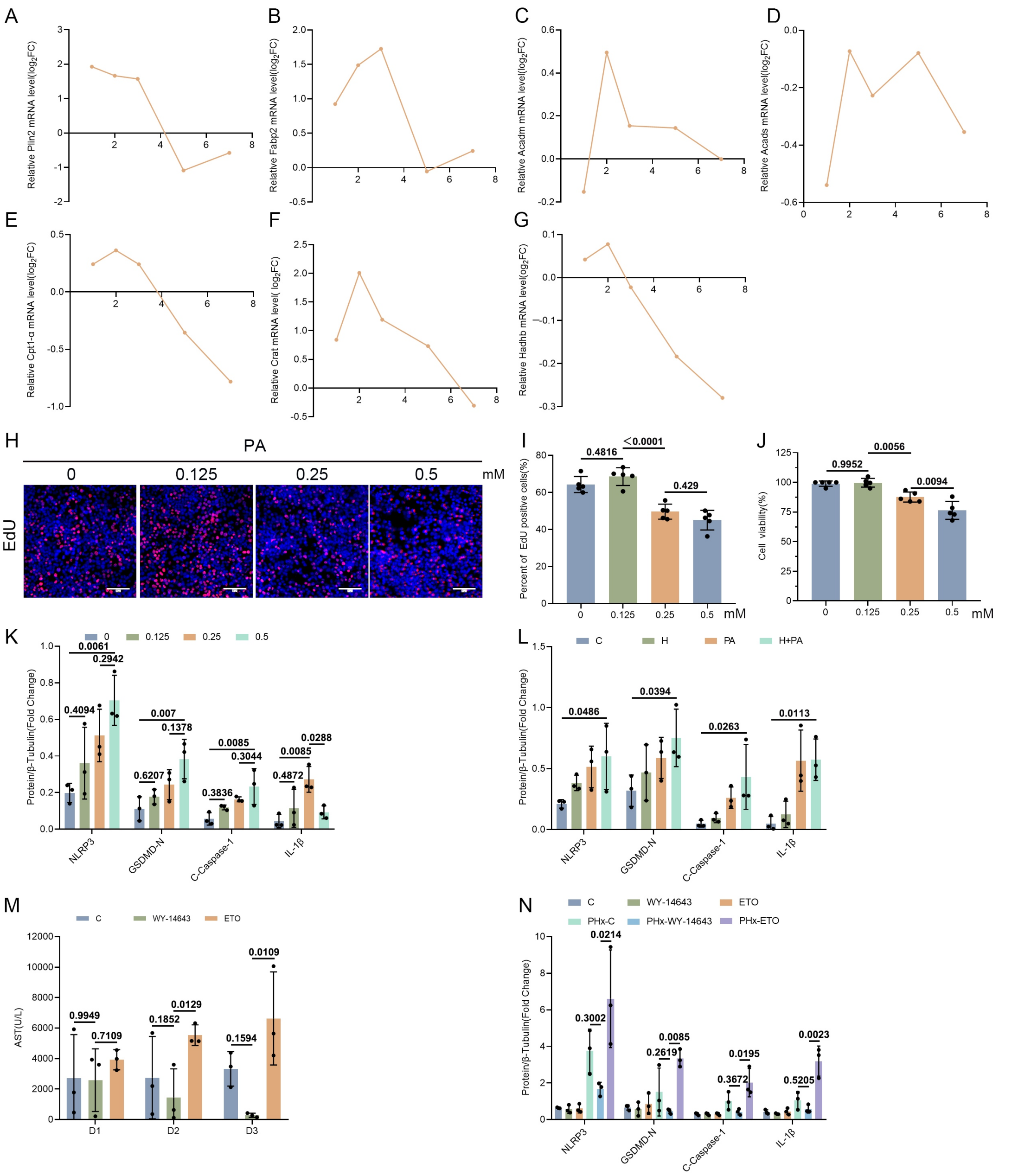

Supplementary Figure 2. Temporal expression patterns of lipid metabolism-related genes following 70% PHx and their effects on hepatocytes.

A-G: Line charts showing the dynamic changes in mRNA levels of lipid metabolism-related genes: *Plin2* (A), *Fabp2* (B), *Acadm* (C), Acads (D), Cpt1-α (E), *Crat* (F), and *Hadhb* (G) at various postoperative time points. H: Representative immunofluorescence images of EdU incorporation in hepatocytes treated with different concentrations of palmitic acid (PA: 0, 0.125, 0.25, and 0.5 mM) for 24 h

(Scale bar=20 μm, n=3 independent experiments). I: Statistical quantification of the percentage of EdU-positive cells in Supplementary Figure 2H (n=5 independent experiments). J: Hepatocyte viability assessed by CCK-8 assay following treatment with different concentrations of PA (n=5 independent experiments). K: Quantitative statistical analysis of protein expression levels corresponding to Figure 2D (n=3 independent experiments). L: Quantitative statistical analysis of protein expression levels corresponding to Figure 2G (n=3 independent experiments). M: Serum AST levels in each group at D1, D2, and D3 (n=3 per group). N: Quantitative statistical analysis of the western blot data corresponding to Figure 2M (n=3 per group). Abbreviations: Plin2, Perilipin 2; Fabp2, Fatty acid-binding protein 2; Acadm, Medium-chain acyl-CoA dehydrogenase; Acads, Short-chain acyl-CoA dehydrogenase; Cpt1-α, Carnitine palmitoyltransferase 1α; Crat, Carnitine O-acetyltransferase; Hadhb, Hydroxyacyl-CoA dehydrogenase beta subunit; EdU, 5-Ethynyl-2′-deoxyuridine; CCK-8, Cell Counting Kit-8; PA, Palmitic acid. Data are expressed as mean ± SD. Statistical significance was analyzed using one-way ANOVA followed by Dunnett's multiple comparisons test (Supplementary Figure 2I-J, K for GSDMD-N, L-N). Statistical significance was analyzed using one-way ANOVA followed by Tukey's multiple-comparisons test (Supplementary Figure 2K for NLRP3, IL-1β, C-Caspase-1). All p values are shown in the figure, Source data are provided as a Source Data file.

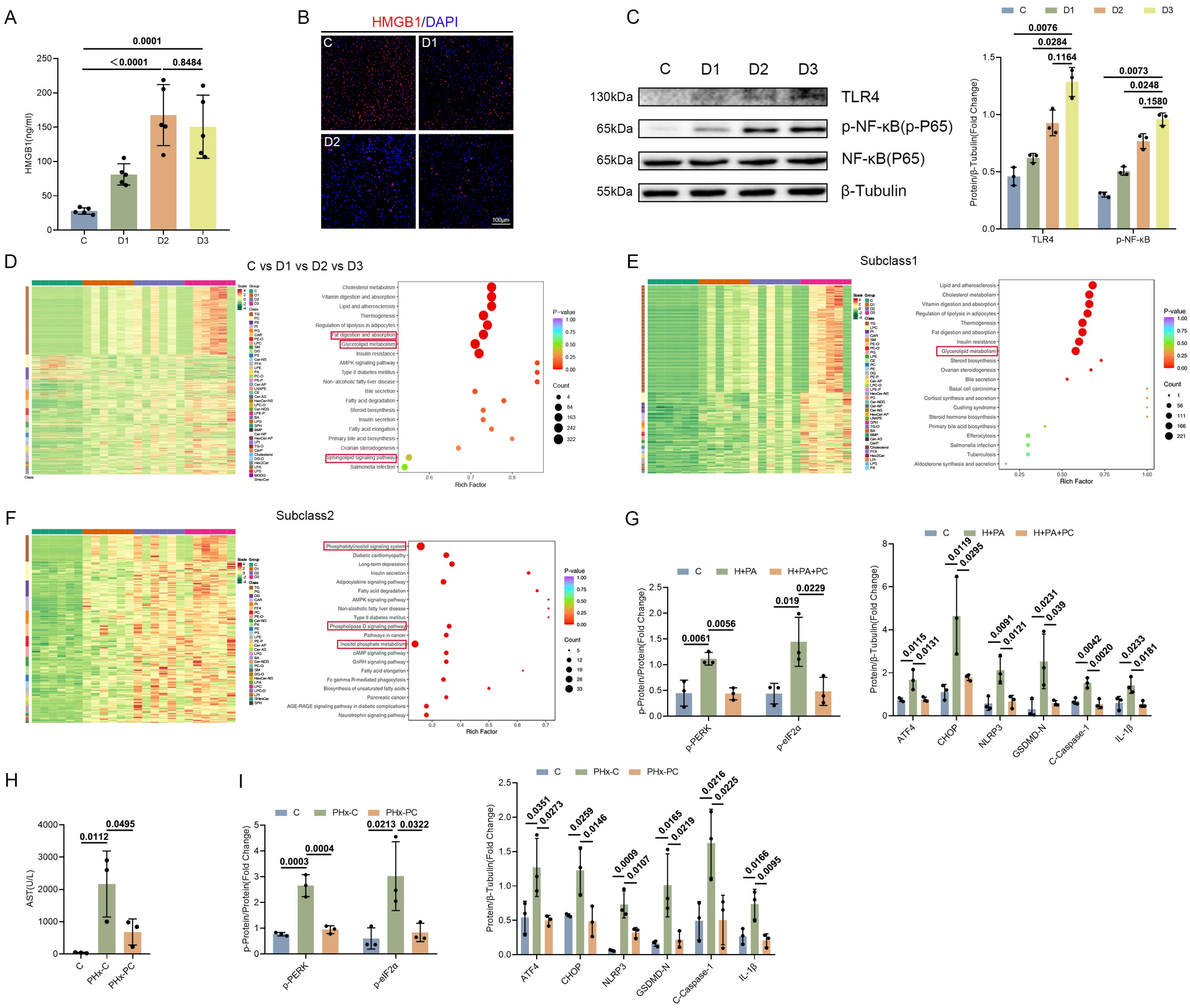

Supplementary Figure 3. Activation of TLR4/NF-κB signaling pathways and enrichment analysis of lipidomics-related signaling pathways during the acute phase following 70% PHx .

A: Serum HMGB1 concentrations at the indicated time points (C, D1, D2, and D3) following 70% PHx, as determined by ELISA (n=5 per group). B: Representative immunofluorescence images of HMGB1 (red) and nuclei (DAPI, blue) in liver tissues (Scale bar=100 μm, n=3 independent experiments). C: Representative western blot images (left panel) and corresponding quantitative analysis (right panel) of TLR4 and p-NF-κB (n=3 independent experiments). D: Heatmap clustering of differential lipids and KEGG pathway enrichment analysis for each group (n=6 per group). E: Expression heatmap and KEGG enrichment analysis of differential lipids in Subclass1.

F: Expression heatmap and KEGG enrichment analysis of differential lipids in Subclass2. G: Quantitative statistical analysis of protein expression levels corresponding to Figure 3H (n=3 independent experiments). H: Comparison of serum AST levels in each group (n=3 per group). I: Quantitative statistical analysis of protein expression levels corresponding to Figure 3P (n=3 per group). Abbreviations: AST, Aspartate aminotransferase; KEGG, Kyoto Encyclopedia of Genes and Genomes. Data are expressed as mean ± SD, statistical significance was analyzed using one-way ANOVA followed by Dunnett's multiple comparisons test. All p values are shown in the figure, Source data are provided as a Source Data file.

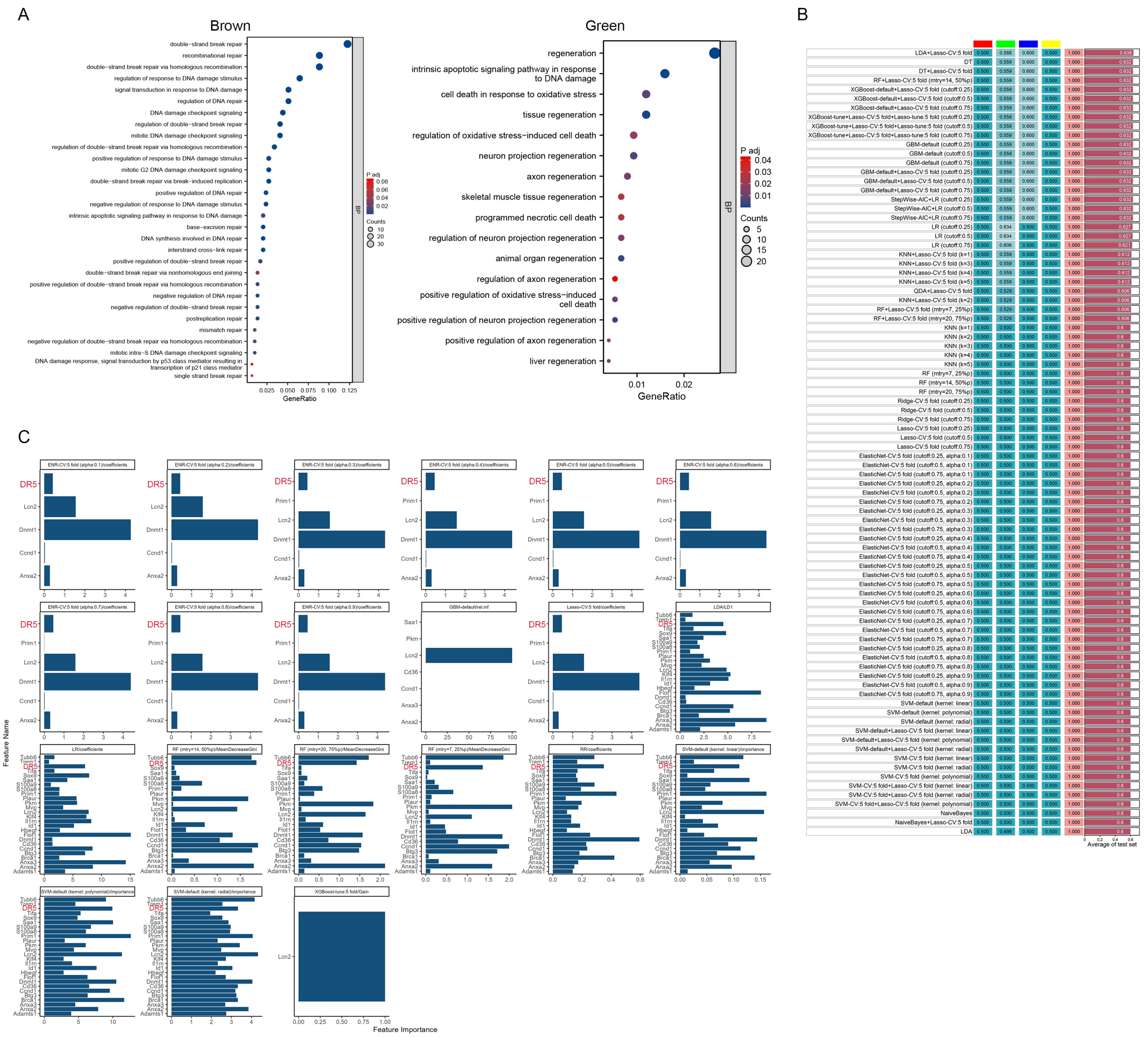

Supplementary Figure 4. Machine Learning-Derived Feature Importance Analysis of Pyroptosis-Related Genes After 70% PHx.

A: Functional enrichment analysis of the Brown (left panel) and Green (right panel) modules. B: Heatmap of model performance evaluation for candidate gene screening using multiple machine learning algorithms. C: Ranking of feature importance calculated by various machine learning algorithms, displaying the importance scores of genes such as DR5 across different models.

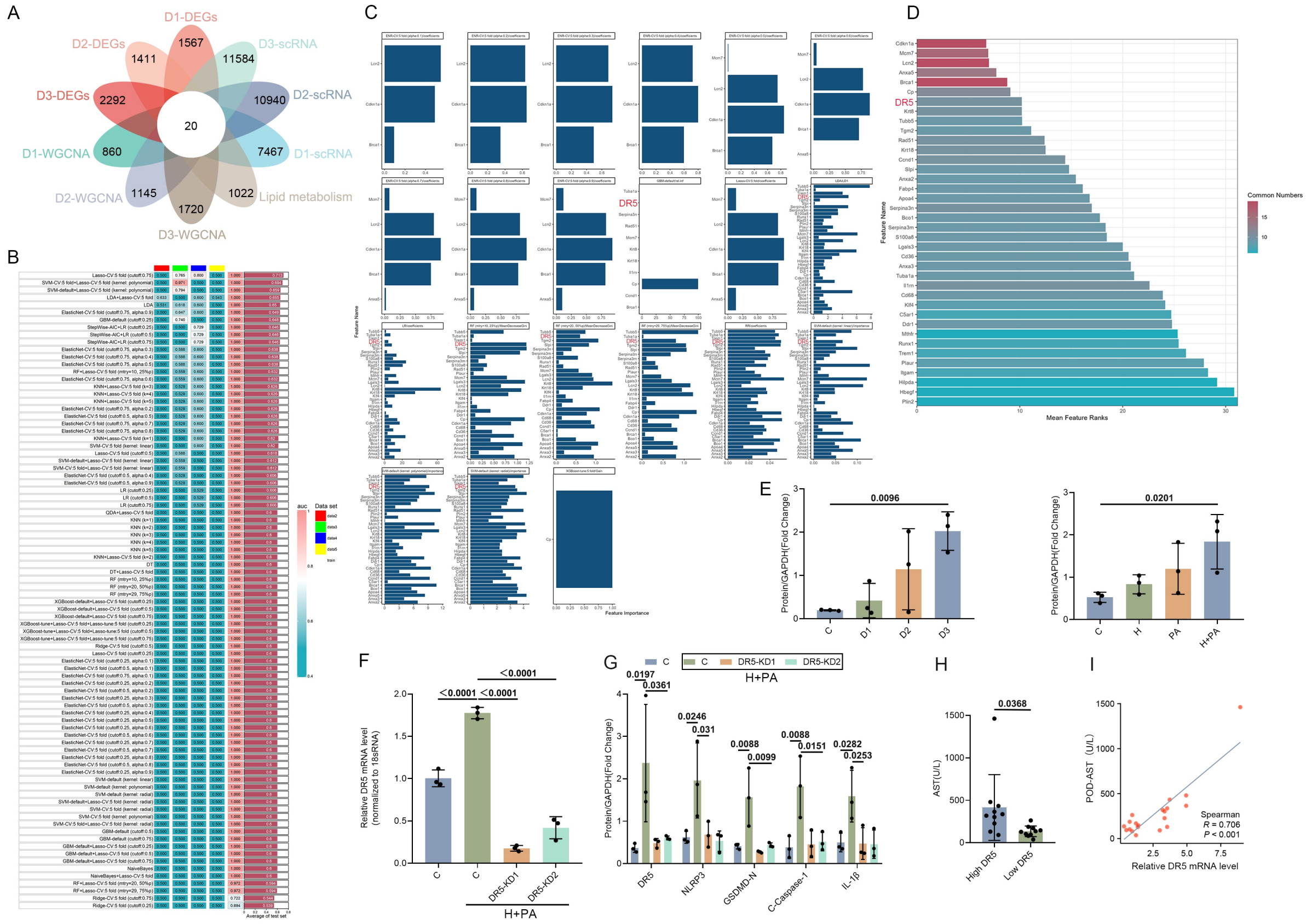

Supplementary Figure 5. Screening and validation of the key pyroptosis-related feature gene (DR5) using machine learning algorithms.

A: Venn diagram showing the screening of key genes from the intersection of RNA-seq DEGs (n=36 samples from 18 mice), scRNA-seq DEGs (CNP0002310), WGCNA gene modules (n=36 samples from 18 mice), and known lipid metabolism-related gene sets following 70% PHx. B: Heatmap of model performance evaluation for candidate gene screening using integrated machine learning algorithms. C: Feature importance ranking calculated by various machine learning algorithms. D: Average ranking of feature genes derived from multiple algorithms. E: Quantitative statistical analysis of protein expression levels corresponding to Figure 4H (n=3 independent experiments). F: RT-qPCR validation of DR5 expression levels following DR5 knockdown (n=3 independent experiments). G: Quantitative statistical analysis of protein expression levels corresponding to Figure 4I (n=3 independent

experiments). H: Comparison of postoperative serum AST levels between low and high DR5 expression groups classified based on DR5 levels after ALPPS-S2 (n=10 per group). I: Scatter plot of Spearman correlation analysis between relative DR5 mRNA expression in ALPPS-S2 liver tissue and peak postoperative serum AST levels (n=20 patients). Abbreviations: AST, Aspartate aminotransferase; RNA-seq, RNA sequencing; DEGs, Differentially expressed genes; scRNA-seq, Single-cell RNA sequencing; WGCNA, Weighted gene co-expression network analysis; DR5, Death receptor 5; ALPPS-S2, Stage 2 of associating liver partition and portal vein ligation for staged hepatectomy; POD, Postoperative day. Data are expressed as mean ± SD. Statistical significance was analyzed using one-way ANOVA followed by Dunnett's multiple comparisons test (Supplementary Figure 5E-G). Statistical significance was analyzed using a two-tailed unpaired Student's t-test (Supplementary Figure 5H). Correlation between relative DR5 mRNA levels and POD-ALT was analyzed using Spearman's rank correlation test (Supplementary Figure 5I). All p values are shown in the figure, Source data are provided as a Source Data file.

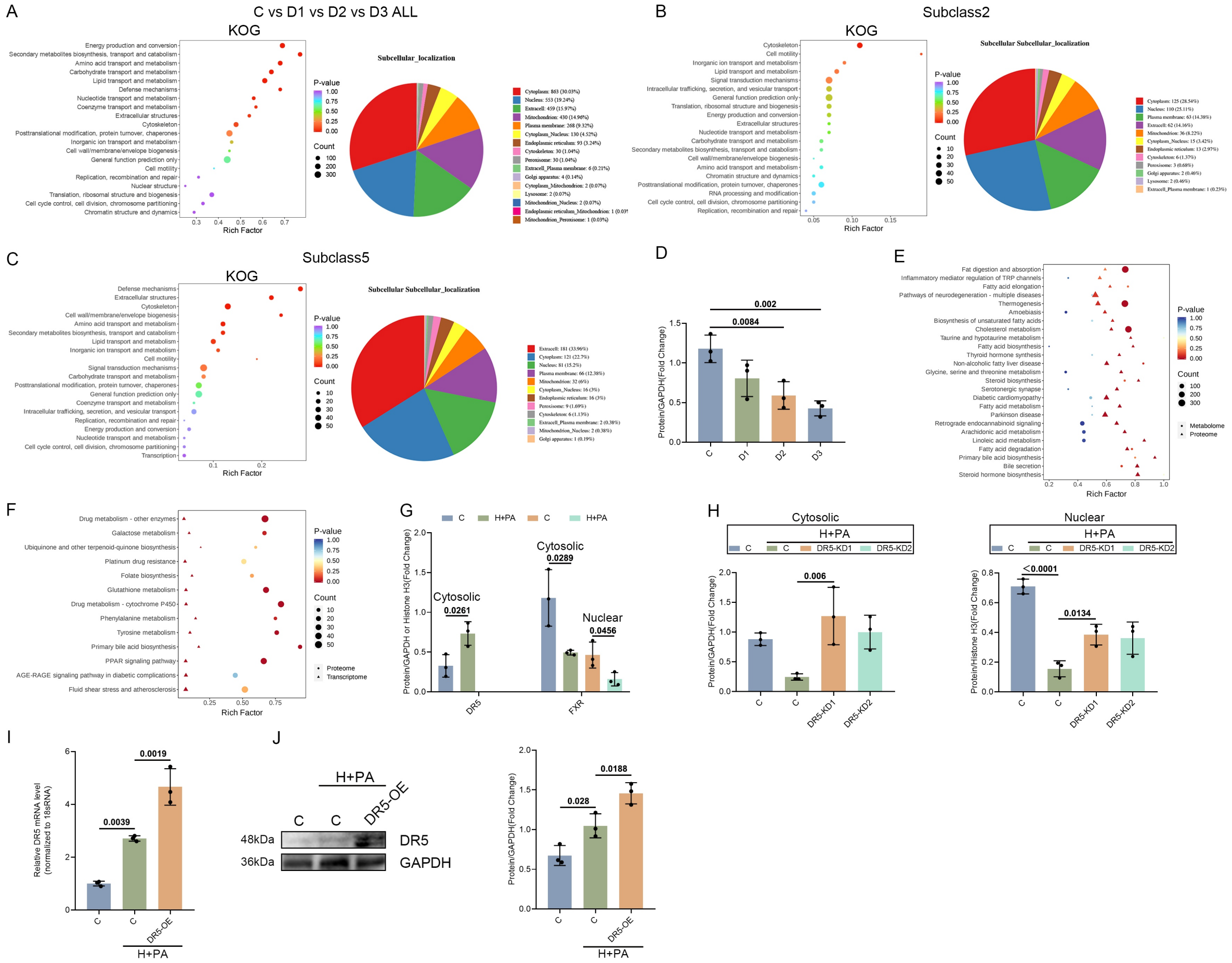

Supplementary Figure 6. Proteomic Functional Enrichment Analysis and Multi-Omics Screening.

A: KOG functional classification and subcellular localization analysis of differentially expressed proteins (n=6 per group). B: Functional annotation and localization analysis of Subclass2 proteins. C: Functional annotation and localization analysis of Subclass5 proteins. D: Quantitative statistical analysis of protein expression levels corresponding to Figure 5F (n=3 independent experiments). E: KEGG pathway enrichment analysis of the joint proteomics and metabolomics analysis. F: KEGG pathway enrichment analysis of the joint proteomics and transcriptomics analysis. G: Quantitative statistical analysis of protein expression levels corresponding to Figure 5I (n=3 independent experiments). H: Quantitative statistical analysis of protein

expression levels corresponding to Figure 5K (n=3 independent experiments). I: RT-qPCR validation of DR5 mRNA expression levels following DR5 overexpression (n=3 independent experiments). J: Representative western blot images of DR5 protein expression levels (left panel) and corresponding quantification (right panel) following DR5 overexpression in the cell model (n=3 independent experiments). Abbreviations: KEGG, Kyoto Encyclopedia of Genes and Genomes; KOG, euKaryotic Orthologous Groups. Data are expressed as mean ± SD. Statistical significance was analyzed using one-way ANOVA followed by Dunnett's multiple comparisons test (Supplementary Figure 6D and 6H-J). Statistical significance was determined by Student's two-tailed t-test (Supplementary Figure 6G). All p values are shown in the figure, Source data are provided as a Source Data file.

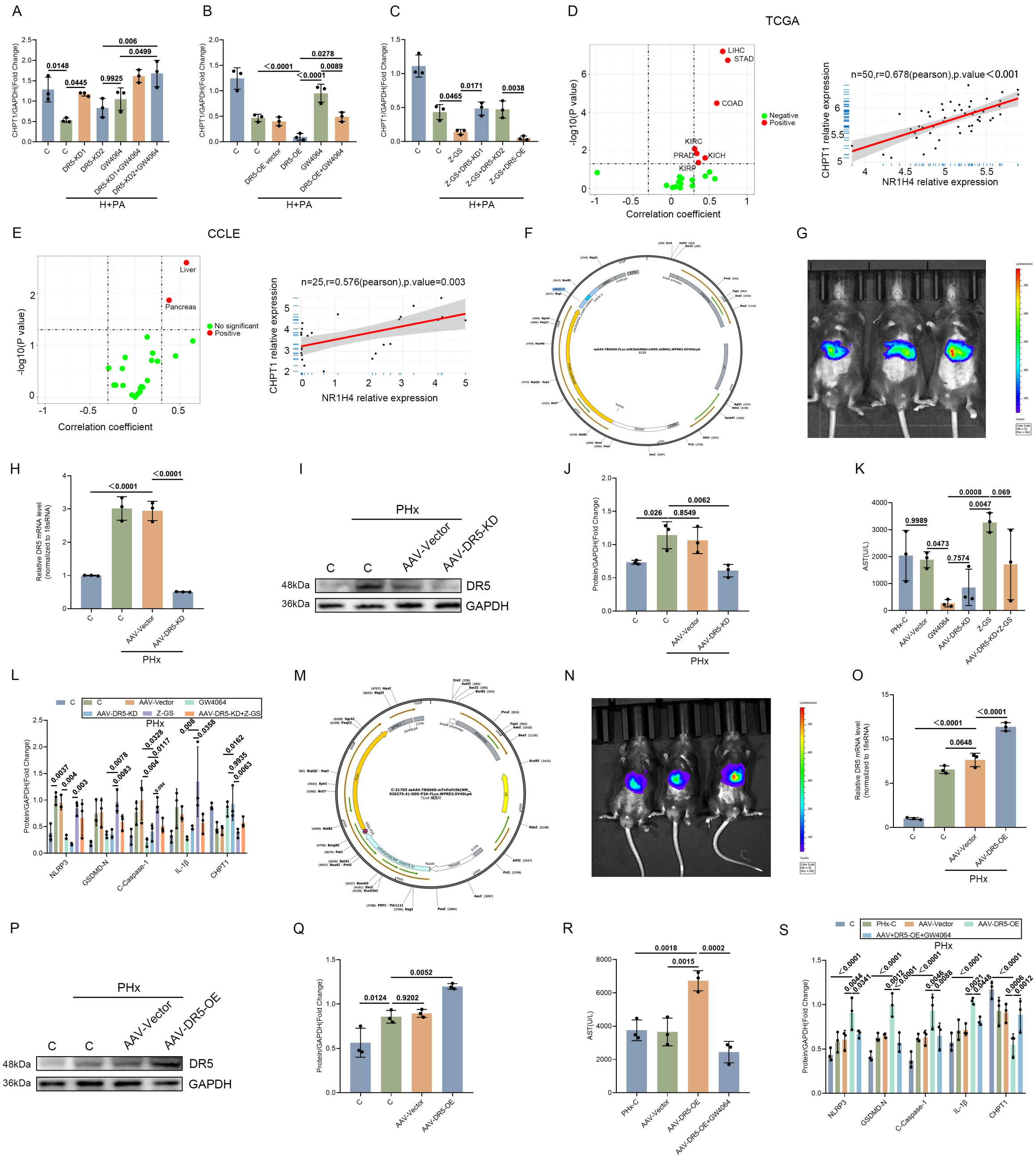

Supplementary Figure 7. Validation of the DR5–FXR–CHPT1 Axis in Mediating Pyroptosis and Liver Injury.

A: Quantitative statistical analysis of protein expression levels corresponding to Figure 6A (n=3 independent experiments). B: Quantitative statistical analysis of protein expression levels corresponding to Figure 6B (n=3 independent experiments). C: Quantitative statistical analysis of protein expression levels corresponding to Figure 6C (n=3 independent experiments). D: Correlation between CHPT1 and

NR1H4 (FXR) expression in the liver from the TCGA database. E: Correlation between CHPT1 and NR1H4 (FXR) expression in liver cell lines from the CCLE database. F: Map of the AAV-DR5-KD plasmid construction. G: In vivo imaging showing specific targeted infection and expression in the liver region following AAV-DR5-KD virus injection (n=3 per group). H: RT-qPCR validation of DR5 mRNA expression levels following AAV-DR5-KD administration (n=3 independent experiments). I: Representative western blot images of DR5 protein expression levels following AAV-DR5-KD administration (n=3 independent experiments). J: Quantitative statistical analysis of protein expression levels corresponding to Supplementary Figure 7I (n=3 independent experiments). K: Serum AST levels in each group (n=3 per group). L: Quantitative statistical analysis of protein expression levels corresponding to Figure 6M (n=3 per group). M: Map of the AAV-DR5-OE plasmid construction. N: In vivo imaging showing specific targeted infection and expression in the liver region following AAV-DR5-OE virus injection (n=3 per group). O: RT-qPCR validation of DR5 mRNA expression levels following AAV-DR5-OE administration (n=3 independent experiments). P: Representative western blot images of DR5 protein expression levels following AAV-DR5-OE administration (n=3 independent experiments). Q: Quantitative statistical analysis of protein expression levels corresponding to Supplementary Figure 7P (n=3 independent experiments). R: Serum AST levels in each group (n=3 per group). S: Quantitative statistical analysis of protein expression levels corresponding to Figure 6R (n=3 per group). Abbreviations: CHPT1, Choline phosphotransferase 1; NR1H4, Nuclear receptor

subfamily 1 group H member 4 (also known as FXR, farnesoid X receptor); TCGA, The Cancer Genome Atlas; CCLE, Cancer Cell Line Encyclopedia; AAV-DR5, Adeno-associated virus vector encoding/knock down death receptor 5; AST, Aspartate aminotransferase. Data are expressed as mean ± SD. Statistical significance was analyzed using one-way ANOVA followed by Tukey's multiple-comparisons test (Supplementary Figure 7A-C, L). Statistical significance was analyzed using one-way ANOVA followed by Dunnett's multiple-comparisons test (Supplementary Figure 7H, J, L, O, Q-S). All p values are shown in the figure, Source data are provided as a Source Data file.

Supplementary table 1 (Key resources table)

1.1 Antibodies

Name	Supplier	Cat no.
NLRP3	Abcam	ab263899
Gasdermin D	CST	39754
IL-1β	Abcam	ab283818
Cleaved-Caspase-1	CST	89332, 4199
PERK	CST	3192
P-PERK	ABclonal	AP1501
eIF2α	CST	5324
P-eIF2α	ABclonal	AP0692
ATF4	Affinity	DF6008
CHOP	Affinity	AF6277
DR5	Abcam	ab8416
FXR/NR1H4	CST	72105
CHPT1	ABclonal	A16137
β-Tubulin	CST	2146
Histone H3	CST	4499
GAPDH	CST	2118
Alexa Fluor 488-conjugated goat anti-mouse IgG	Invitrogen	2714439
Alexa Fluor 546-conjugated goat anti-rabbit IgG	Invitrogen	2570547
CD31	Abcam	ab281583
HMGB1	Abcam	ab79823
Caspase-1	CST	83383
HNF4α	Abcam	ab201460
TLR4	CST	14358
GSDMD-N	ABclonal	A24059
P-NF-κB	CST	3033
NF-κB	CST	6956
CD11b	Abcam	ab133357

1.2 Sequence-based reagents

Name	Sequence	Supplier
M-*18S*-F1	GGTGGAGTCATACGCATTCTGG	Tsingke
M-*18S*-R1	ACAGCATTCCCAGTGCCTTGGA	Tsingke
H-*18S*-F1	ACCCGTTGAACCCCATTCGTGA	Tsingke
H-*18S*-R1	GCCTCACTAAACCATCCAATCGG	Tsingke
M-*DR5*-F1	TGTGTCGATGCAAACCAGGCAC	Tsingke

M-*DR5*-R1	GCCGTTTTGGAGACACACTTCC	Tsingke
H-*DR5*-F1	AGCACTCACTGGAATGACCTCC	Tsingke
H-*DR5*-R1	GTGCCTTCTTCGCACTGACACA	Tsingke
H-*PERK*-F1	GTCCCAAGGCTTTGGAATCTGTC	Tsingke
H-*PERK*-R1	CCTACCAAGACAGGAGTTCTGG	Tsingke
M-*Perk*-F1	CCGATGTCAGTGACAACAGCTG	Tsingke
M-*Perk*-R1	AAGACAACGCCAAAGCCACCAC	Tsingke
H-*ATF4*-F1	TTCTCCAGCGACAAGGCTAAGG	Tsingke
H-*ATF4*-R1	CTCCAACATCCAATCTGTCCCG	Tsingke
M-*Atf4*-F1	AACCTCATGGGTTCTCCAGCGA	Tsingke
M-*Atf4*-R1	CTCCAACATCCAATCTGTCCCG	Tsingke
H-*DDIT3*-F1	GGTATGAGGACCTGCAAGAGGT	Tsingke
H-*DDIT3*-R1	CTTGTGACCTCTGCTGGTTCTG	Tsingke
M-*Ddit3*-F1	GGAGGTCCTGTCCTCAGATGAA	Tsingke
M-Ddit3-R1	GCTCCTCTGTCAGCCAAGCTAG	Tsingke
CHPT1-ChIP-up	AGTTCGTGGACCC	Tsingke
CHPT1-ChIP-down	GGGTCCACGAACT	Tsingke

1.3 Other (e.g. drugs etc.)

**Drugs**	**Supplier**	**Cat no.**
Mouse interleukin 18 (IL-18) enzyme-linked immunosorbent assay kit	Elabscience	E-EL-M0730
Mouse interleukin 1β (IL-1β) enzyme-linked immunosorbent assay kit	Elabscience	E-EL-M0037
Isoflurane	RWD	R510-22
Dako REAL™ EnVision™ Detection System	Dako	K5007
ChIP Assay Kit	Merck millipore	17-10086
ECL Western Blotting kit	NCM	P10300
TRIzol	ThermoFisher	15596026
Oil Red O staining	Beyotime	C0158S
Rat tail collagen type I	Solarbio	C8062
glucose-free DMEM	Gibco	11966025
RPMI 1640	Gibco	C11875500BT
DMEM	Gibco	C11995500BT
FBS	Gibco	10099141C
Polyene phosphatidylcholine	Weishi	hbws-958
WY-14643	MCE	HY-16995

Etomoxir (ETO)	Selleck	S8244
GW4064	MCE	HY-50108
L-α-phosphatidylcholine	Sigma-Aldrich	P5394
(Z)-Guggulsterone (Z-GS)	MCE	HY-110066
Nuclear and Cytoplasmic Protein Extraction Kit	Beyotime	P0028
Protein A/G Magnetic Beads	Elabscience	EA-IP-007M
CCK-8	KeyGEN	KGA317
HMGB1 ELISA Kit	ABclonal	RK06737

1.4 Data and biological samples

**Data**	**Biological samples**
RNA-seq (training set)	n=36
GSE151309	n=5
GSE20426	n=35
GSE181476	n=14
CNP0002310	n=30
90% PHx (scRNA-seq)	n=3
Proteomics	n=24
Lipidomics	n=24
