# Supplementary material for: Multi-omics reveals the DR5-FXR-CHPT1 axis governs hepatocyte pyroptosis via glycerophospholipid remodeling after major partial hepatectomy in male mice
Source: Nat Commun. 2026 Oct 7;17:11423. doi: 10.1038/s41467-026-78113-8 (PMC13737082; doi:10.1038/s41467-026-78113-8)
Supplement: Supplementary file 2 [file 41467_2026_78113_MOESM2_ESM.pdf]

Title: Supplementary Data 1

Description: Detailed proteomics identification, quantification, and functional annotation results.

Peptides_Summary: Details the information of all identified peptides, including peptide sequences, precursor charge, missed cleavages, mapped protein groups, and the quantification values across all 24 samples (4 groups: C, D1, D2, D3, with 6 replicates per group).

Protein_Annot_Summary: Presents the protein-level quantification and comprehensive bioinformatics annotations. It includes protein accession numbers, gene names, peptide counts, relative abundance across all samples, and functional annotations such as Gene Ontology (Biological process, Cellular component, Molecular function), KOG, KEGG pathways, InterPro domains (IPRs), subcellular localization, and signal peptide (signalP) predictions.

Title: Supplementary Data 2

Description: Detailed lipidomics identification, quantification, and structural annotation results. This file provides comprehensive data on all identified lipid compounds. The spreadsheets include:

Lipid Identification & Properties (Sheet1): Detailed chemical and structural information for each lipid, including compound name, primary and secondary lipid categories (Class I and Class II), carbon chain length, chain unsaturation, chemical formula, molecular weight, precursor ion mass (Q1), and ionization mode.

Quantification & Pathway Mapping (Sheet2): The relative quantitative abundance of each lipid compound across all 24 samples (4 experimental groups: C, D1, D2, D3, with 6 biological replicates per group). Contains additional Kyoto Encyclopedia of Genes and Genomes (KEGG) pathway mapping (kegg_map) for the functionally annotated lipid metabolites.
