# Supplementary material for: Multi-omics reveals the DR5-FXR-CHPT1 axis governs hepatocyte pyroptosis via glycerophospholipid remodeling after major partial hepatectomy in male mice
Source: Nat Commun. 2026 Oct 7;17:11423. doi: 10.1038/s41467-026-78113-8 (PMC13737082; doi:10.1038/s41467-026-78113-8)
Supplement: Supplementary file 3 [file 41467_2026_78113_MOESM5_ESM.pdf]

nature portfolio

Corresponding author(s): Linsen Ye, Zenan Yuan, Changchang Jia, Hua Li

Last updated by author(s): Aug 18, 2026

Reporting Summary

Nature Portfolio wishes to improve the reproducibility of the work that we publish. This form provides structure for consistency and transparency in reporting. For further information on Nature Portfolio policies, see our Editorial Policies and the Editorial Policy Checklist.

Statistics

For all statistical analyses, confirm that the following items are present in the figure legend, table legend, main text, or Methods section.

n/a	Confirmed	
☐	☒	The exact sample size (*n*) for each experimental group/condition, given as a discrete number and unit of measurement
☐	☒	A statement on whether measurements were taken from distinct samples or whether the same sample was measured repeatedly
☐	☒	The statistical test(s) used AND whether they are one- or two-sided<br>*Only common tests should be described solely by name; describe more complex techniques in the Methods section.*
☒	☐	A description of all covariates tested
☐	☒	A description of any assumptions or corrections, such as tests of normality and adjustment for multiple comparisons
☐	☒	A full description of the statistical parameters including central tendency (e.g. means) or other basic estimates (e.g. regression coefficient) AND variation (e.g. standard deviation) or associated estimates of uncertainty (e.g. confidence intervals)
☐	☒	For null hypothesis testing, the test statistic (e.g. F, t, r) with confidence intervals, effect sizes, degrees of freedom and P value noted<br>*Give P values as exact values whenever suitable.*
☒	☐	For Bayesian analysis, information on the choice of priors and Markov chain Monte Carlo settings
☒	☐	For hierarchical and complex designs, identification of the appropriate level for tests and full reporting of outcomes
☐	☒	Estimates of effect sizes (e.g. Cohen's *d*, Pearson's *r*), indicating how they were calculated

Our web collection on statistics for biologists contains articles on many of the points above.

Software and code

Policy information about availability of computer code

Data collection	Immunofluorescence: confocal microscope (Zeiss LSM 880) (The fluorescence emission spectrum covers the range of 400–750 nm);<br>Transmission electron microscopy: transmission electron microscope (HITACHI);<br>Scanning Electron Microscopy: scanning electron microscopy(HITACHI);<br>Protein concentration and ELISA analysis: Multimode Microplate Reader (Bio-Tek);<br>Real-time quantitative PCR: LightCycler 480 (RPCHE);<br>Western blot assay: Tanon-5200CE.
Data analysis	All statistical analyses were performed using GraphPad Prism (version 8.0.1);<br>single-cell-resolution inference of pyroptosis activity: UCell R package (version 2.6.2);<br>RNA-seq analysis: DEGs were determined by DESeq2 (v.1.30.1) and Functional annotation was performed with Metascape (v.3.5);<br>K-Means clustering analysis: R stats package (version 4.1.2);<br>Immunofluorescence and hematoxylin and eosin (H&E) analysis: Image J software.<br>All custom R scripts utilized for WGCNA, machine learning, multi-omics integration, and single-cell transcriptomic analyses are accessible via GitHub (https://github.com/tys980915/code.git), accompanied by a README guide to facilitate the reproduction of the analytical pipeline.

For manuscripts utilizing custom algorithms or software that are central to the research but not yet described in published literature, software must be made available to editors and reviewers. We strongly encourage code deposition in a community repository (e.g. GitHub). See the Nature Portfolio guidelines for submitting code & software for further information.

Data

Policy information about availability of data

All manuscripts must include a data availability statement. This statement should provide the following information, where applicable:

- Accession codes, unique identifiers, or web links for publicly available datasets
- A description of any restrictions on data availability
- For clinical datasets or third party data, please ensure that the statement adheres to our policy

Data Availability
All RNA-seq and ScRNA-seq data have been deposited in the Sequence Read Archive (SRA) under accession number PRJNA1513559 and PRJNA1490015 (https://www.ncbi.nlm.nih.gov/sra/?term=PRJNA1513559; https://www.ncbi.nlm.nih.gov/sra/?term=PRJNA1490015). Proteomics data have been deposited to the ProteomeXchange Consortium via the iProX partner repository59,60 with the dataset identifier PXD080626 (http://proteomecentral.proteomexchange.org/cgi/GetDataset?ID=PXD080626; https://www.iprox.cn/page/project.html?id=IPX0018189000). Lipidomics generated in this study have been deposited to MetaboLights repository61 with the study identifier MTBLS14980 (https://www.ebi.ac.uk/metabolights/MTBLS14980). All data supporting the findings of this study are included in the main text, the Supplementary Information, or the Source Data file. Source data are provided with this paper.
Code Availability
All R scripts utilized for WGCNA, machine learning, multi-omics integration, and single-cell transcriptomic analyses are publicly accessible via GitHub (https://github.com/tys980915/code.git), accompanied by a README guide to facilitate the reproduction of the analytical pipeline. To ensure long-term preservation and citeability, this repository has also been archived on the Zenodo platform (https://doi.org/10.5281/zenodo.21936844).

Research involving human participants, their data, or biological material

Policy information about studies with human participants or human data. See also policy information about sex, gender (identity/presentation), and sexual orientation and race, ethnicity and racism.

Reporting on sex and gender	Our study did not identify findings that apply exclusively to one sex or gender. To investigate the injury dynamics of the remnant liver tissue following PHx, we retrospectively collected data from 371 cases, including anatomical left hemihepatectomy (n=174 patients, 112 male and 62 female patients), anatomical right hemihepatectomy (n=145 patients,106 male and 39 female patients), and ALPPS (n=52 patients,50 male and 2 female patients). Gender was not considered in this study. Written informed consent was obtained from all participants.
Reporting on race, ethnicity, or other socially relevant groupings	No information/data about race, ethnicity or other socially relevant groupings were collected.
Population characteristics	The study population comprised 174 patients who underwent left hemihepatectomy, 145 patients who underwent right hemihepatectomy and 52 patients who underwent ALPPS for hepatocellular carcinoma (HCC) at the Department of Hepatobiliary Surgery of the Third Affiliated Hospital of Sun Yat-sen University between January 1, 2002 and December 31, 2024. Tissue specimens were obtained from the Department of Pathology or the Liver Disease Laboratory Specimen Bank at the same institution. Inclusion criteria were: (1) patients with HCC who underwent left or right hemihepatectomy after exclusion of surgical contraindications; (2) age between 18 and 70 years, regardless of sex; and (3) ability to understand the study and provide written informed consent. Exclusion criteria were: (1) blood transfusion during or within 2 weeks prior to surgery; (2) severe psychiatric illness or substance abuse; (3) refusal to participate; and (4) pregnancy or lactation.
Recruitment	No donors were recruited. This study exclusively utilizes pre-existing clinical records and archived specimens, with explicit exclusion of any materials previously declined by patients/subjects for research use. All participants were informed about the study and provided written informed consent before inclusion. There are no potential self-selection bias or other biases present in our study.
Ethics oversight	The study was conducted in accordance with the Declaration of Helsinki and approved by the Clinical Research Ethics Committee of the Third Affiliated Hospital of Sun Yat-sen University (?2025-316-1) (Guangzhou, China).

Note that full information on the approval of the study protocol must also be provided in the manuscript.

Field-specific reporting

Please select the one below that is the best fit for your research. If you are not sure, read the appropriate sections before making your selection.

☒ Life sciences ☐ Behavioural & social sciences ☐ Ecological, evolutionary & environmental sciences

For a reference copy of the document with all sections, see nature.com/documents/nr-reporting-summary-flat.pdf

Life sciences study design

All studies must disclose on these points even when the disclosure is negative.

Sample size	No statistical method was used to predetermine sample size, While a formal power analysis was not conducted, the sample size was determined based on previous studies and preliminary experiments in this research area. Sample sizes in vivo and vitro were determined by

	three or more samples for comparisons between one or multiple groups, followed by the statistical test.
Data exclusions	No pre-established data exclusion method was used.
Replication	The experimental findings can be reliably reproduced. Each experimental condition was independently repeated no fewer than three times (typically exceeding five replicates in most cases), and specific repetition numbers are provided in figure legends. The "replicates" for in vitro experiments refer to independent experiments, andanimal studies now report the number of animals per group.
Randomization	Patient-derived samples were randomly acquired by technical personnel blinded to study objectives, and experimental animals were systematically distributed to cages by animal center operators unaffiliated with the investigation. This randomization approach prevented any selection bias in group assignments.
Blinding	Investigators were blinded to group allocation during data collection and analysis.

Reporting for specific materials, systems and methods

We require information from authors about some types of materials, experimental systems and methods used in many studies. Here, indicate whether each material, system or method listed is relevant to your study. If you are not sure if a list item applies to your research, read the appropriate section before selecting a response.

Materials & experimental systems

n/a	Involved in the study
☐	☒ Antibodies
☐	☒ Eukaryotic cell lines
☒	☐ Palaeontology and archaeology
☐	☒ Animals and other organisms
☐	☒ Clinical data
☒	☐ Dual use research of concern
☒	☐ Plants

Methods

n/a	Involved in the study
☒	☐ ChIP-seq
☒	☐ Flow cytometry
☒	☐ MRI-based neuroimaging

Antibodies

Antibodies used	Western blot assay:<br>NLRP3 (1:1000; ab263899, Abcam), Gasdermin D (1:1000; #39754; CST), IL-1β (1:1000; ab283818; Abcam), Cleaved-Caspase-1 (1:1000; #89332, #4199; CST), PERK (1:1000; #3192; CST), P-PERK (1:1000; AP1501; ABclonal), eIF2α (1:1000; #5324; CST), P-eIF2α (1:1000; AP0692; ABclonal), ATF4 (1:1000; DF6008; Affinity), CHOP (1:1000; AF6277; Affinity), DR5 (1:1000; ab8416; Abcam), FXR/NR1H4 (1:1000; #72105; CST), CHPT1 (1:1000; A16137; ABclonal), β-Tubulin (1:1000; #2146; CST), Histone3 (#1:1000; 4499, CST), GAPDH (1:1000; #2118, CST), Caspase1 (#1:1000; 83383; CST), TLR4 (#1:1000; 14358, CST), GSDMD-N (1:1000; A24059; ABclonal), P-NF-κB (1:1000; #3033; CST).<br><br>Immunofluorescence:<br>DR5 (1:800; ab8416; Abcam), FXR/NR1H4(1:800; #72105; CST), CHPT1(1:800; A16137; ABclonal), HMGB1(1:800; ab79823; Abcam), HNF4α(1:800; ab201460; Abcam), GSDMD-N(1:800; A24059; ABclonal), CD31(1:800; ab281583; Abcam), CD11b(1:800; ab133357; Abcam). Alexa Fluor 488-conjugated goat anti-mouse IgG (1:800; #2714439; Invitrogen), Alexa Fluor 546-conjugated goat anti-rabbit IgG (1:800; #2570547; Invitrogen).<br><br>Co-immunoprecipitation assay: Specific antibody for CoIP: DR5 (2 μg; ab8416; Abcam), CHPT1 (2 μg; A16137; ABclonal).<br><br>Chromatin immunoprecipitation assay: Specific antibody for ChIP: FXR/NR1H4(2μg; #72105; CST).
Validation	All purchased antibodies have been validated by the manufacturers for their corresponding applications. We have further validated the antibodies used for Western blotting based on the molecular weight of the target proteins.

Eukaryotic cell lines

Policy information about cell lines and Sex and Gender in Research

Cell line source(s)	The THLE -2 cell line were obtained from ATCC.
Authentication	Cell lines were not authenticated.
Mycoplasma contamination	The cell lines were regularly tested negative for mycoplasma.
Commonly misidentified lines<br>(See ICLAC register)	No misidentified lines were used.

Animals and other research organisms

Policy information about studies involving animals; ARRIVE guidelines recommended for reporting animal research, and Sex and Gender in Research

Laboratory animals	Wild-type C57BL/6J mice aged 6-8 weeks were obtained from Jiangsu Gempharmatech Biological Science and Technology Ltd (Nanjing, Jiangsu, China) and bred at the Sun Yat-sen University Laboratory Animals Center. All mice were maintained in a special pathogen-free (SPF) environment with a temperature of 20°C to 25°C, relative humidity of about 50%, and a 12-hour light-dark cycle.
Wild animals	No wild animals were used.
Reporting on sex	Sex was not considered in study design, and no sex-based analyses was performed.
Field-collected samples	This study did not involve field-collected samples.
Ethics oversight	All animal procedures were approved by the Institutional Animal Care and Use Committee (IACUC), Sun Yat-Sen University (SYSU-IACUC-2025-001978), and conducted in compliance with National Institutes of Health's Guide for the Care and Use of Laboratory Animals (#JENNIO-IACUC-2024-A062).

Note that full information on the approval of the study protocol must also be provided in the manuscript.

Clinical data

Policy information about clinical studies
All manuscripts should comply with the ICMJE guidelines for publication of clinical research and a completed CONSORT checklist must be included with all submissions.

Clinical trial registration	This study was registered in the Medical Research Registration and Filing System, and was approved by the Clinical Research Ethics Committee of the Third Affiliated Hospital of Sun Yat-sen University (?2025-316-1).
Study protocol	The protocol has been approved by the Ethics Committee of the Third Affiliated Hospital of Sun Yat-sen University. retrospective cohort study aims to investigate the factors influencing hepatocyte injury following liver resection and their underlying molecular mechanisms. Patients with hepatocellular carcinoma who underwent left hemihepatectomy or right hemihepatectomy at the Department of Hepatobiliary Surgery, Third Affiliated Hospital of Sun Yat-sen University, between January 1, 2002 and December 31, 2024, were enrolled. Inclusion criteria were: (1) histologically confirmed HCC with left or right hemihepatectomy after exclusion of surgical contraindications; (2) age 18–70 years, regardless of sex; and (3) provision of written informed consent for medical data and residual specimen use. Exclusion criteria were: (1) blood transfusion during or within 2 weeks prior to surgery; (2) severe psychiatric illness or substance abuse; (3) refusal to provide informed consent; and (4) pregnancy or lactation. Tissue specimens were obtained from the Department of Pathology or the Liver Disease Laboratory Specimen Bank. Protein and RNA expression in resected liver specimens were assessed by immunohistochemistry, Western blot, or PCR, and patients were stratified into high- and low-expression groups. Clinical data, including demographics, tumor stage, pathological grade, biochemical indices (AFP, ALB, TBIL), hematological parameters, and imaging findings (CT, MRI, PET-CT).The primary endpoint was postoperative liver function changes within 3 days after hepatectomy and alterations in gene expression in biospecimens. Parametric tests (independent samples t-test, one-way and two-way ANOVA) or non-parametric tests (Mann–Whitney U, Kolmogorov–Smirnov, Kruskal–Wallis, and Wilcoxon tests) were applied as appropriate, with statistical significance set at $p < 0.05$. All analyses were performed using SPSS and GraphPad Prism software.teria, and statistical analysis plan, are summarized in this manuscript.
Data collection	This study retrospectively collected clinical data and postoperative liver function parameters, including alanine aminotransferase [ALT], aspartate aminotransferase [AST], albumin [ALB], prothrombin time [PT], and international normalized ratio [INR], from the electronic medical record system of the Third Affiliated Hospital of Sun Yat-sen University between January 1, 2002 and December 31, 2024. Resected liver tissue specimens were obtained from the Department of Pathology or the Liver Disease Laboratory Specimen Bank. No follow-up was required. All residual specimens were disposed of as biomedical waste in accordance with institutional guidelines.
Outcomes	(1)Alanine Aminotransferase (ALT) levels peaked within postoperative day 3 (D3) following left hemihepatectomy, right hemihepatectomy, and ALPPS Step-1.<br>(2)Retrospective analysis revealed that patients with high DR5 expression<br>had significantly elevated ALT and AST levels following Step 2, and DR5 expression positively correlated with serum ALT and AST on D1.

Plants

Seed stocks	This study did not involve plants.
Novel plant genotypes	This study did not involve plants.
Authentication	This study did not involve plants.
