# Supplementary material for: Multi-omics reveals the DR5-FXR-CHPT1 axis governs hepatocyte pyroptosis via glycerophospholipid remodeling after major partial hepatectomy in male mice
Source: Nat Commun. 2026 Oct 7;17:11423. doi: 10.1038/s41467-026-78113-8 (PMC13737082; doi:10.1038/s41467-026-78113-8)
Supplement: Supplementary file 4 [file 41467_2026_78113_MOESM6_ESM.pdf]

nature portfolio

Peer Review File

Multi-omics reveals the DR5-FXR-CHPT1 axis governs hepatocyte pyroptosis via glycerophospholipid remodeling after major partial hepatectomy in male mice

Corresponding Author: Professor Hua Li

This file contains all reviewer reports in order by version, followed by all author rebuttals in order by version.

Version 0:

Reviewer comments:

Reviewer #1

(Remarks to the Author)
Comments to the Authors

This manuscript addresses the mechanism of liver injury after major partial hepatectomy (PHx) through an integrative multi-omics framework, combining human blood data with multiple mouse datasets, including single-cell transcriptomics, bulk RNA-seq, lipidomics, and proteomics. Overall, the study is ambitious, and the broad experimental design appears potentially valuable. The proposed DR5–FXR–CHPT1 axis is interesting and may be relevant to understanding hepatocyte pyroptosis and glycerophospholipid remodeling after PHx. However, several major issues currently prevent a proper assessment of the methodological rigor and robustness of the conclusions.

Major comments
1. Sample size and cohort information are insufficiently reported throughout the manuscript.
This is my main concern. It is currently very unclear how many biological samples were included in each analysis, and without this information it is not possible to evaluate the statistical robustness or reliability of the findings. For example, the number of samples used for WGCNA should be clearly stated, as this is particularly important for assessing the validity of module identification and correlation analyses. If the sample size is small, especially below the range typically considered suitable for WGCNA, the reliability of the network analysis becomes questionable. The authors should clearly report sample numbers, biological replicates, and the exact datasets used for every experiment and analysis.
2. The manuscript shifts between 70% PHx and 90% PHx in a way that makes the study design difficult to follow.
The logic for when 70% PHx versus 90% PHx is used is not sufficiently clear. At present, the results section appears to move between these two models without a clear framework, which makes it difficult to determine whether the conclusions are intended to apply to one condition, the other, or both. This also raises concern about selective presentation of results. The authors should either focus primarily on one PHx model for the main mechanistic conclusions or present the two conditions in parallel in a more systematic and consistent manner.
3. The machine learning analysis is inadequately described.
The manuscript refers to machine learning methods, but the modeling framework is not defined clearly enough for evaluation or reproduction. It is not clear what the objective of the model is, what the input features are, what the output variable is, or whether the model is performing classification or regression. If it is a classification model, the classes should be explicitly defined. In addition, the training, validation, and testing procedures, performance metrics, and methods for feature selection should all be described in detail. At present, the analysis is not interpretable, and reproducibility is further limited by the lack of code and data availability.
4. The novelty and significance of the phosphatidylcholine-related findings need to be clarified.
A substantial part of the manuscript focuses on the role of phosphatidylcholine (PC) in PHx-induced liver injury. However, the authors themselves note in the Introduction that this aspect has already been relatively well described in the literature. Therefore, it is unclear what the genuinely new conceptual advance is here. The authors should better distinguish their work from prior studies and explain more clearly what this study adds beyond the existing knowledge base.
5. Functional validation of DR5 appears incomplete.
The manuscript seems to rely primarily on DR5 silencing to support the proposed mechanism. While loss-of-function data are useful, complementary gain-of-function experiments would substantially strengthen the causal interpretation. For

example, DR5 overexpression in vivo or in vitro could help determine whether increased DR5 is sufficient to induce the expected phenotype. If the current dataset is sufficiently powered and the authors believe silencing alone is adequate, this needs to be justified more clearly. Again, this is difficult to assess without clear reporting of sample sizes.
Minor comments
6. Line 147:
The sentence beginning “While DR5 has frequently been targeted in therapies for solid tumors…” appears incomplete and should be revised for clarity.
7. Line 221:
The authors report Oil Red O staining confirming intrahepatic lipid accumulation following 70% PHx. Given the broader comparison between 70% and 90% PHx, it would be helpful to also show the corresponding staining results for 90% PHx.
8. Line 303:
The statement that “The Brown and Green modules showed high Pearson correlation coefficients (>0.7) with PHx, suggesting their importance” is not sufficiently justified. A strong correlation does not by itself establish biological importance. The authors should either rephrase this claim more cautiously or provide stronger reasoning and supporting evidence.
9. Pseudotime analysis is insufficiently described.
The manuscript refers to pseudotime analysis multiple times, but I could not find an adequate description of this analysis in the Methods. The authors should explain how pseudotime was inferred, which cells were included, what software/package was used, and how the resulting trajectories were interpreted.
10. All omics data and code for the reproduction of this study are missing, which is not acceptable.

(Remarks on code availability)
No code is provided.

Reviewer #2

(Remarks to the Author)
This study employs multi-omics integrative analysis and machine learning algorithms to propose a novel mechanism whereby the DR5-FXR-CHPT1 axis regulates hepatocyte pyroptosis following major partial hepatectomy. The study is highly innovative because previous studies never established the causal relationship between glycerophospholipid metabolism and pyroptosis. However, this study lacks several critical evidences and thus the conclusion is not solidly supported. The manuscript will be more unsuitable for publication in Nature Communications if these evidences are supplemented. My specific suggestions are as follows:
1. Figure 1D demonstrates upregulation of pyroptosis-related proteins; however, could this result potentially arise from pyroptosis occurring in cell types other than hepatocytes? Please clarify which cell type(s) primarily undergo pyroptosis following major partial hepatectomy.
2. Figure 1F presents transmission electron microscopy (TEM) observations of extensive membrane pore formation and organelle structural damage in hepatocytes. How do the authors demonstrate that the cells observed by electron microscopy are indeed hepatocytes?
3. The authors conducted DR5 overexpression and knockdown experiments in both cellular and animal models; however, they should assess the efficiency of DR5 overexpression or knockdown.
4. For Western blot of GSDMD-N and C-caspase-1, the full length protein should also be detected.
5. For Oil Red O staining, quantitation should also be shown.
6. According to a previous study (PMID: 34890842) and scRNA-seq data from human protein atlas website (https://www.proteinatlas.org/), the expression level of NLRP3 and caspase-1 in hepatocytes are extremely low in untreated state. The authors should explain why pyroptosis could occur in hepatocytes. Are there expressions induced by PHx? What is the possible mechanism?

(Remarks on code availability)

Reviewer #3

(Remarks to the Author)
The authors used Multi-omics approaches combined with machine learning method reveals the DR5-FXR-CHPT1 axis governs hepatocyte pyroptosis via glycerophospholipid remodeling after major partial hepatectomy. The data was comprehensive and the writing was largely well. I have the following minor concerns and comments mainly focus on proteomics and lipidomics analyses:
• The number of replicates should be stated clearly for proteomics and lipidomics analysis
• How much peptide was injected to mass spec for proteomics analysis? Will the proteomics data uploaded to Pride or other places?
• Lipidomis analysis: Since MRM method was used, what are the target lipids analyzed? Was the lipid quantification based on internal lipid standards or it is a relative quantification?
• How exactly Multi-omics integration (Metabolomics+Proteomics+Transcriptomics) was done?
Other minor points:
• Line 122: add a comma at the end
• Line 939-948 need to be re-edited
• Line 982: remove “with were measured”

• Line 987: change “Cell model” to “cell model”
• Keep consistent for “min” or “minutes” for the whole manuscript
• Line 1049: change “detection” to “measurement”
• Line 1052: add space before “Briefly”; change “tissues” to “tissue”
• Line 1124: change to “Proteomics analysis by LC-MS/MS”
• Line 1125: the sentence need to be re-edited
• Line 1138: remove “LC-MS/MS Analysis (Orbitrap Astral)”
• Line 1144: change “Mass spectrometry was performed” to “After LC separation, the peptides were analyzed”
• Line 1152: delete “Data Processing”
• Line 1158: change “Lipidomics Analysis” to “Lipidomics analysis by LC-MS/MS”
• Line 1159: remove “Lipid Extraction”
• Line 1165: remove “UPLC-MS/MS conditions”
• Line 1221: remove "Multiplex Immunofluorescence Staining"
• Line 1237: change “mm3” to “mm3”
• Line 1245: change “Electron Microscopy” to “electron microscopy”
• Line 1246: change “mm^2” to “mm3”
• Line 1247: add a space before “Samples”
• Line 1248: it is “PB” or “PBS”?
• Line 1254: add “ was used” after “ANOVA”
• Line 1256: add a space before “Statistical”
• Line 1258: change “performedusing” to “performed by”
• Fig 2L: is the unite for liver FFA “nmol”?
• Fig 4A: add x-axis

(Remarks on code availability)

Version 1:

Reviewer comments:

Reviewer #1

(Remarks to the Author)
I think the authors have addressed most of my concerns. My only remaining concern is the machine learning part. They used only 36 samples to train models with 26 input genes – that is too few samples for reliable machine learning. The results from this analysis are not very robust and add little beyond simpler statistical tests. The authors could have achieved the same conclusion (DR5 is a key gene) through differential expression and correlation analyses alone. The addition of 15 machine learning algorithms gives a superficial impression of sophistication but does not fundamentally advance the mechanistic understanding. The main biological conclusions do not depend on the ML, so I am not asking them to remove it. However, I think they should honestly acknowledge this limitation in the Discussion section.

(Remarks on code availability)
The code include a lot of Chinese documentations, and is a bit messy to follow. It looks like a credible code history from the author, but I don't think I can reproduce everything from it.

Reviewer #2

(Remarks to the Author)
The authors have made substantial and careful revisions that fully address my previous concerns. The manuscript now presents a compelling, well-supported, and innovative mechanism linking glycerophospholipid metabolism to hepatocyte pyroptosis via the DR5-FXR-CHPT1 axis following major partial hepatectomy. The evidence is now solid, and the conclusions are appropriately supported. I have no further concerns and recommend acceptance of this revised manuscript for publication in Nature Communications.

(Remarks on code availability)

Reviewer #3

(Remarks to the Author)

(Remarks on code availability)

Open Access This Peer Review File is licensed under a Creative Commons Attribution 4.0 International License, which permits use, sharing, adaptation, distribution and reproduction in any medium or format, as long as you give appropriate credit to the original author(s) and the source, provide a link to the Creative Commons license, and indicate if changes were made.
In cases where reviewers are anonymous, credit should be given to 'Anonymous Referee' and the source.
The images or other third party material in this Peer Review File are included in the article's Creative Commons license, unless indicated otherwise in a credit line to the material. If material is not included in the article's Creative Commons license and your intended use is not permitted by statutory regulation or exceeds the permitted use, you will need to obtain permission directly from the copyright holder.
To view a copy of this license, visit https://creativecommons.org/licenses/by/4.0/

Response Letter (Manuscript # NCOMMS-26-018614A)

Dear Editor and Reviewers,

We thank you very much for the thorough and thoughtful evaluation of our manuscript “Multi-omics reveals the DR5-FXR-CHPT1 axis governs hepatocyte pyroptosis via glycerophospholipid remodeling after major partial hepatectomy” (Manuscript # NCOMMS-26-018614A).

We greatly appreciate the reviewers’ insightful comments and constructive suggestions, which have helped us to substantially improve the rigor, clarity, and overall quality of the work.

We have carefully revised the manuscript in accordance with the suggestions provided. We are pleased to submit a revised version for your consideration which we believe now meets the expectations of the reviewers and journal. Detailed, point-to-point responses are provided in the sections that follow.

Thank you again for your time and insightful suggestions. Please do not hesitate to contact me if any additional information is required

Yours Sincerely,

Hua Li MD. & Ph.D

Department of Hepatic Surgery, Liver Transplantation Center,

the Third Affiliated Hospital of Sun Yat-Sen University, Guangzhou 510630, China

E-mail: lihua3@mail.sysu.edu.cn

Response to Reviewers' Comments

BLUE TEXT: Reviewer comments

BLACK TEXT: Author responses

RED TEXT: Changes made to text in manuscript

Reviewer #1 (Remarks to the Author)

Comments to the Authors

This manuscript addresses the mechanism of liver injury after major partial hepatectomy (PHx) through an integrative multi-omics framework, combining human blood data with multiple mouse datasets, including single-cell transcriptomics, bulk RNA-seq, lipidomics, and proteomics. Overall, the study is ambitious, and the broad experimental design appears potentially valuable. The proposed DR5–FXR–CHPT1 axis is interesting and may be relevant to understanding hepatocyte pyroptosis and glycerophospholipid remodeling after PHx. However, several major issues currently prevent a proper assessment of the methodological rigor and robustness of the conclusions.

1. Sample size and cohort information are insufficiently reported throughout the manuscript.

This is my main concern. It is currently very unclear how many biological samples were included in each analysis, and without this information it is not possible to evaluate the statistical robustness or reliability of the findings. For example, the number of samples used for WGCNA should be clearly stated, as this is particularly important for assessing the validity of module identification and correlation analyses.

If the sample size is small, especially below the range typically considered suitable for WGCNA, the reliability of the network analysis becomes questionable. The authors should clearly report sample numbers, biological replicates, and the exact datasets used for every experiment and analysis.

Response: We sincerely thank the reviewer for pointing out this critical omission. We fully agree that transparently reporting sample sizes and cohort information is essential for evaluating the statistical robustness and reliability of our findings. In the revised manuscript, we have updated all **figure legends** to explicitly report the exact sample numbers, biological replicates, and the specific datasets used for every single experiment and analysis. Furthermore, we have introduced a comprehensive new supplementary table (Supplementary information-1.4 Data and biological samples) that meticulously details the cohort information and sample sizes across the entire study. Regarding the statistical robustness of our bioinformatics analyses, we would like to explicate the following points:

For WGCNA: We utilized a total of 36 samples from our in-house 70% PHx RNA-seq dataset. This sample size far exceeds the recommended minimum threshold (typically >15 samples) required for building a stable WGCNA network, thereby ensuring high reliability and validity in our module identification and correlation analyses.

For Machine Learning[1,2]: Our training set consisted of these same 36 internal samples. To further guarantee the stability and generalizability of our results, we validated our findings across four independent external datasets (GSE151309, GSE20426,

GSE181476, and CNP0002310[3]). We believe this substantial cumulative sample size across diverse validation cohorts provides more than ample statistical power to support our conclusions. We hope these comprehensive updates adequately address your concerns regarding the statistical robustness of our analyses.

1.4 Data and biological samples

Data	Biological samples
RNA-seq (training set)	n=36
GSE151309	n=5
GSE20426	n=35
GSE181476	n=14
CNP0002310	n=20
90% PHx (scRNA-seq)	n=3
Proteomics	n=24
Lipidomics	n=24

2. The manuscript shifts between 70% PHx and 90% PHx in a way that makes the study design difficult to follow.

The logic for when 70% PHx versus 90% PHx is used is not sufficiently clear. At present, the results section appears to move between these two models without a clear framework, which makes it difficult to determine whether the conclusions are intended to apply to one condition, the other, or both. This also raises concern about selective presentation of results. The authors should either focus primarily on one PHx model for the main mechanistic conclusions or present the two conditions in parallel in a more systematic and consistent manner.

Response: We sincerely thank the reviewer for pointing out the lack of clarity regarding our parallel use of the 70% and 90% PHx models. We completely agree that

a well-defined logical framework is essential to prevent any concerns regarding the selective presentation of data. Accordingly, we have now explicitly clarified the rationale in the revised manuscript: the classical 70% PHx model was utilized to dissect and establish the foundational mechanism, whereas the 90% massive hepatectomy model was employed as an extended, lethal model. The latter allowed us to rigorously validate the universality and clinical relevance of the pyroptotic cascade in post-hepatectomy liver failure (PHLF) and to effectively evaluate the capacity of our interventions (e.g., WY-14643)[4] to rescue lethal outcomes.

Following your constructive suggestion to present the two conditions in a more systematic and parallel manner, we sought to eliminate the possibility of model-specific bias and determine whether this mechanism is universally applicable to more severe, lethal PHLF. To this end, we performed a parallel, systematic validation of our core findings using the 90% PHx model. Strikingly consistent with the trends observed in the 70% PHx model, Western blot analysis revealed that the expression of core pyroptosis pathway proteins was significantly upregulated in a time-dependent manner, peaking at D3 (Response Figure 1A). Concurrently, ELISA confirmed a drastic and progressive systemic release of the downstream proinflammatory cytokines IL-1β and IL-18 (Response Figure 1B). Furthermore, transmission and scanning electron microscopy (TEM and SEM) unambiguously captured the hallmark ultrastructural features of pyroptosis (Response Figure 1C). Collectively, these parallel validation results conclusively demonstrate that the NLRP3/Caspase-1/GSDMD-mediated pyroptosis is not an idiosyncrasy of the 70%

resection model, but rather a universal and critical pathological driver of acute liver injury following extensive hepatectomy.

We have now explicitly distinguished the rationales for employing these two models in the revised text to avoid any potential misunderstandings regarding selective data presentation. We have incorporated these new validation data into Supplementary Figure 1J-M, and the Results section has been revised accordingly (Line 228-242): "Having thoroughly characterized the pyroptotic process in the classical 70% PHx model, we next sought to eliminate the possibility of model-specific bias and determine whether this mechanism is universally applicable to more severe, lethal PHLF. To this end, we performed a parallel, systematic validation of our core findings using a 90% PHx model. Strikingly consistent with the trends observed in the 70% PHx model, WB analysis revealed that the expression of core pyroptosis pathway proteins was significantly upregulated in a time-dependent manner, peaking at D3 (Supplementary Figure 1J-K). Concurrently, ELISA confirmed a drastic and progressive systemic release of the downstream proinflammatory cytokines IL-1β and IL-18 (Supplementary Figure 1L). Furthermore, TEM and SEM unambiguously captured the hallmark ultrastructural features of pyroptosis (Supplementary Figure 1M). Collectively, these parallel validation results conclusively demonstrate that the NLRP3/Caspase-1/GSDMD-mediated pyroptosis is not an idiosyncrasy of the 70% resection model, but rather a universal and critical pathological driver of acute liver injury following extensive hepatectomy."

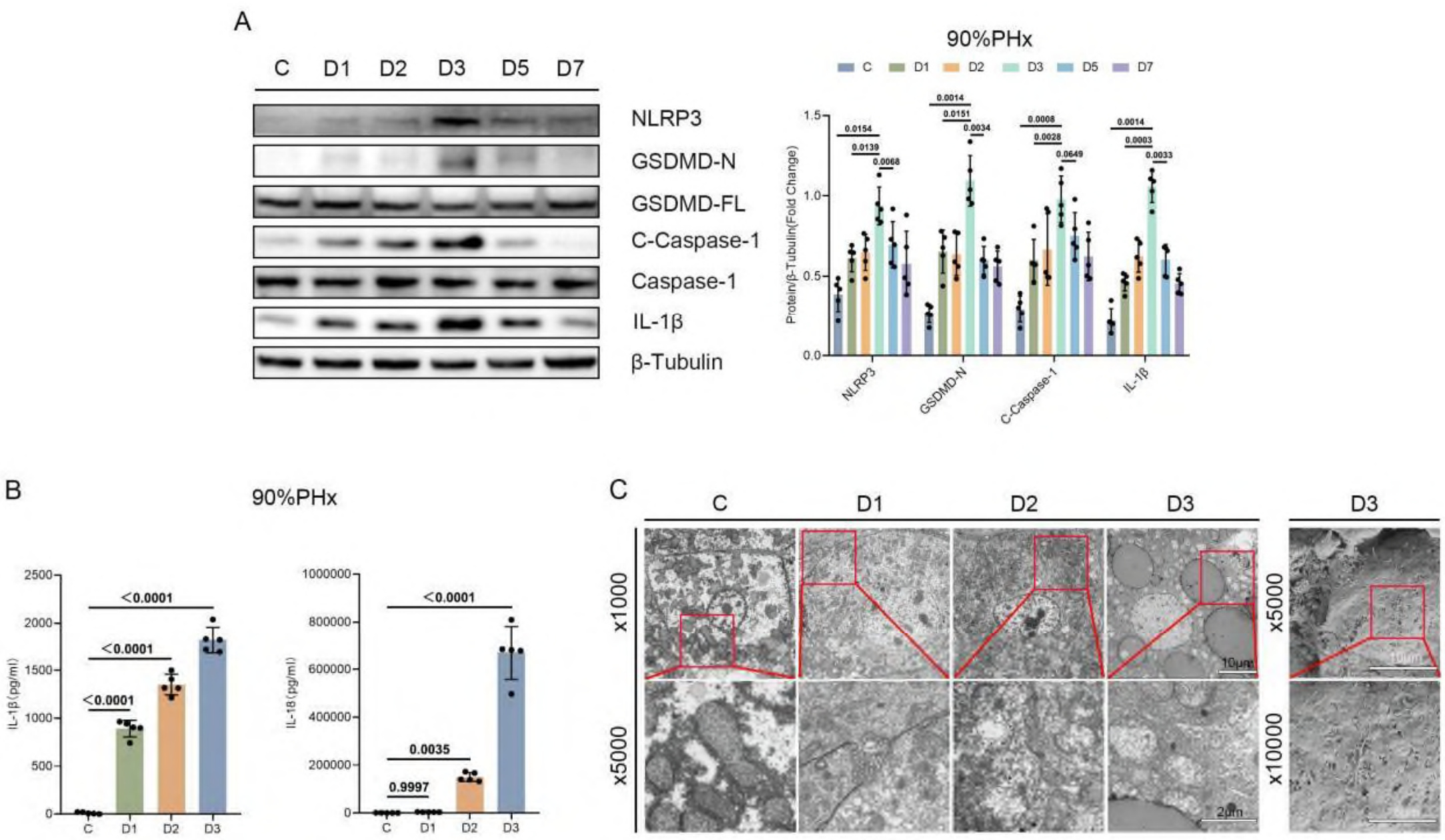

Response Figure 1. A: (left panel) Representative western blot images of pyroptosis-related proteins in liver tissue following 90% PHx (n=5 independent experiments); (right panel) Quantitative statistical analysis of protein expression levels (n=5 mice per group). B: Serum levels of IL-1β and IL-18 cytokines measured by ELISA following 90% PHx (n=5 mice per group). C: Representative Transmission Electron Microscopy (TEM) and Scanning Electron Microscopy (SEM) images of liver tissue following 90% PHx (Scale bar=10 μm. n=3 independent experiments).

3. The machine learning analysis is inadequately described.

The manuscript refers to machine learning methods, but the modeling framework is not defined clearly enough for evaluation or reproduction. It is not clear what the objective of the model is, what the input features are, what the output variable is, or whether the model is performing classification or regression. If it is a classification model, the classes should be explicitly defined. In addition, the training, validation, and testing procedures, performance metrics, and methods for feature selection should all be described in detail. At present, the analysis is not interpretable, and reproducibility is further limited by the lack of code and data availability.

Response: We sincerely thank the reviewer for this valuable and highly constructive

feedback. We fully agree that the original manuscript lacked sufficient detail on the machine learning framework. In the revised manuscript, we have substantially expanded the Methods section and have also deposited the complete R code and all relevant data to ensure full transparency and reproducibility (https://github.com/tys980915/code.git). Below we address each specific point:

1. Model objective: classification vs regression

Our machine learning model was designed as a binary classification task.

Objective: To identify genes that can best discriminate between pre-operative samples and post-hepatectomy injury samples (different injury stages), and to rank gene importance for pinpointing key regulators of pyroptosis and lipid metabolism.

Output variable (labels):

Positive class (1): Disease (post-PHx samples, specifically D1, D2, or D3 after 70% PHx).

Negative class (0): Control (C, pre-surgery).

In the code, type <- "Disease" defines the positive class.

Input features: Expression values of 26 candidate genes – the intersection of DEGs from bulk RNA-seq, scRNA-seq, WGCNA modules, and pyroptosis-related genes (Figure 4E), (Expression values of 20 candidate genes-Supplementary Figure 5A).

Clarified in Methods – Machine learning, subsection “Model objective and input features”.

2. Training, validation, and testing procedures

We have now explicitly detailed the data split and validation strategy (see code lines:

fold <- 5, training on DatasetA.txt, testing on other independent datasets).

Training set: Our own bulk RNA-seq dataset (n=36 paired samples) – file DatasetA.txt.

External test sets (used to evaluate generalizability):

GSE151309, GSE20426, GSE181476, CNP0002310 (each converted to pseudo-bulk or used as is).

Internal validation during training: 5-fold cross-validation (CV) repeated once (code: fold <- 5). For each CV fold, 80% of training data was used for model fitting and 20% for internal validation.

Feature selection: Lasso regression (cv.glmnet with alpha=1, 5-fold CV) was first applied to reduce the 26 features to a smaller subset of informative genes (stored as lassogene). This was done before running most downstream algorithms (e.g., LDA, QDA, KNN, DT, RF, XGBoost, SVM, GBM, Naive Bayes).

No separate validation set – we relied on cross-validation for hyperparameter tuning and used external datasets for final evaluation.

3. Algorithms, hyperparameter tuning, and performance metrics

We used 15 algorithms as originally stated. The code shows that for algorithms with multiple hyperparameters (e.g., neural network, XGBoost, GBM, SVM), we performed grid search within cross- validation. Key examples:

Algorithm	Hyperparameters tuned	Tuning method
Neural network (MLP)	batch size, learning rate, dropout rate, cutoff	Grid search (nested loops)

XGBoost	nrounds, eta, max_depth	caret::train with grid (50–1000 nrounds, eta 0.025–0.3, max_depth 2–6)
Random Forest	mtry (25th, 50th, 75th percentile of gene number)	Iteration
KNN	k (1–5)	Iteration
SVM	kernel (linear, polynomial, radial) + cost/gamma via caret	caret::train with 5-fold CV
GBM	interaction.depth, n.trees, shrinkage, n.minobsinnode	caret::train with grid
Logistic regression, LDA, QDA, Ridge, Lasso, Elastic Net, Decision Tree, Naive Bayes	Cutoff (0.25, 0.5, 0.75) or default	Minimal tuning

Performance metrics (calculated for each model on each test dataset and on the training set):

- Accuracy (result_acc)
- Recall (Sensitivity) (result_recall)
- F1-score (result_FS) – computed using a custom f1_score function (source

file F1S.R).

The final ranking of gene importance (Figure 4F, Supplementary Figure 5D) was based on the Random Forest model (mean decrease in Gini index) and also cross- checked with XGBoost and Lasso- logistic regression coefficients.

4. Interpretability and feature importance

Extracted variable importance from the final Random Forest model (importance(model)). Also extracted coefficients from Lasso (non-zero coefficients) and importance from XGBoost (xgb.importance). Ranked all 26 genes by importance; DR5 was the top 3 gene in all three methods. The heatmaps show the performance of each model across training and test sets, demonstrating that models using Lasso-selected features often performed better than full-feature models.

We have expanded the corresponding "Methods" section in the revised manuscript (Line 917-971): "Machine Learning Model Objective and Feature Selection

To identify key regulators of pyroptosis and lipid metabolism, a binary classification machine learning framework was constructed[1,2]. The primary objective was to screen for genes that optimally discriminate between pre-operative control samples and post-hepatectomy injury samples across different surgical stages, and to rank these genes based on their predictive importance. The output variable was defined as a binary label: the positive class (1) represented disease states (post-partial hepatectomy [PHx] samples at D1, D2, or D3 after 70% PHx), while the negative class (0) represented pre-surgery controls (C). The input features consisted of the expression profiles of 26 candidate genes, derived from the intersection of

differentially expressed genes (DEGs) from bulk RNA-seq, single-cell RNA-seq (scRNA-seq), Weighted Gene Co-expression Network Analysis (WGCNA) modules, and well-defined pyroptosis-related genes. A parallel evaluation utilizing a subset of 20 candidate genes was also performed.

Training, Validation, and Testing Strategy

Model training was conducted using an internal bulk RNA-seq dataset comprising 36 samples (n=36 paired samples derived from 18 mice). To rigorously evaluate the generalizability of the models, four independent external datasets (GSE151309, GSE20426, GSE181476, and CNP0002310) were utilized as independent test sets. For single-cell datasets within this testing cohort, data were converted to pseudo-bulk expression profiles prior to model evaluation. During the training phase, a 5-fold cross-validation (CV) strategy was employed, wherein the training data was iteratively partitioned into 80% for model fitting and 20% for internal validation. Prior to downstream classification, Lasso (Least Absolute Shrinkage and Selection Operator) regression (alpha=1, via 5-fold CV) was applied to select an optimal subset of informative features and reduce dimensionality.

Algorithms and Hyperparameter Tuning

A comprehensive panel of 15 machine learning algorithms was implemented. For algorithms necessitating hyperparameter optimization, grid search strategies were executed within the cross-validation framework. Specifically, the Multi-Layer Perceptron (MLP) neural network was tuned for batch size, learning rate, dropout rate, and cutoff values. Extreme Gradient Boosting (XGBoost) models were optimized

across learning rates (eta: 0.025-0.3), maximum depth (2-6), and iterations (nrounds: 50-1000). Support Vector Machine (SVM) models evaluated linear, polynomial, and radial kernels alongside cost/gamma parameters. Gradient Boosting Machine (GBM) parameter grids included interaction depth, number of trees, shrinkage, and minimum observations in terminal nodes. Random Forest models were tuned by iterating mtry values corresponding to the 25th, 50th, and 75th percentiles of the feature count, while k-Nearest Neighbors (KNN) evaluated k values ranging from 1 to 5. Other algorithms, including Logistic Regression, Linear Discriminant Analysis (LDA), Quadratic Discriminant Analysis (QDA), Ridge Regression, Elastic Net, Decision Trees, and Naive Bayes, were applied using default configurations or evaluated across standard probability thresholds (0.25, 0.5, 0.75).

Performance Evaluation and Model Interpretability

Model performance across both the internal training cohort and external test sets was quantified using Accuracy, Recall (Sensitivity), and F1-score. To ensure biological interpretability and pinpoint key regulatory genes, feature importance was extracted and aggregated from multiple high-performing models. This included the Mean Decrease in Gini index from the Random Forest model, the gain metrics from the XGBoost algorithm, and the non-zero coefficients derived from the Lasso regression. The candidate genes were subsequently ranked based on their integrated importance scores, consistently identifying DR5 among the top three key regulators across the utilized methodologies. Data: GSE151309 (data2), GSE20426 (data3), GSE181476 (data4), and CNP0002310 (data5).”

To fully address your concern regarding reproducibility, we have made all raw and processed datasets, as well as the complete, well-commented R scripts required to reproduce this machine learning pipeline, publicly available."

4. The novelty and significance of the phosphatidylcholine-related findings need to be clarified.

A substantial part of the manuscript focuses on the role of phosphatidylcholine (PC) in PHx-induced liver injury. However, the authors themselves note in the Introduction that this aspect has already been relatively well described in the literature. Therefore, it is unclear what the genuinely new conceptual advance is here. The authors should better distinguish their work from prior studies and explain more clearly what this study adds beyond the existing knowledge base.

Response: We sincerely thank the reviewer for giving us the opportunity to clarify the conceptual advance of our study. While previous lipidomic profiling has indeed cataloged the extensive disruption of phosphatidylcholine (PC) following partial hepatectomy (PHx), we emphasize that these existing findings have largely remained at a purely descriptive and phenomenological level[5]. The upstream molecular drivers orchestrating this metabolic reprogramming, as well as the precise consequences of PC dysregulation on cell death execution, have remained completely undefined prior to our study.

By integrating multi-omics data with functional experiments, we provide the first evidence that DR5 drives this PC metabolic remodeling. Mechanistically, DR5

inhibits the transcription factor FXR, thereby downregulating the expression of CHPT1, the rate-limiting enzyme for PC synthesis. This upstream regulatory mechanism has never been previously reported in the context of PHx. Specifically, we reveal the DR5-FXR-CHPT1 axis as the central upstream engine driving hepatic PC depletion. Crucially, we move beyond associative observations by demonstrating that this specific lipidomic collapse is not merely a byproduct of hepatic injury, but a direct instigator of endoplasmic reticulum stress (ERS)-mediated pyroptosis. By fundamentally bridging the gap between glycerophospholipid metabolism and inflammatory cell death, our work provides a novel mechanistic framework and identifies actionable therapeutic targets for mitigating post-hepatectomy liver failure (PHLF).

To help readers accurately grasp the genuine conceptual advance our study offers beyond the existing knowledge base, we have now explicitly articulated this novelty in the **Introduction** and **Discussion** sections of the revised manuscript.

Corresponding Revisions in the Manuscript

In the Introduction (Line 127-133): "Although the dynamic changes in PC following PHx are relatively well-documented, and a decreased hepatic PC/PE ratio has been identified as a predictive marker for survival following PHx, existing studies are largely confined to phenomenological observations. Therefore, the upstream drivers of these glycerophospholipid metabolic alterations, their underlying molecular mechanisms, and the specific impact of glycerophospholipid metabolites on injury in the remnant liver require further investigation."

In the Discussion (Line 460-464): "We reveal the DR5-FXR-CHPT1 axis as the core pathway orchestrating hepatic PC metabolic reprogramming post-hepatectomy. Importantly, we show that severe PC depletion triggers lethal endoplasmic reticulum stress (ERS), subsequently activating GSDMD-mediated pyroptosis."

5. Functional validation of DR5 appears incomplete.

The manuscript seems to rely primarily on DR5 silencing to support the proposed mechanism. While loss-of-function data are useful, complementary gain-of-function experiments would substantially strengthen the causal interpretation. For example, DR5 overexpression in vivo or in vitro could help determine whether increased DR5 is sufficient to induce the expected phenotype. If the current dataset is sufficiently powered and the authors believe silencing alone is adequate, this needs to be justified more clearly. Again, this is difficult to assess without clear reporting of sample sizes.

Response: We highly appreciate the reviewer's rigorous standard for establishing causality. We fully agree that while loss-of-function data are informative, complementary gain-of-function experiments are indispensable for substantially strengthening the causal interpretation of the proposed mechanism.

To directly address this, rather than merely justifying the adequacy of silencing, we have now comprehensively supplemented both in vitro and in vivo gain-of-function experiments. We have also ensured that sample sizes (n) and statistical significance are explicitly and prominently reported across all these

functional assays.

1. In vitro functional validation (Knockdown and Overexpression)

In the revised manuscript, we fully present the qRT-PCR and Western blot validation data for DR5 knockdown (DR5-KD) and DR5 overexpression (DR5-OE) cell lines (Response Figure 2A-F). Furthermore, we respectfully direct the reviewer's attention to the in vitro gain-of-function functional experiments that were performed. Specifically, utilizing the DR5-OE model, we demonstrated that DR5 overexpression actively exacerbates pyroptosis and further aggravates downstream cellular damage when combined with an FXR inhibitor. Concurrently, DR5-OE successfully downregulated CHPT1 expression, strongly supporting its upstream regulatory role (Response Figure 2G).

2. In vivo gain-of-function and rescue experiments

To definitively fortify the causal interpretation in vivo, we engineered an adeno-associated viral vector to overexpress DR5 (AAV-DR5-OE) and validated its robust hepatocyte tropism and transduction efficiency in the 70% PHx mouse model (Response Figure 2L-O). Strikingly, AAV-DR5-OE administration markedly decreased mouse survival, a lethal outcome that was effectively mitigated by co-treatment with the FXR agonist GW4064 (Response Figure 2P). Concurrently, AAV-DR5-OE-treated mice exhibited drastically elevated serum ALT and AST levels (Response Figure 2Q-R) alongside a substantially increased area of hepatic damage. Activation of FXR with GW4064 markedly ameliorated these liver injuries, partially abrogating the detrimental impacts of DR5 overexpression.

At the ultrastructural level (SEM and TEM), AAV-DR5-OE exacerbated extensive plasma membrane pore formation, severe sinusoidal congestion, and mitochondrial swelling, whereas GW4064 co-treatment effectively preserved organelle integrity. Mechanistically, Western blot analysis revealed that AAV-DR5-OE suppressed CHPT1 expression and triggered a massive upregulation of core pyroptosis markers (NLRP3, GSDMD-N, Cleaved Caspase-1, and IL-1β). Notably, GW4064 intervention reversed these molecular alterations, restoring CHPT1 levels while effectively dampening the pyroptotic protein signature (Response Figure 2U-V). Collectively, these robust gain-of-function data, coupled with our loss-of-function experiments, provide compelling and sufficient in vivo evidence establishing the DR5-FXR-CHPT1 axis as a causal driver of pyroptosis post-PHx. We have incorporated these new validation data into the **Results** section (Line 358-361): "We established two distinct DR5-knockdown (DR5-KD) THLE-2 cell lines (Supplementary Figure 5F) and observed that DR5-KD significantly reduced pyroptosis-related protein expression in vitro (Figure 4I, Supplementary Figure 5G).

(Line 374-376): In parallel, we established a DR5-overexpressing (DR5-OE) THLE-2 cell line (Supplementary Figure 6I-J), DR5-OE increased pyroptosis, which was mitigated by GW4064 (Figure 5M).

(Line 416-419): To evaluate this axis in vivo, mice were administered an AAV-DR5-KD vector featuring a TBG promoter for hepatocyte-specific targeting. We

confirmed its precise liver tropism and potent silencing efficiency in the 70% PHx model (Figure 6I, Supplementary Figure 7F-J).

(Line 429-445): To further substantiate these findings, an AAV-DR5-OE vector with verified liver-specific tropism and high transduction efficiency was employed in the 70% PHx model (Supplementary Figure 7M-Q). AAV-DR5-OE treatment markedly decreased mouse survival, whereas FXR activation via GW4064 co-treatment mitigated this escalating mortality trend (Figure 6N). Concurrently, AAV-DR5-OE-treated mice exhibited drastically elevated serum ALT and AST levels (Figure 6O, Supplementary Figure 7R) alongside a substantially increased area of hepatic damage (Figure 6P-Q). Activation of FXR with GW4064 markedly ameliorated these liver injuries, partially abrogating the detrimental impacts of DR5 overexpression. SEM and TEM imaging further confirmed that GW4064 effectively maintained organelle integrity, counteracting the AAV-DR5-OE-induced aggravation of plasma membrane poration, sinusoidal congestion, and mitochondrial swelling. Mechanistically, WB analysis revealed that AAV-DR5-OE suppressed CHPT1 expression and triggered the massive upregulation of pyroptosis markers (NLRP3, GSDMD-N, Cleaved Caspase-1, and IL-1β). Notably, GW4064 intervention reversed these molecular alterations, restoring CHPT1 levels while effectively dampening the pyroptotic protein signature (Figure 6R, Supplementary Figure 7S)."

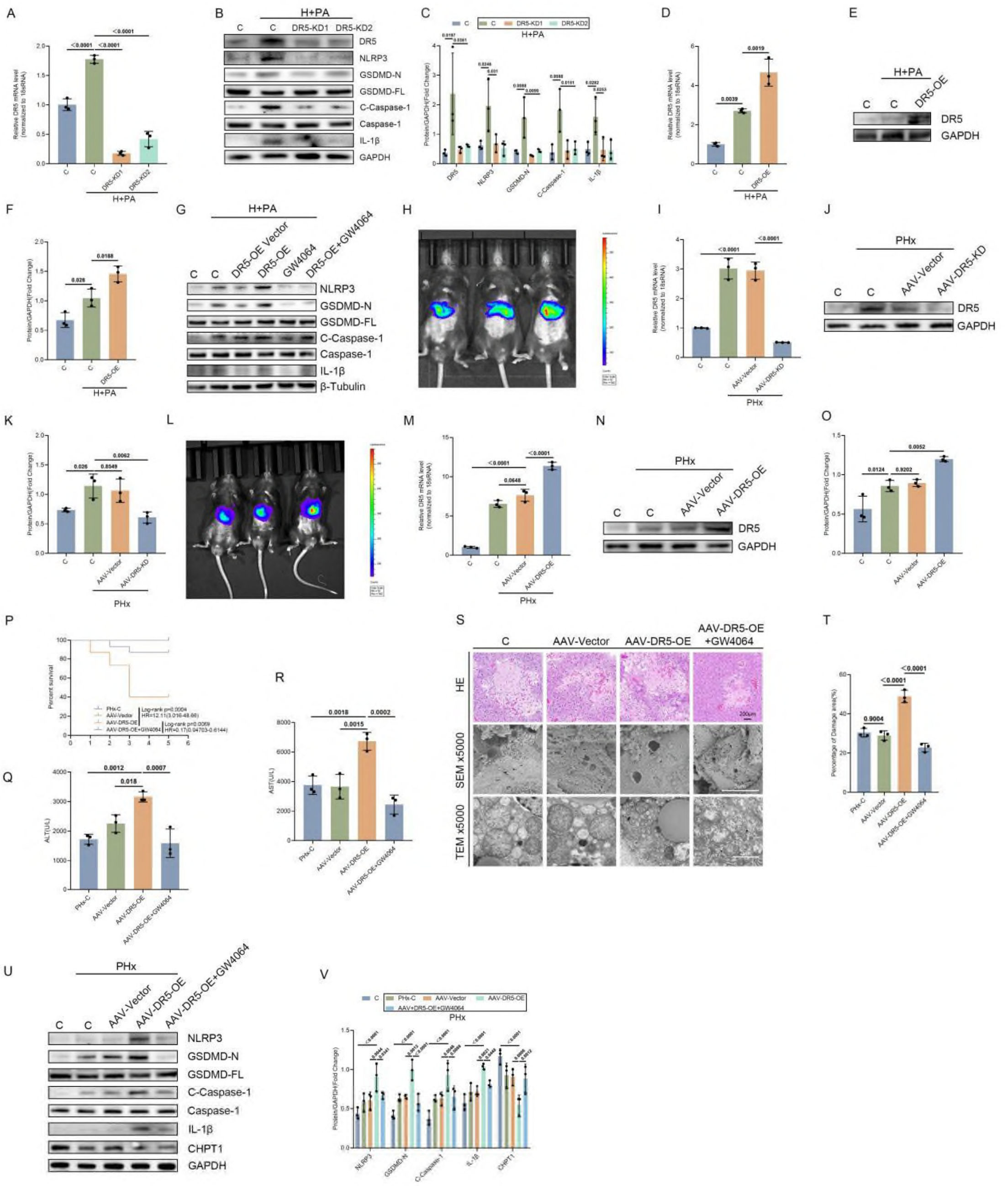

Response Figure 2. A: RT-qPCR validation of DR5 expression levels following DR5 knockdown (n=3 independent experiments). B: Representative western blot analysis of pyroptosis-related proteins in the cell model following DR5 knockdown (DR5-KD) (n=3 independent experiments). C: Quantitative statistical analysis of protein expression levels corresponding to Response Figure 2B (n=3 independent experiments). D: RT-qPCR validation of DR5 mRNA expression levels following DR5 overexpression (n=3 independent experiments). E: Representative western blot images of DR5 protein expression levels. F: corresponding quantification following DR5 overexpression in the cell model (n=3 independent experiments). G: Representative western blot images of pyroptosis-related proteins in the cell model following DR5 overexpressing (DR5-OE) combined with the FXR agonist GW4064 (n=3 independent experiments). H: In vivo imaging showing specific targeted

infection and expression in the liver region following AAV-DR5-KD virus injection (n=3 per group). I: RT-qPCR validation of DR5 mRNA expression levels following AAV-DR5-KD administration (n=3 independent experiments). J: Representative western blot images of DR5 protein expression levels following AAV-DR5-KD administration (n=3 independent experiments). K: Quantitative statistical analysis of protein expression levels corresponding to Response Figure 2J (n=3 independent experiments). L: In vivo imaging showing specific targeted infection and expression in the liver region following AAV-DR5-OE virus injection (n=3 per group). M: RT-qPCR validation of DR5 mRNA expression levels following AAV-DR5-OE administration (n=3 independent experiments). N: Representative western blot images of DR5 protein expression levels following AAV-DR5-OE administration (n=3 independent experiments). O: Quantitative statistical analysis of protein expression levels corresponding to Response Figure 2N (n=3 independent experiments). P: Survival curve of the 70% PHx treated with AAV-DR5-OE or GW4064 (n=15 per group. Survival curves were generated using the Kaplan-Meier method, and statistical significance between groups was determined using the Log-rank test). Q: Serum ALT levels following 70% PHx in each treatment group (n=3 per group). R: Serum AST levels in each group (n=3 per group). S: Representative H&E (Scale bar=200 μm), SEM (Scale bar=5 μm), and TEM (Scale bar=2 μm) images of each treatment group following 70% PHx (n=3 independent experiments). T: Statistical analysis of liver damage area in each treatment group (n=3 per group). U: Representative western blot analysis of CHPT1 expression and pyroptosis-related proteins following 70% PHx in each treatment group (n=3 independent experiments). V: Quantitative statistical analysis of protein expression levels corresponding to Response Figure 2U (n=3 per group).

Minor comments

6. Line 147:

The sentence beginning “While DR5 has frequently been targeted in therapies for solid tumors…” appears incomplete and should be revised for clarity.

Response: We sincerely apologize for this grammatical omission. We have carefully revised the sentence in the revise manuscript to ensure clarity and completeness. The corrected sentence now reads as follow, we have incorporated this revision into the Introduction section (Line 150-154): “While DR5 has frequently been targeted in therapies for solid tumors (including melanoma, breast, lung, and colon cancers), its

role in benign liver disease models remains notably unexplored, particularly regarding hepatocyte injury in the remnant liver after massive hepatectomy."

7. Line 221:

The authors report Oil Red O staining confirming intrahepatic lipid accumulation following 70% PHx. Given the broader comparison between 70% and 90% PHx, it would be helpful to also show the corresponding staining results for 90% PHx.

Response: We highly appreciate this constructive suggestion. We fully agree that providing the corresponding lipid staining results for the 90% PHx model is crucial for a comprehensive comparison. Accordingly, we have now performed Oil Red O staining on liver sections from the 90% PHx mouse model. The representative images, alongside the corresponding quantitative analysis, have been provided for the reviewer's evaluation (Response Figure 3). This new addition visually and unambiguously demonstrates that intrahepatic lipid accumulation and metabolic disorder are markedly more severe in the 90% PHx model compared to the 70% PHx model. These functional findings are highly consistent with the escalated lethality and the aggravated pyroptotic cascade observed in the 90% PHx cohort, thereby further consolidating our overall mechanistic conclusions.

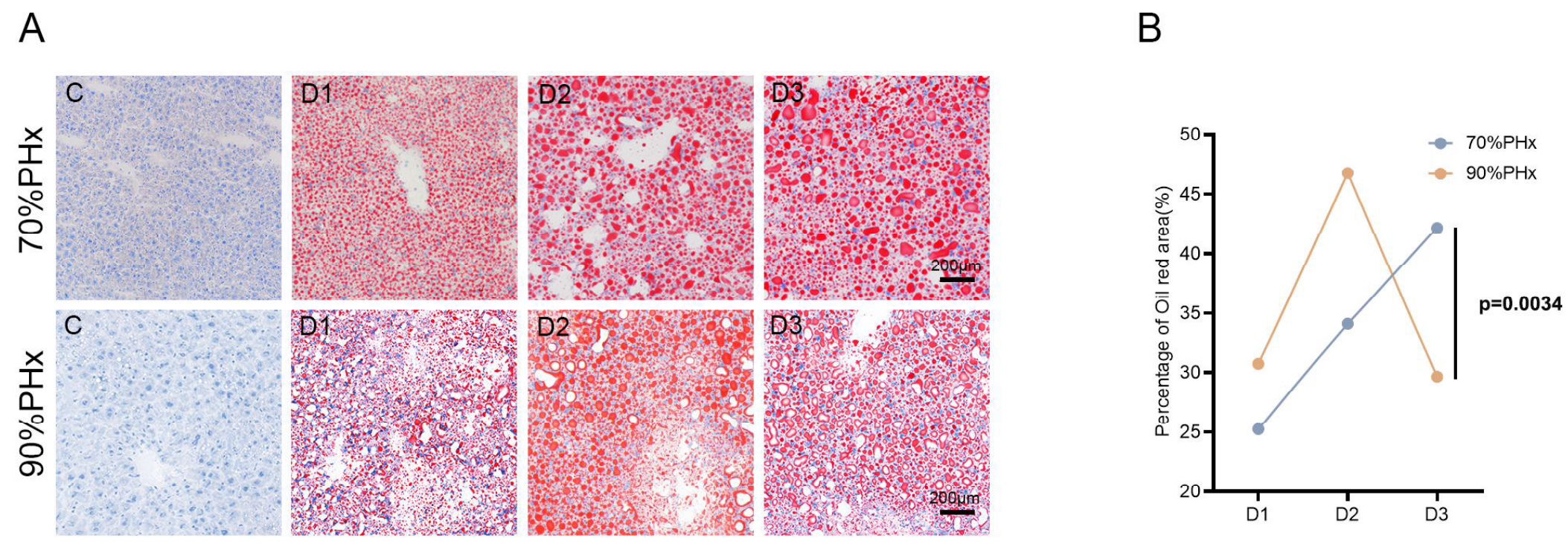

Response Figure 3. A: Representative Oil Red O staining images of liver tissue following 70% PHx and 90% PHx (Scale bar=200 μm, n=5 independent experiments). B: Quantitative statistical analysis of Oil Red O staining corresponding to Response Figure3A.

8. Line 303:

The statement that "The Brown and Green modules showed high Pearson correlation coefficients (>0.7) with PHx, suggesting their importance" is not sufficiently justified. A strong correlation does not by itself establish biological importance. The authors should either rephrase this claim more cautiously or provide stronger reasoning and supporting evidence.

Response: We highly appreciate the reviewer's rigorous scientific perspective. We fully agree that a strong statistical correlation does not inherently equate to biological importance or causality. To address this valid concern and provide stronger supporting evidence, we have performed further biological functional enrichment analyses on these specific modules. The results demonstrated that the Brown and Green modules are predominantly enriched in pathways associated with postoperative injury and tissue repair responses (Supplementary Figure 4A). This provides the essential biological context required to support their functional relevance beyond mere

statistical correlation. Furthermore, in accordance with your constructive suggestion, we have rephrased the original claim to be more scientifically cautious. The revised text in the manuscript (Line 343-347): "The Brown and Green modules showed high Pearson correlation coefficients (>0.7) with PHx, subsequent biological functional analysis revealed that these modules are highly associated with postoperative injury and repair responses. Consequently, they were prioritized for the screening of key genes."

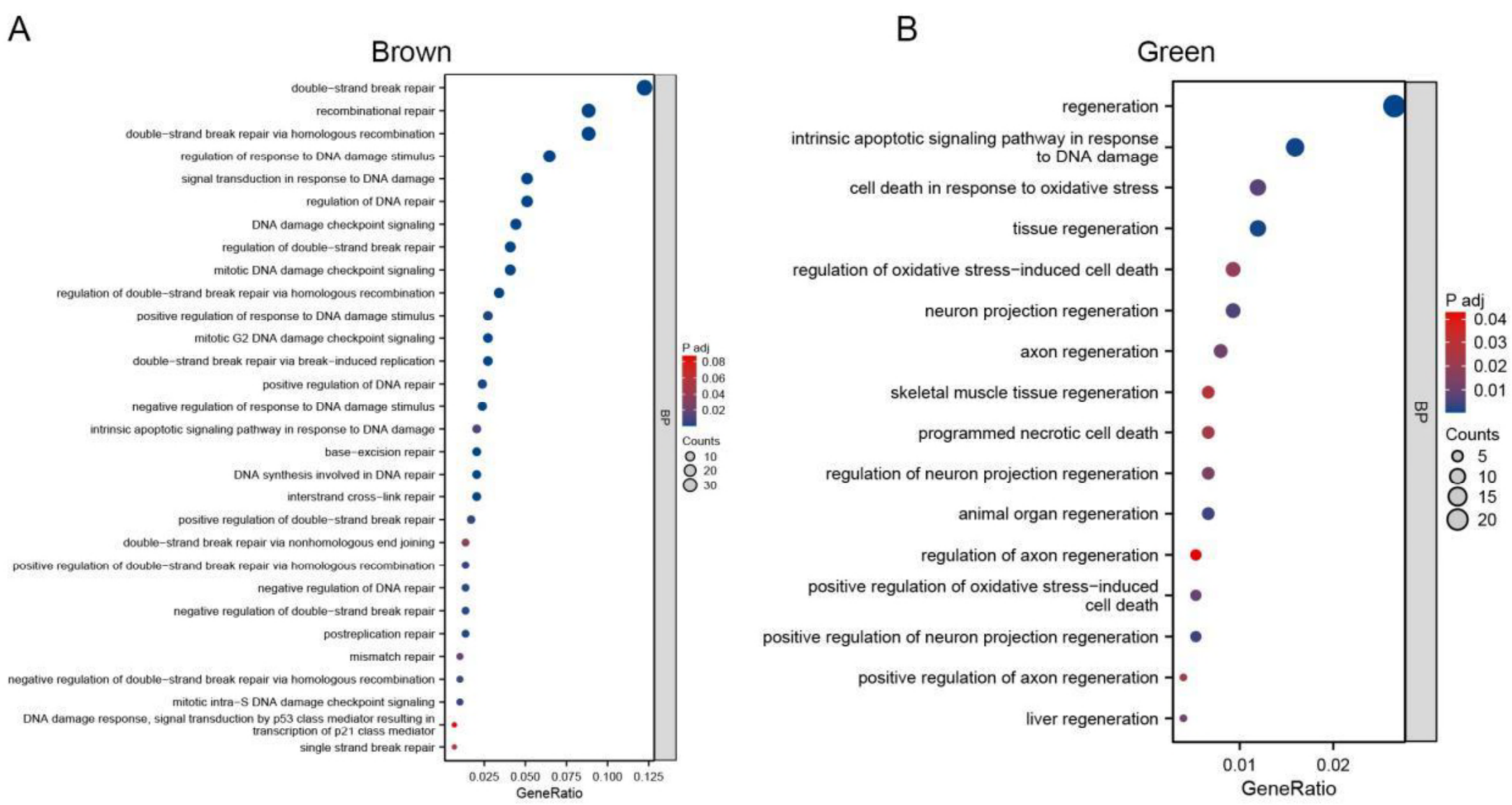

Response Figure 4. A: Functional enrichment analysis of the Brown modules. B: Functional enrichment analysis of the Green modules.

9. Pseudotime analysis is insufficiently described.

The manuscript refers to pseudotime analysis multiple times, but I could not find an adequate description of this analysis in the Methods. The authors should explain how pseudotime was inferred, which cells were included, what software/package was used, and how the resulting trajectories were interpreted.

Response: We sincerely thank the reviewer for their careful evaluation and for raising

this constructive point. We realize that our description in the original manuscript (Figure 3A and Figure 5B) was unfortunately ambiguous, which understandably led to the misconception that we performed "single-cell sequencing pseudotime trajectory inference." We deeply apologize for this lack of clarity. To clarify, this was not a single-cell pseudotime analysis. Rather, it was a time-series K-Means clustering analysis performed on bulk lipidomic and proteomic data across the various time points of our animal model[6,7].

To explore the dynamic evolutionary patterns of lipid and protein components during the acute injury phase, we inputted the expression matrices of significantly altered lipids and proteins across the PHx time points (C, D1, D2, D3). Based on the Euclidean distances of these multi-timepoint expression profiles, the algorithm automatically stratified metabolites and proteins with highly synchronous dynamic trajectories into distinct subclasses. For instance, Subclass 3 (Figure 3A), which exhibited a continuously decreasing trajectory, enabled us to precisely pinpoint the depletion trend of phosphatidylcholine (PC) that highly correlates with disease progression. This provided a crucial systems-biology context for resolving the timeline of postoperative injury and repair.

Methodological Details of the K-Means Implementation.

We would like to provide the specific parameters utilized to ensure full reproducibility:

Software: The analysis was executed using the K-Means function within the R stats

package (v4.1.2).

Distance Metric: Euclidean distance was employed to measure the similarity between multi-timepoint data points.

Selection of K: The optimal number of clusters (K = 3) was determined by combining domain knowledge with the statistical Elbow method to ensure detailed structural resolution while minimizing noise interference.

Convergence: The algorithm iteratively minimized the within-cluster sum of squares, and the centroids successfully converged after iterations in our specific dataset.

To eliminate any potential ambiguity for future readers, we have comprehensively revised the manuscript and have expanded the corresponding analytical steps in the Results and Methods sections.

Result：

Lipidomics (Line 311-317): The K-Means clustering algorithm was applied to the abundance matrix of significantly differential lipids, for investigating the dynamic temporal patterns of lipid profiles during the acute injury phase. Using Euclidean distance measurements, metabolites sharing highly congruent dynamic trajectories were automatically partitioned into 3 subclasses (Figure 3A): Subclass1 was dominated by rising PE (Supplementary Figure 3E); Subclass2 showed similar PC and PE changes (Supplementary Figure 3F); Subclass3 showed the most significant downregulation of PC, correlating closely with glycerophospholipid metabolism (Figure 3B-C)

Proteomics (Line 374-376): Similarly, K-Means clustering algorithm was applied to the temporal expression profiles of the differentially expressed proteins. Based on their temporal trajectories, these proteins were stratified into 6 Subclasses (Figure 5B, Supplementary Figure 6B-C). Subclass3, which mirrored the PC trend, was closely related to lipid metabolism (Figure 5C) and cytoplasmic localization (Figure 5D).

Method (Line 875-889): K-Means clustering analysis

To elucidate the dynamic evolutionary patterns of lipid and protein profiles during the acute injury phase following PHx, K-Means clustering of temporal expression profiles was performed using the R stats package (version 4.1.2). Expression matrices comprising significantly differentially expressed lipids and proteins across Control (C) and early post-PHx time points (D1, D2, and D3) were utilized as input features. The optimal number of clusters (K) was determined by integrating the Elbow method with biological interpretability. The algorithm partitioned metabolites and proteins into distinct subclasses based on the Euclidean distance of their multi-time-point expression trajectories, clustering features with highly similar dynamic patterns. Iterative calculations of cluster centroids were executed until convergence criteria were met. This temporal clustering approach enabled the identification of specific expression modules, facilitating the precise characterization of disease and providing a comprehensive systems biology context for post-operative injury.

10. All omics data and code for the reproduction of this study are missing, which is not acceptable.

Response: We sincerely thank the reviewer for their insightful comments and constructive suggestions, which have helped us to substantially improve the rigor, clarity, and overall quality of our work. We fully agree that the public availability of all raw data and custom analytical code is an absolute prerequisite for ensuring the reproducibility and transparency of our study. We sincerely apologize for this critical omission in our initial submission. To completely resolve this issue and strictly adhere to the journal's open science policies, we have now deposited all multi-omics datasets into specialized, publicly accessible international repositories. Furthermore, the complete, well-commented R scripts required to reproduce our bioinformatics and machine learning pipelines have been made publicly available.

Specifically, the data and code can now be accessed via the following platforms and accession numbers (Line 1018-1039): ScRNA-seq data have been deposited in the Sequence Read Archive (SRA) under accession number PRJNA1490015 (https://dataview.ncbi.nlm.nih.gov/object/PRJNA1490015?reviewer=k5jlep8rqs5t9lj7ba9ous5g5f). Proteomics data have been deposited to the ProteomeXchange Consortium via the iProX partner repository[8,9] with the dataset identifier PXD080626 (https://proteomecentral.proteomexchange.org/cgi/GetDataset?ID=PXD080626; IPX0018189000; https://www.iprox.cn/page/DSV021.html;?url=1783239456569lSKY; Password: crd9). The mRNA sequencing data generated in this study have been deposited in the

China National Center for Bioinformation database under accession code PRJCA068365 (OMIX018348; https://ngdc.cncb.ac.cn/omix/preview/eVvRK3WN). Lipidomics generated in this study have been deposited to MetaboLights repository[10] with the study identifier MTBLS14980 (https://www.ebi.ac.uk/metabolights/reviewerbd325d4c-c5a6-4f1f-94a4-e5915872835 7). All data supporting the findings of this study are included in the main text, the Supplementary Information, or the Source Data file. Source data are provided with this paper. All custom R scripts utilized for WGCNA, machine learning, multi-omics integration, and single-cell transcriptomic analyses are accessible via GitHub (https://github.com/tys980915/code.git), accompanied by a README guide to facilitate the reproduction of the analytical pipeline.

Accordingly, we have incorporated comprehensive "Data and Code Availability" statements at the end of the revised manuscript to provide explicit guidance for future readers. We hope these comprehensive updates fully satisfy your rigorous equirements for reproducibility.

Reviewer #2 (Remarks to the Author):

This study employs multi-omics integrative analysis and machine learning algorithms to propose a novel mechanism whereby the DR5-FXR-CHPT1 axis regulates hepatocyte pyroptosis following major partial hepatectomy. The study is highly innovative because previous studies never established the causal relationship between

glycerophospholipid metabolism and pyroptosis. However, this study lacks several critical evidences and thus the conclusion is not solidly supported. The manuscript will be more suitable for publication in Nature Communications if these evidences are supplemented. My specific suggestions are as follows:

1. Figure 1D demonstrates upregulation of pyroptosis-related proteins; however, could this result potentially arise from pyroptosis occurring in cell types other than hepatocytes? Please clarify which cell type(s) primarily undergo pyroptosis following major partial hepatectomy.

Response: We sincerely thank the reviewer for this rigorous and insightful question. We completely agree that because the liver is a highly heterogeneous organ comprising multiple cell types, whole-tissue western blot analysis cannot definitively distinguish the specific cellular origin of the pyroptotic signals. To precisely address this and identify the predominant cell population undergoing pyroptosis, we have incorporated two complementary layers of high-resolution evidence in the revised manuscript: We first deeply mined scRNA-seq datasets. Utilizing the UCell algorithm[11] to evaluate pathway enrichment, we found that the pyroptosis score was specifically elevated in the hepatocyte subpopulation across all evaluated postoperative time points (D1-D3) following massive PHx (Response Figure 5). To provide direct morphological validation of this computational prediction at the protein level, we have supplemented multiplex immunofluorescence double-staining in the revised manuscript. By co-staining the hepatocyte-specific nuclear marker (HNF4α) with the core pyroptosis executioner protein (GSDMD-N), we unequivocally

observed that the GSDMD-N signal predominantly co-localizes with HNF4α-positive cells, but to a lesser extent with endothelial (CD31) or myeloid (CD11b) cell markers. Together, these newly added lines of cross-validated evidence visually and definitively demonstrate that hepatocytes are indeed the core parenchymal cell population undergoing massive pyroptosis in the remnant liver following extensive partial hepatectomy.

We have comprehensively revised the manuscript and have expanded the corresponding content in the Results sections (Line 211-222): "To further identify the primary effector cells undergoing pyroptosis following extensive PHx, we conducted an in-depth analysis of Single-cell RNA sequencing (scRNA-seq) data. Evaluation using the UCell algorithm revealed a significant and highly specific elevation in pyroptosis pathway scores within hepatocyte subpopulations across all evaluated postoperative time points (D1-D3) (Figure 1F, Supplementary Figure 1H). Multiplex immunofluorescence (mIF) staining corroborated these findings, demonstrating that the core pyroptotic executioner GSDMD-N highly colocalized with the hepatocyte marker HNF4α, but to a lesser extent with endothelial (CD31) or myeloid (CD11b) cell markers (Supplementary Figure 1I). Collectively, these results strongly indicate that hepatocytes as the primary effector cells undergoing pyroptosis following extensive PHx."

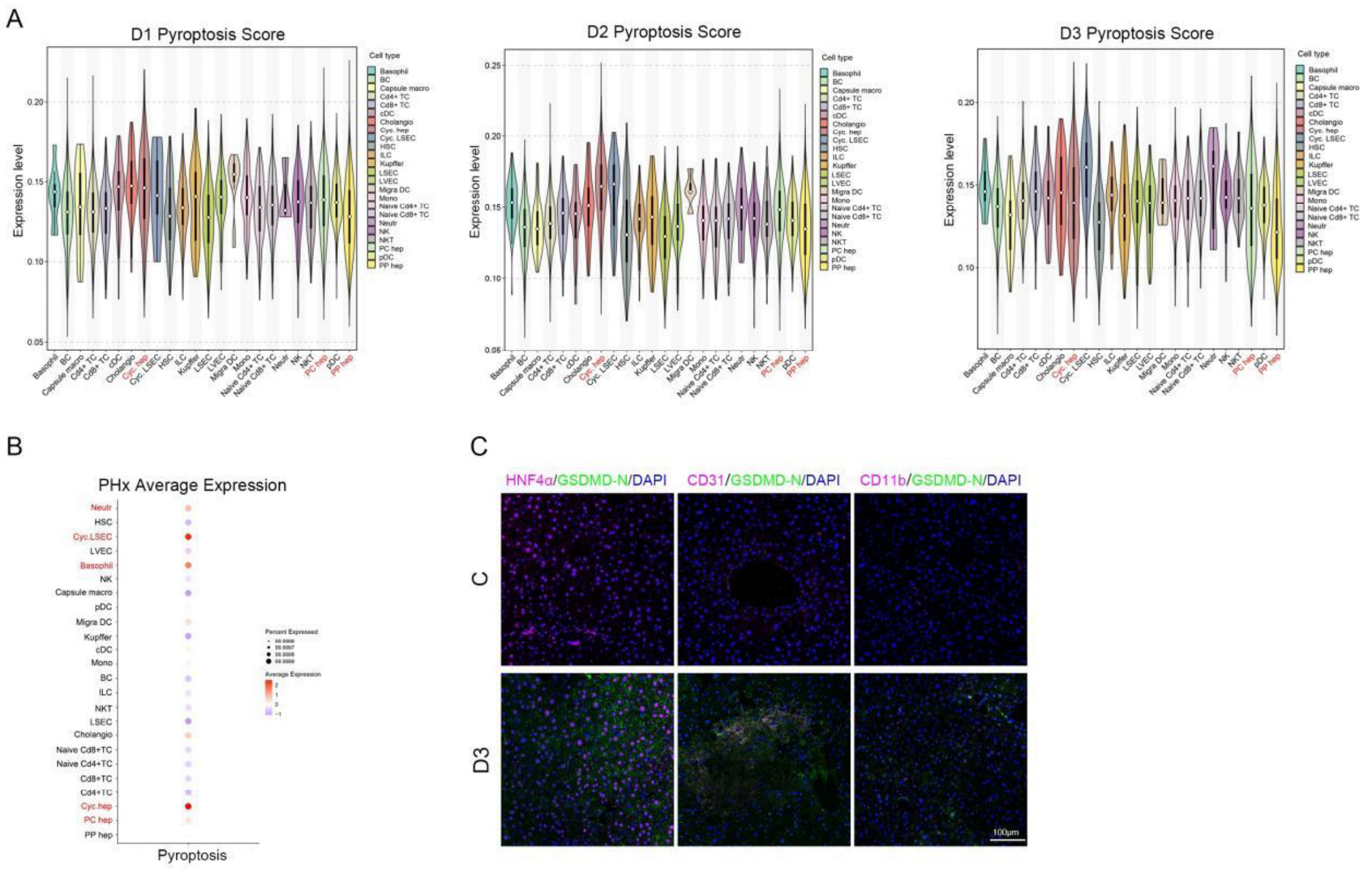

Response Figure 5. Single-cell RNA sequencing (scRNA-seq) analysis shows pyroptosis scores at D1-D3 (A) and average expression patterns (B) after 70% PHx across distinct hepatic cellular clusters by using the UCell method (CNP0002310). C: Representative immunofluorescence co-staining of the major cell types undergoing pyroptosis in liver tissues following 70% PHx (n=3 independent experiments).

2. Figure 1F presents transmission electron microscopy (TEM) observations of extensive membrane pore formation and organelle structural damage in hepatocytes. How do the authors demonstrate that the cells observed by electron microscopy are indeed hepatocytes?

Response: We highly appreciate this careful comment from the reviewer, which raises a highly professional morphological question[12,13].

To enable readers to more clearly discern these features, we have now incorporated distinct arrows and detailed annotations in the revised Source Data, explicitly pinpointing these signature structures (e.g., bile canaliculi and dense mitochondrial clusters).

Guided by these classic ultrastructural landmarks, we can definitively confirm that the cells exhibiting pyroptotic features, severe membrane poration and organelle collapse, are indeed hepatocytes. We hope these detailed morphological clarifications and the newly annotated images fully address your concern.

TEM (Supplementary Figure 1M)

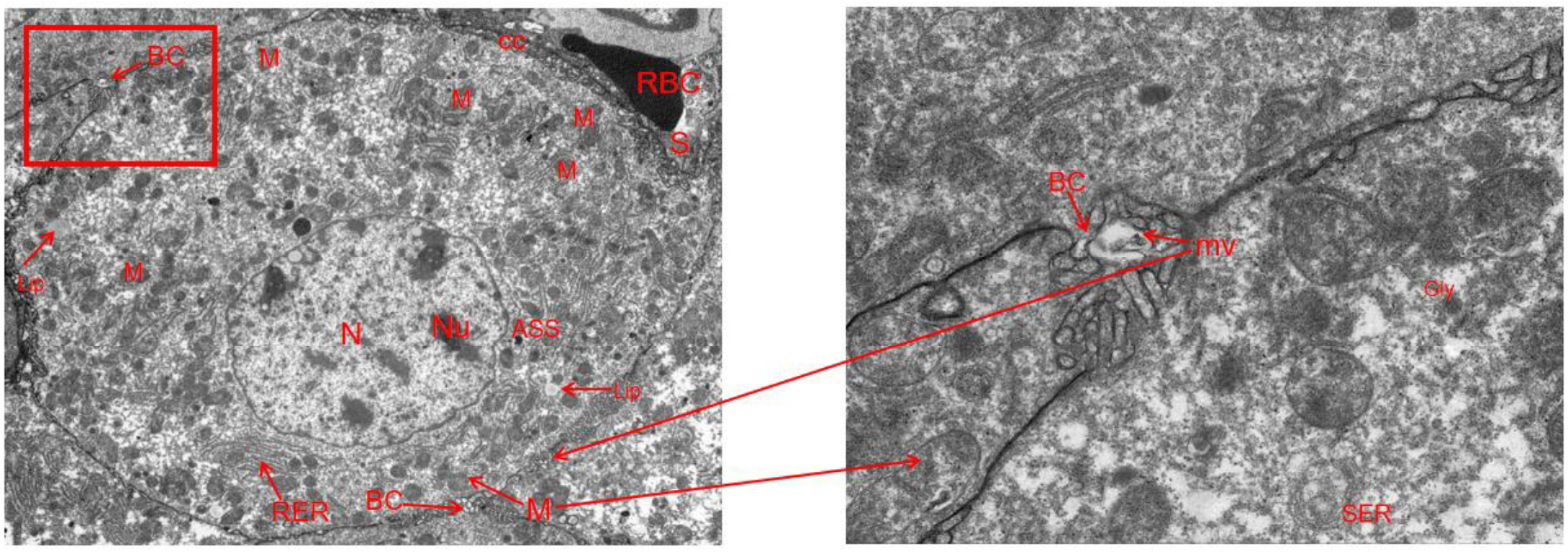

BC, bile canaliculus; cc, cell junction; Gly, glycogen granules; Lip, lipid droplet; M, mitochondria; N, nucleus; Nu,Nucleolus;RER, rough endoplasmic reticulum; S, hepatic sinusoid; SER, smooth endoplasmic reticulum; mv: Microvillus; ASS, Autolysosome; RBC, Red Blood Cell.

SEM

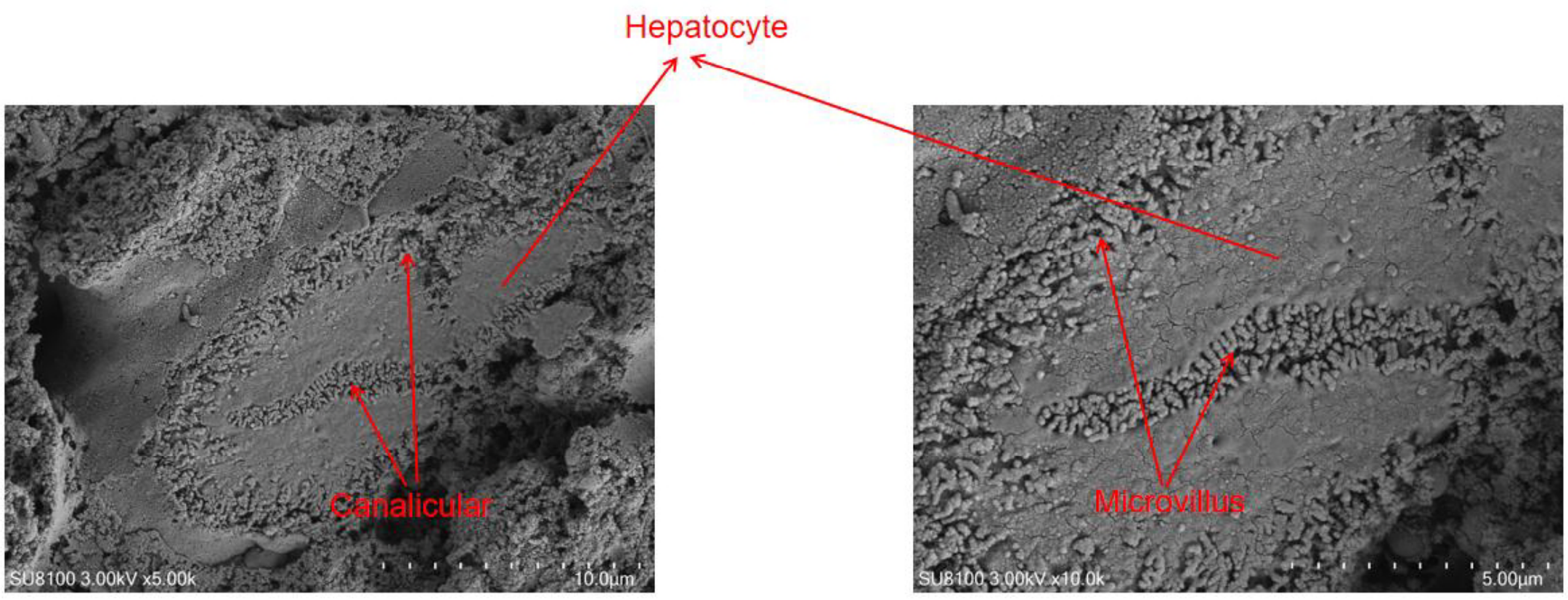

Response Figure 6. Specific ultrastructural characteristics of hepatocytes.

3. The authors conducted DR5 overexpression and knockdown experiments in both cellular and animal models; however, they should assess the efficiency of DR5 overexpression or knockdown.

Response: We completely agree with the reviewer's rigorous suggestion. Demonstrating the efficiency of these genetic manipulations is indeed a fundamental prerequisite for ensuring the robustness and validity of our experimental conclusions. While we had routinely verified these efficiencies during our study, we acknowledge that these crucial validation data were not sufficiently highlighted in our original manuscript. To address this, we have now comprehensively presented the Real-time quantitative PCR (RT-qPCR) and Western blot data in the revised manuscript, demonstrating the robust knockdown and overexpression efficiencies of DR5 across both cellular and animal models. All validation experiments were accompanied by explicit reporting of sample sizes (n) and demonstrated significant statistical differences.

In vitro efficiency validation: We have provided clear evidence for the potent silencing efficiency in our DR5-knockdown (DR5-KD) THLE-2 cell lines (Response Figure 7A-C). Concurrently, the robust upregulation of DR5 at both the mRNA and protein levels in our DR5-overexpressing (DR5-OE) THLE-2 model (Response Figure 7D-G).

In vivo efficiency validation: To confirm the efficacy of our in vivo genetic interventions, we systematically validated the precise hepatocyte-specific tropism and the highly efficient DR5 knockdown/overexpression achieved by our adeno-associated viral vector (AAV-DR5-KD/OE) in the 70% PHx mouse model (Response Figure 7H-O). The successful establishment of these highly efficient manipulation models laid a solid foundation for the downstream functional

phenotypes. We have updated the Results and Supplementary Information sections to explicitly reference these efficiency validation data, ensuring that the robustness of our genetic models is clearly conveyed to the readers.

(Line 358-361): We established two distinct DR5-knockdown (DR5-KD) THLE-2 cell lines (Supplementary Figure 5F) and observed that DR5-KD significantly reduced pyroptosis-related protein expression in vitro (Figure 4I, Supplementary Figure 5G).

(Line 392-394): In parallel, we established a DR5-overexpressing (DR5-OE) THLE-2 cell line (Supplementary Figure 6I-J), DR5-OE increased pyroptosis, which was mitigated by GW4064 (Figure 5M).

(Line 416-419): To evaluate this axis in vivo, mice were administered an AAV-DR5-KD vector featuring a TBG promoter for hepatocyte-specific targeting. We confirmed its precise liver tropism and potent silencing efficiency in the 70% PHx model (Figure 6I, Supplementary Figure 7F-J).

(Line 429-445): To further substantiate these findings, an AAV-DR5-OE vector with verified liver-specific tropism and high transduction efficiency was employed in the 70% PHx model (Supplementary Figure 7M-Q). AAV-DR5-OE treatment markedly decreased mouse survival, whereas FXR activation via GW4064 co-treatment mitigated this escalating mortality trend (Figure 6N). Concurrently,

AAV-DR5-OE-treated mice exhibited drastically elevated serum ALT and AST levels (Figure 6O, Supplementary Figure 7R) alongside a substantially increased area of hepatic damage (Figure 6P-Q). Activation of FXR with GW4064 markedly ameliorated these liver injuries, partially abrogating the detrimental impacts of DR5 overexpression. SEM and TEM imaging further confirmed that GW4064 effectively maintained organelle integrity, counteracting the AAV-DR5-OE-induced aggravation of plasma membrane poration, sinusoidal congestion, and mitochondrial swelling. Mechanistically, WB analysis revealed that AAV-DR5-OE suppressed CHPT1 expression and triggered the massive upregulation of pyroptosis markers (NLRP3, GSDMD-N, Cleaved Caspase-1, and IL-1β). Notably, GW4064 intervention reversed these molecular alterations, restoring CHPT1 levels while effectively dampening the pyroptotic protein signature (Figure 6R, Supplementary Figure 7S)."

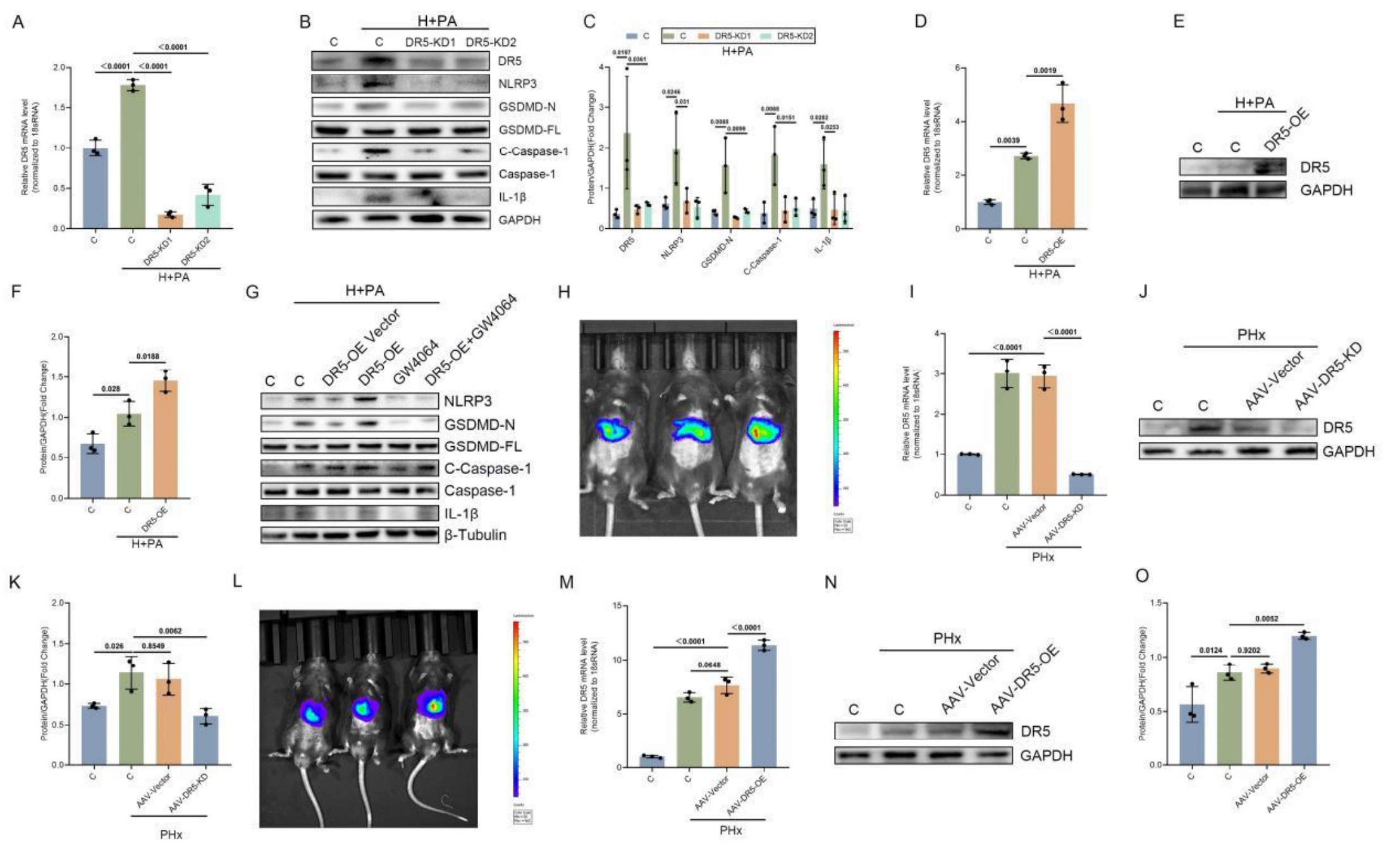

Response Figure 7. A: RT-qPCR validation of DR5 expression levels following DR5 knockdown (n=3 independent experiments). B: Representative western blot analysis of pyroptosis-related proteins in the cell model following DR5 knockdown (DR5-KD)

(n=3 independent experiments). C: Quantitative statistical analysis of protein expression levels corresponding to Response Figure 7B (n=3 independent experiments). D: RT-qPCR validation of DR5 mRNA expression levels following DR5 overexpression (n=3 independent experiments). E: Representative western blot images of DR5 protein expression levels. F: corresponding quantification following DR5 overexpression in the cell model (n=3 independent experiments). G: Representative western blot images of pyroptosis-related proteins in the cell model following DR5 overexpressing (DR5-OE) combined with the FXR agonist GW4064 (n=3 independent experiments). H: In vivo imaging showing specific targeted infection and expression in the liver region following AAV-DR5-KD virus injection (n=3 per group). I: RT-qPCR validation of DR5 mRNA expression levels following AAV-DR5-KD administration (n=3 independent experiments). J: Representative western blot images of DR5 protein expression levels following AAV-DR5-KD administration (n=3 independent experiments). K: Quantitative statistical analysis of protein expression levels corresponding to Response Figure 7J (n=3 independent experiments). L: In vivo imaging showing specific targeted infection and expression in the liver region following AAV-DR5-OE virus injection (n=3 per group). M: RT-qPCR validation of DR5 mRNA expression levels following AAV-DR5-OE administration (n=3 independent experiments). N: Representative western blot images of DR5 protein expression levels following AAV-DR5-OE administration (n=3 independent experiments). O: Quantitative statistical analysis of protein expression levels corresponding to Response Figure 7N (n=3 independent experiments).

4. For Western blot of GSDMD-N and C-caspase-1, the full length protein should also be detected.

Response: We highly appreciate the reviewer's constructive feedback and rigorous scientific standard. We fully agree that in studies of programmed cell death, detecting the full-length precursor proteins alongside their cleaved forms is essential to accurately demonstrate pathway activation. Accordingly, we have performed the supplementary western blot analyses and incorporated the bands for full-length GSDMD (GSDMD-FL) and Caspase-1across all relevant western blot figures in the revised manuscript. These precursor proteins are now displayed in parallel with their active cleaved counterparts (GSDMD-N and Cleaved Caspase-1, respectively). This

comprehensive presentation provides a more rigorous and complete evaluation of the pyroptotic activation cascade following massive PHx, clearly demonstrating the robust cleavage events during disease progression.

5. For Oil Red O staining, quantitation should also be shown.

Response: We highly appreciate the reviewer's constructive suggestion. We fully agree that quantitation is essential for a more objective evaluation of the lipid accumulation. Accordingly, we have performed rigorous quantitative analyses of the Oil Red O staining images across all experimental groups and time points. The corresponding statistical graphs have now been incorporated into the revised manuscript (Response Figure 8). We believe these newly added quantitative data provide more robust and objective statistical evidence regarding the severe lipid metabolic disorder in the remnant liver following extensive PHx, thereby further consolidating our mechanistic conclusions.

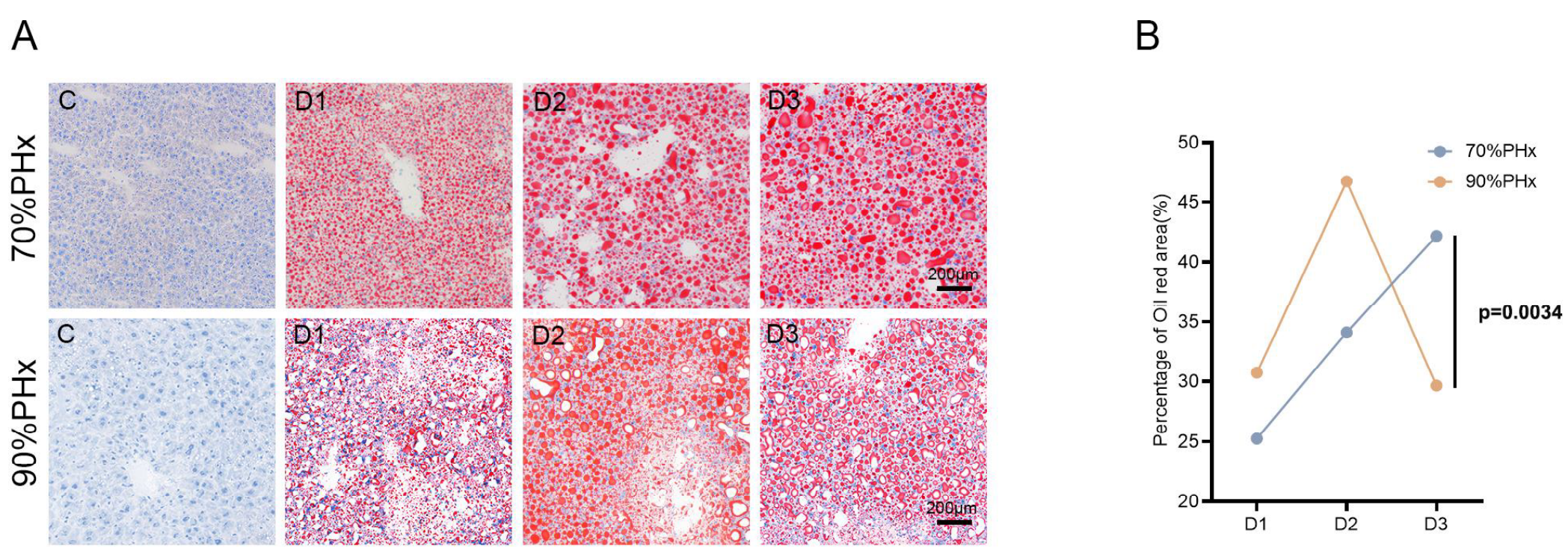

Response Figure 8. A: Representative Oil Red O staining images of liver tissue following 70% PHx and 90% PHx (Scale bar=200 μm, n=5 independent experiments). B: Quantitative statistical analysis of Oil Red O staining corresponding to Response Figure8A.

6. According to a previous study (PMID: 34890842) and scRNA-seq data from human protein atlas website (https://www.proteinatlas.org/), the expression level of NLRP3 and caspase-1 in hepatocytes are extremely low in untreated state. The authors should explain why pyroptosis could occur in hepatocytes. Are there expressions induced by PHx? What is the possible mechanism?

Response: We sincerely thank the reviewer for citing this important previous study and for raising this core mechanistic question regarding the baseline expression context. The reviewer's observation is entirely correct, under physiological homeostasis (the untreated state), the baseline expression of NLRP3 inflammasome components in hepatocytes is indeed maintained at a low level[14]. However, we found that massive PHx strongly induces their expression and triggers massive hepatocyte pyroptosis[15-17]. This phenomenon perfectly aligns with the classical "Two-Signal Activation" model of the inflammasome. PHx constitutes an extreme metabolic and inflammatory stress. The complete molecular logic driven by PHx in our model can be delineated as follows:

Signal 1 (Priming): The damage-associated molecular patterns (DAMPs) released from surgical trauma (within 48 hours), the sudden alterations in hemodynamic shear stress, and the acute, massive influx of lipids serve as a robust "priming signal". These stimuli strongly activate the NF-κB transcriptional cascade within hepatocytes, which in turn profoundly induces the transcription and de novo synthesis of *NLRP3* and *Pro-IL-1β* (Response Figure 9A-C).

Signal 2 (Activation): Building upon this initial priming phase, our study reveals

that the massive PHx-mediated dysregulation of the DR5-FXR-CHPT1 axis leads to a severe depletion of phosphatidylcholine (PC) in the remnant liver. Because PC is a crucial glycerophospholipid for maintaining endoplasmic reticulum (ER) membrane homeostasis, its drastic deficiency provokes severe ER stress (ERS). This metabolic remodeling-induced membrane instability acts as the lethal "Signal 2," triggering the oligomerization and assembly of the newly synthesized NLRP3 proteins. This assembled complex subsequently cleaves Pro-Caspase-1 into its active form, ultimately executing pyroptosis.

By incorporating this dual mechanism, the severe PC depletion orchestrated by the DR5 axis acts as the decisive trigger for inflammasome assembly, perfectly bridging the gap between glycerophospholipid metabolic disorder and inflammatory cell death. To ensure this theoretical framework is clear to all readers, we have explicitly incorporated this complete molecular logic of "stress-induced priming + lipid disorder-triggered activation" into the **Results** and **Discussion** section of the revised manuscript. We believe this addition makes our hypothesis regarding lipid-regulated pyroptosis highly self-consistent and persuasive.

Results section (Line 302-308): During the early phase of hepatectomy (within 48 hours), surgical trauma prompts the release of damage-associated molecular patterns (DAMPs), such as HMGB1, to activate the NF-κB signaling pathway, ultimately triggering NLRP3-mediated pyroptosis (Supplementary Figure 3A-C). However, the mechanisms underlying the subsequent exacerbation of pyroptosis warrant deeper

exploration. A crucial clue emerged from our preceding observation that lipid remodeling profoundly influences the occurrence of pyroptosis following 70% PHx.

Discussion section (Line 494-503): Under physiological homeostasis, the expression of NLRP3 inflammasome components in hepatocytes is indeed maintained at a low basal level. However, our findings reveal that extensive PHx robustly upregulates their expression to trigger hepatocyte pyroptosis. We postulate that damage-associated molecular patterns (DAMPs) released by surgical trauma, sudden shifts in hemodynamic shear stress, and the acute, massive influx of lipids post-surgery collectively serve as 'priming signals'. These signals activate the intracellular NF-κB cascade, thereby transcriptionally inducing Nlrp3 and Pro-IL-1β. Nevertheless, the mechanisms driving the subsequent and more critical exacerbated phase of pyroptosis warrant further in-depth exploration.

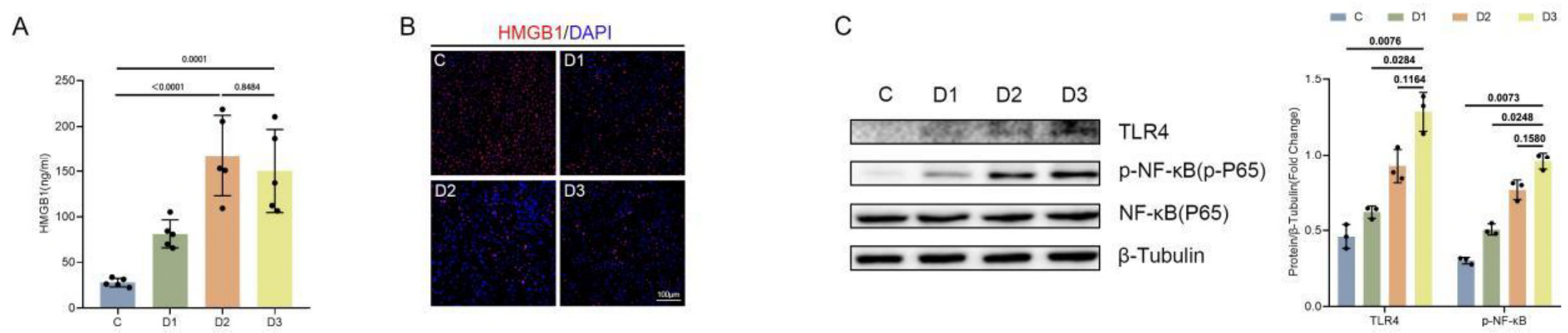

Response Figure 9. A: Serum HMGB1 concentrations at the indicated time points (C, D1, D2, and D3) following 70% PHx, as determined by ELISA (n=5 per group). B: Representative immunofluorescence images of HMGB1 (red) and nuclei (DAPI, blue) in liver tissues (Scale bar=100 μm, n=3 independent experiments). C: Representative western blot images (left panel) and corresponding quantitative analysis (right panel) of TLR4 and p-NF-κB (n=3 independent experiments).

Reviewer #3 (Remarks to the Author):

The authors used Multi-omics approaches combined with machine learning method

reveals the DR5-FXR-CHPT1 axis governs hepatocyte pyroptosis via glycerophospholipid remodeling after major partial hepatectomy. The data was comprehensive and the writing was largely well. I have the following minor concerns and comments mainly focus on proteomics and lipidomics analyses:

1. The number of replicates should be stated clearly for proteomics and lipidomics analysis

Response: We fully agree with the reviewer and sincerely apologize for this omission in our original manuscript. To ensure full transparency and statistical rigor, we have now explicitly stated the exact number of biological replicates in corresponding Figure Legends in the revised manuscript. Specifically, a robust sample size of n = 6 independent biological replicates per group was utilized for both the comprehensive proteomics and lipidomics analyses. We hope this clarification fully resolves your concern regarding the experimental design and statistical power.

2. How much peptide was injected to mass spec for proteomics analysis? Will the proteomics data uploaded to Pride or other places?

Response: We sincerely thank the reviewer for this rigorous and insightful question. For LC-MS/MS proteomic analysis, the accurate number of peptides analyzed per sample was 200 ng. To strictly comply with open science policies and ensure full data accessibility, the complete proteomics datasets have been successfully deposited to the ProteomeXchange Consortium via the iProX partner repository[8,9]. The dataset identifier is PXD080626 (IPX0018189000). For the reviewer's convenience during

the evaluation process, the data can be directly accessed via the following link: URL: https://www.iprox.cn/page/DSV021.html?url=1783239456569lSKY; Password: crd9. We have also incorporated this accession number into the "Data Availability" section of the revised manuscript. We hope these comprehensive clarifications fully resolve your concerns regarding our experimental design, technical details, and data transparency.

3. Lipidomis analysis: Since MRM method was used, what are the target lipids analyzed? Was the lipid quantification based on internal lipid standards or it is a relative quantification?

Response: We sincerely thank the reviewer for the excellent and highly methodological question. Lipid quantification was performed using the Multiple Reaction Monitoring (MRM) mode on a triple quadrupole mass spectrometer[18,19]. In MRM mode, the first quadrupole specifically selects the precursor (parent) ions of the target analytes, filtering out ions of other molecular weights to minimize initial background interference. These precursor ions are then fragmented in the collision cell via collision-induced dissociation, generating numerous product (fragment) ions. The third quadrupole then filters and isolates a specific, characteristic product ion. This process eliminates non-target ion interference, resulting in highly precise quantification and excellent reproducibility. After acquiring the lipid mass spectrometry data across different samples, the chromatographic peaks of all identified substances are integrated, and quantitative analysis is performed using the

internal standard method. Target Lipids: The targeted MRM approach was specifically designed to analyze major lipid classes crucial for hepatic lipid remodeling.

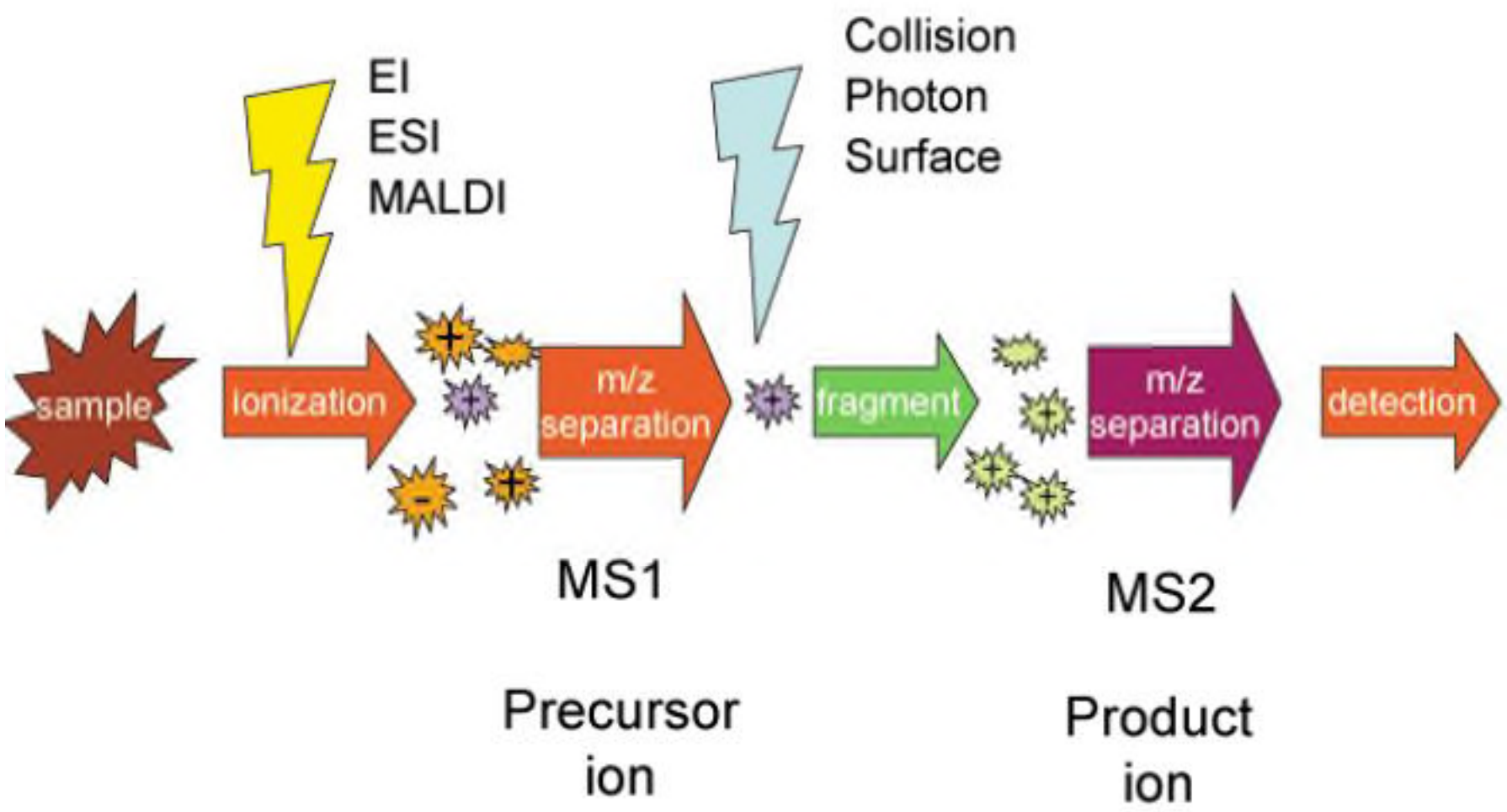

The primary targets include glycerophospholipids (e.g., PC, PE, PS, PI), sphingolipids (e.g., SM, Cer), and neutral lipids (e.g., TG, DAG, FFA).

Quantification Method: Qualitative MS analysis of the sample lipids was conducted based on an in-house lipid database. The chromatographic peaks detected for each substance across different samples were then aligned and corrected to ensure quantitative accuracy. The lipid quantification in this study is a semi-absolute quantification based on internal lipid standards. The integrated peak area ratios detected in all samples were substituted into a linear regression equation and further processed to obtain the final content of each substance in the experimental samples.The formula for calculating lipid content in the samples is as follows:

$X = 0.001 * R * c * F * V/m$

X: Lipid content in the sample (nmol/g)

R: Area ratio of the analyte's peak to the internal standard's peak

F: Internal standard correction factor for different lipid classes

c: Concentration of the internal standard (μmol/L)

V: Volume of the sample extraction buffer (μL)

m: Mass of the weighed sample (g)

4. How exactly Multi-omics integration (Metabolomics+Proteomics+Transcriptomics) was done?

Response: We sincerely thank the reviewer for this crucial and constructive comment. The multi-omics integration was performed by combining the KEGG functional enrichment results of differentially expressed genes (DEGs)[20], differentially abundant metabolites (DAMs), and differentially expressed proteins (DEPs) to conduct a multi-omics co-enrichment analysis. KEGG pathway enrichment analysis was performed independently for the transcriptomic, metabolomic, and proteomic datasets. we screened for and identified the pathways that were commonly enriched across all three omics levels. Finally, based on the enrichment intensity metrics of each omics dataset within these shared pathways — specifically the p-value, enrichment factor, and feature count — we generated a co-enrichment bubble plot to visualize the integrated results.

Other minor points:

• Line 122: add a comma at the end

Response: A comma has been added at the end of the line.

• Line 939-948 need to be re-edited

Response: This paragraph has been revised and overlaps with the **Multiplex Immunofluorescence (mIF) Staining** ， methods section (Line 972-989):

Multiplex immunofluorescence (mIF) staining

Multiplex immunofluorescence (mIF) staining was performed on formalin-fixed, paraffin-embedded liver sections using a 4-color Multiplex Fluorescent Immunohistochemistry Kit (Servicebio, Wuhan, China). Following deparaffinization and microwave-based antigen retrieval in citrate or EDTA buffer, endogenous peroxidase was blocked, and sections were incubated with primary antibodies overnight at 4°C. A sequential staining protocol was employed based on Tyramide Signal Amplification (TSA) technology, where each cycle consisted of HRP-conjugated secondary antibody incubation, TSA-fluorophore reaction, and heat-induced antibody stripping. Target proteins were grouped into distinct panels on serial sections, utilizing antibodies against CD206 (#24595, CST), iNOS (ab3523, Abcam), F4/80 (ab300421, Abcam), CD3 (ab135327, Abcam), CD19 (ab245235, Abcam), NCR-1 (ab233558, Abcam), DR5 (ab8416, Abcam), HMGB1 (ab79823, Abcam), HNF4α (ab201460, Abcam), CD31 (ab281583, Abcam), CD11b (ab133357, Abcam), GSDMD-N (A24059, ABclonal) and NR1H4 (#72105, CST, Danvers, MA, USA), as well as CHPT1 (A16137, ABclonal). Finally, nuclei were counterstained with DAPI, and images were captured using a laser scanning confocal microscope (Zeiss LSM 880).

• Line 982: remove “with were measured”

Response: The redundant phrase "with were measured" has been removed and replaced with a grammatically correct expression.

• Line 987: change “Cell model” to “cell model”

Response: The “Cell model” has Changed to “cell model”.

• Keep consistent for “min” or “minutes” for the whole manuscript

Response: The entire text uses the abbreviation "min" uniformly, and full-text search replacement is complete.

• Line 1049: change “detection” to “measurement”

Response: The “detection” has Changed to “measurement”.

• Line 1052: add space before “Briefly”; change “tissues” to “tissue”

Response: A space has been added before “Briefly”; “tissues” has been changed to “tissue”.

• Line 1124: change to “Proteomics analysis by LC-MS/MS”

Response: This sentence has been changed to“Proteomics analysis by LC-MS/MS”。

• Line 1125: the sentence need to be re-edited

Response: We have revised this sentence to improve clarity (Line 825-827):

Tissue samples were ground into powder in liquid nitrogen. The powder was lysed in lysis buffer containing 8 M urea, 1 mM PMSF, and 2 mM EDTA, followed by sonication on ice for 5 min.

• Line 1138: remove "LC-MS/MS Analysis (Orbitrap Astral)"

Response: We have deleted the subheading "LC-MS/MS Analysis (Orbitrap Astral)".

• Line 1144: change "Mass spectrometry was performed" to "After LC separation, the peptides were analyzed"

Response: "Mass spectrometry was performed" has been changed to "After LC separation, the peptides were analyzed by...".

• Line 1152: delete "Data Processing"

Response: We have deleted the independent subheading "Data Processing".

• Line 1158: change "Lipidomics Analysis" to "Lipidomics analysis by LC-MS/MS"

Response: We have changed "Lipidomics Analysis" to "Lipidomics analysis by LC-MS/MS".

• Line 1159: remove "Lipid Extraction"

Response: We have removed the independent subheading "Lipid Extraction".

• Line 1165: remove “UPLC-MS/MS conditions”

Response: We have removed the independent subheading “UPLC-MS/MS conditions”.

• Line 1221: remove “Multiplex Immunofluorescence Staining”

Response: We have removed the independent subheading “Multiplex Immunofluorescence Staining”.

• Line 1237: change “mm3” to “mm3”

Response: We have changed “mm3” to “mm^3”.

• Line 1245: change “Electron Microscopy” to “electron microscopy”

Response: We have changed “Electron Microscopy” to “electron microscopy”.

• Line 1246: change “mm^2” to “mm3”

Response: We have changed “mm^2” to “mm^3”

• Line 1247: add a space before “Samples”

Response: We have added a space before “Samples”.

• Line 1248: it is “PB” or “PBS”?

Response: it is “PBS”.

• Line 1254: add “was used” after “ANOVA”

Response: We have added “was used” after “ANOVA”.

• Line 1256: add a space before “Statistical”

Response: We have added a space before “Statistical”.

• Line 1258: change “performedusing” to “performed by”

Response: We have changed “performedusing” to “performed by”.

Line 1254: add “was used” after “ANOVA”, Line 1256: add a space before “Statistical”, Line 1258: change “performedusing” to “performed by”

Response: We have rewritten the entire paragraph (Line 1007-1017): All statistical analyses were performed using GraphPad Prism (version 8.0.1; GraphPad Software, La Jolla, CA, USA). Data normality was assessed using the Shapiro-Wilk test. For data that passed the normality test, comparisons between two independent groups were performed using a two-tailed unpaired Student’s t-test, while comparisons between paired groups were analyzed using a two-tailed paired Student’s t-test. Comparisons among three or more groups were performed using one-way (ANOVA), followed by Dunnett’s or Tukey’s multiple-comparisons test. For non-normally distributed data, comparisons between two groups were performed using the

nonparametric Mann-Whitney U test. Statistical methods and p values are detailed in the respective figure legends and figures. Source data are provided in the Source Data file.

• Fig 2L: is the unite for liver FFA "nmol"?

Response: We have verified the quantitative unit for hepatic free fatty acids (FFA) and corrected it to the correct value of "nmol/mg tissue," with this clarification included in the figure.

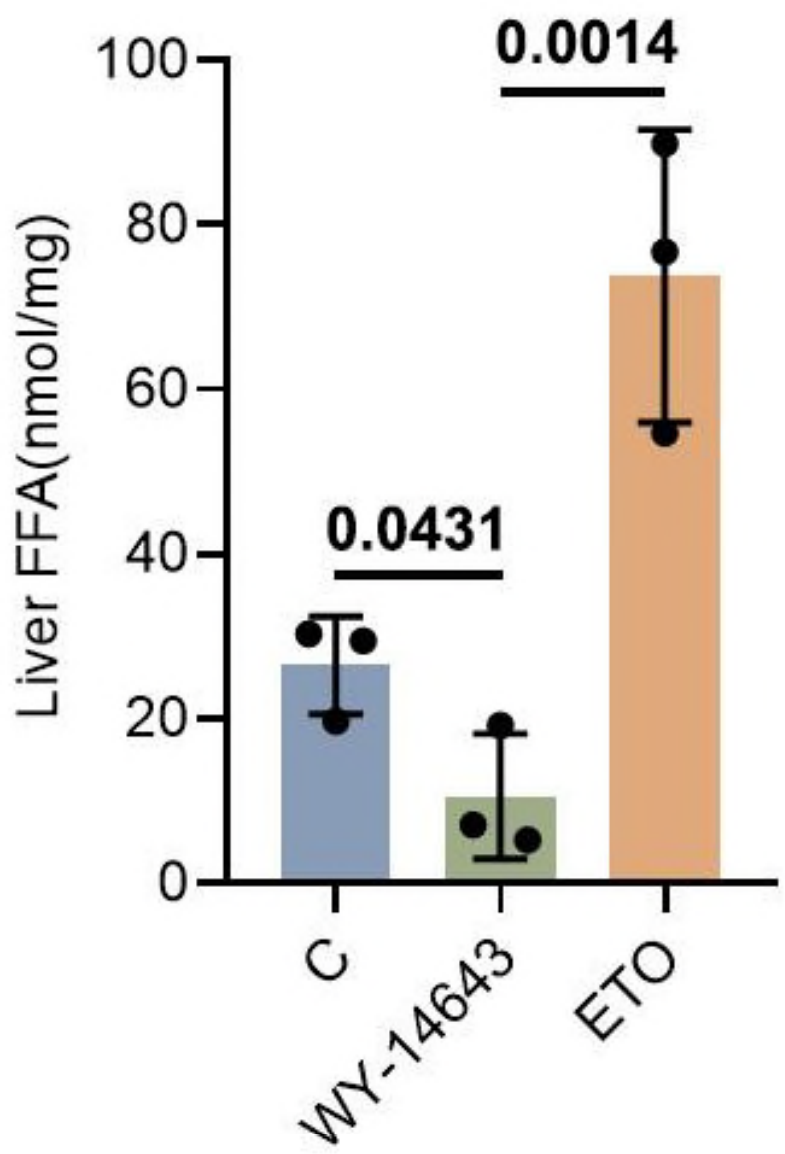

Response Figure 10. Statistical analysis of FFAs content in liver tissue at D3 post-70% PHx (n=3 per group. Data are presented as mean ± SD. Statistical significance was determined using ordinary one-way ANOVA followed by Tukey's multiple comparisons test).

• Fig 4A: add x-axis

Response: We have added x-axis.

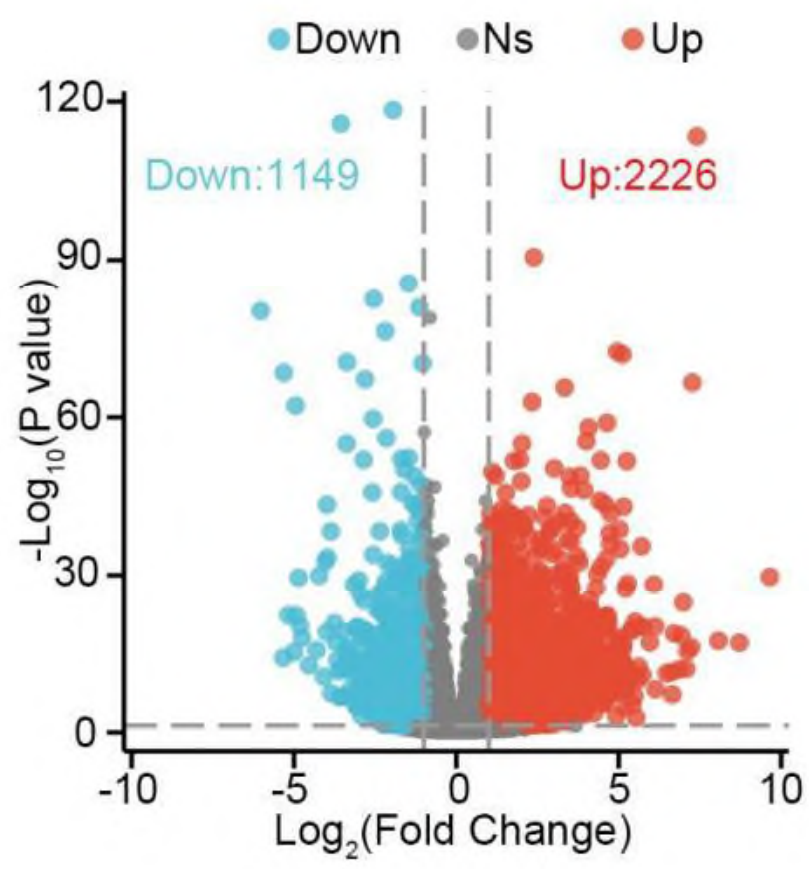

Response Figure 11. Volcano plot of DEGs in the first 3 days (n=36 samples from 18 mice).

Reference

1 Liu, Z. *et al.* Machine learning-enhanced discovery of a basement membrane-related gene signature in glioblastoma via single-cell and Spatial transcriptomics. *J Transl Med* **23**, 1325, doi:10.1186/s12967-025-06918-0 (2025).

2 Wu, D. *et al.* Integrative single-cell and spatial transcriptomics analysis reveals a baicalein-responsive 10-gene signature for non-small cell lung cancer. *Transl Oncol* **70**, 102853, doi:10.1016/j.tranon.2026.102853 (2026).

3 Xu, J. *et al.* A spatiotemporal atlas of mouse liver homeostasis and regeneration. *Nat Genet* **56**, 953-969, doi:10.1038/s41588-024-01709-7 (2024).

4 Xie, Z. *et al.* The enedioic acid analog 326E alleviates metabolic dysfunction-associated steatohepatitis via dual targeting at ACLY and PPARalpha. *Cell Metab* **37**, 2149-2166 e2149, doi:10.1016/j.cmet.2025.09.011 (2025).

5 Ling, J., Chaba, T., Zhu, L. F., Jacobs, R. L. & Vance, D. E. Hepatic ratio of phosphatidylcholine to phosphatidylethanolamine predicts survival after partial hepatectomy in mice. *Hepatology* **55**, 1094-1102, doi:10.1002/hep.24782 (2012).

6 Kiselev, V. Y. *et al.* SC3: consensus clustering of single-cell RNA-seq data. *Nat Methods* **14**, 483-486, doi:10.1038/nmeth.4236 (2017).

7 Semrau, S. *et al.* Dynamics of lineage commitment revealed by single-cell transcriptomics of differentiating embryonic stem cells. *Nat Commun* **8**, 1096, doi:10.1038/s41467-017-01076-4 (2017).

8 Ma, J. *et al.* iProX: an integrated proteome resource. *Nucleic Acids Research* **47**, D1211-D1217, doi:10.1093/nar/gky869 (2019).

9 Chen, T. *et al.* iProX in 2021: connecting proteomics data sharing with big data. *Nucleic Acids Res* **50**, D1522-D1527, doi:10.1093/nar/gkab1081 (2022).

10 Yurekten, O. *et al.* MetaboLights: open data repository for metabolomics. *Nucleic Acids Res* **52**, D640-D646, doi:10.1093/nar/gkad1045 (2024).

11 Lian, Q. *et al.* Serglycin's role in primary liver cancer: insights into tumor microenvironment and macrophage interaction. *Front Immunol* **16**, 1668627, doi:10.3389/fimmu.2025.1668627 (2025).

12 Parlakgul, G. *et al.* Regulation of liver subcellular architecture controls metabolic homeostasis. *Nature* **603**, 736-742, doi:10.1038/s41586-022-04488-5 (2022).

13 Chun Chung, G. H., Gissen, P., Stefan, C. J. & Burden, J. J. Three-dimensional Characterization of Interorganelle Contact Sites in Hepatocytes using Serial Section Electron Microscopy. *J Vis Exp*, doi:10.3791/63496 (2022).

14 Sun, P. *et al.* Hepatocytes Are Resistant to Cell Death From Canonical and Non-Canonical Inflammasome-Activated Pyroptosis. *Cellular and Molecular Gastroenterology and Hepatology* **13**, 739-757, doi:10.1016/j.jcmgh.2021.11.009 (2022).

15 Bauernfeind, F. G. *et al.* Cutting Edge: NF-κB Activating Pattern Recognition and Cytokine Receptors License NLRP3 Inflammasome Activation by Regulating NLRP3 Expression. *The Journal of Immunology* **183**, 787-791, doi:10.4049/jimmunol.0901363 (2009).

16 Swanson, K. V., Deng, M. & Ting, J. P. Y. The NLRP3 inflammasome: molecular activation and regulation to therapeutics. *Nature Reviews Immunology* **19**, 477-489, doi:10.1038/s41577-019-0165-0 (2019).

17 Lv, M., Zeng, H., He, Y., Zhang, J. & Tan, G. Dexmedetomidine promotes liver regeneration in mice after 70% partial hepatectomy by suppressing NLRP3 inflammasome not TLR4/NFκB. *International Immunopharmacology* **54**, 46-51, doi:10.1016/j.intimp.2017.10.030 (2018).

18 Narvaez-Rivas, M. & Zhang, Q. Comprehensive untargeted lipidomic analysis using core-shell C30 particle column and high field orbitrap mass spectrometer. *J Chromatogr A* **1440**, 123-134, doi:10.1016/j.chroma.2016.02.054 (2016).

19 Xuan, Q. *et al.* Development of a High Coverage Pseudotargeted Lipidomics Method Based on Ultra-High Performance Liquid Chromatography-Mass Spectrometry. *Anal Chem* **90**, 7608-7616, doi:10.1021/acs.analchem.8b01331 (2018).

20 Kanehisa, M. & Goto, S. KEGG: kyoto encyclopedia of genes and genomes. *Nucleic Acids Res* **28**, 27-30, doi:10.1093/nar/28.1.27 (2000).

Response Letter (Manuscript # NCOMMS-26-018614B)

Dear Editor and Reviewers,

We thank you very much for the thorough and thoughtful evaluation of our manuscript “Multi-omics reveals the DR5-FXR-CHPT1 axis governs hepatocyte pyroptosis via glycerophospholipid remodeling after major partial hepatectomy” (Manuscript # NCOMMS-26-018614B).

We greatly appreciate the reviewers’ insightful comments and constructive suggestions, which have helped us to substantially improve the rigor, clarity, and overall quality of the work.

We have carefully revised the manuscript in accordance with the suggestions provided. We are pleased to submit a revised version for your consideration which we believe now meets the expectations of the reviewers and journal. Detailed, point-to-point responses are provided in the sections that follow.

A brief summary of the main findings of the paper : After major partial hepatectomy, the DR5 protein disrupts lipid metabolism via the FXR-CHPT1 axis, triggering fatal cellular stress and pyroptosis. Targeting this pathway offers a therapeutic strategy to prevent liver failure.

Thank you again for your time and insightful suggestions. Please do not hesitate to contact me if any additional information is required

Yours Sincerely,

Hua Li MD. & Ph.D

Department of Hepatic Surgery, Liver Transplantation Center,

the Third Affiliated Hospital of Sun Yat-Sen University, Guangzhou 510630,

China

E-mail: lihua3@mail.sysu.edu.cn

Response to Reviewers' Comments

BLUE TEXT: Reviewer comments

BLACK TEXT: Author responses

RED TEXT: Changes made to text in manuscript

Reviewer #1 (Remarks to the Author):

I think the authors have addressed most of my concerns. My only remaining concern is the machine learning part. They used only 36 samples to train models with 26 input genes – that is too few samples for reliable machine learning. The results from this analysis are not very robust and add little beyond simpler statistical tests. The authors could have achieved the same conclusion (DR5 is a key gene) through differential expression and correlation analyses alone. The addition of 15 machine learning algorithms gives a superficial impression of sophistication but does not fundamentally advance the mechanistic understanding. The main biological conclusions do not depend on the ML, so I am not asking them to remove it. However, I think they should honestly acknowledge this limitation in the Discussion section.

Response:We sincerely thank the reviewer for this insightful and objective evaluation. We completely agree with your assessment. A sample size of 36 used to train models with 26 input features is indeed relatively small, which inherently limits the robustness, stability, and generalizability of the machine learning algorithms. Your observation is accurate. Our core biological conclusion identifies DR5 as a pivotal driver in this process. Traditional differential expression, scRNA-seq, and correlation analyses strongly support this finding. The machine learning screening was intended

as an adjunctive prioritization tool rather than the foundational evidence for our mechanistic claims.

Following your constructive suggestion, we have explicitly acknowledged this limitation in the revised Discussion section (Lines 540-549), ensuring readers interpret these specific screening results with appropriate caution.

(Lines 540-549):While our integrative analysis successfully prioritized DR5, we must acknowledge the limitations of the machine learning approach utilized in this study. The algorithms were trained on a relatively small cohort of 36 samples with 26 input genes, which inherently restricts the robustness and broad generalizability of the predictive models. Consequently, the machine learning results should be interpreted as an adjunctive screening tool rather than standalone definitive evidence. Nevertheless, our core mechanistic conclusions regarding the DR5-FXR-CHPT1 axis remain highly robust, as they are independently and strongly substantiated by comprehensive multi-omics differential expression, scRNA-seq, and extensive in vivo and in vitro experimental validations.

Reviewer #1 (Remarks on code availability):

The code include a lot of Chinese documentations, and is a bit messy to follow. It looks like a credible code history from the author, but I don't think I can reproduce everything from it.

Response: We sincerely apologize for the inconvenience caused by the messy code structure and the language barrier in the documentation. We deeply appreciate your

meticulous review. To ensure full transparency and facilitate seamless reproducibility for international readers, we have thoroughly overhauled the shared code repository. Specifically, we have implemented the following improvements:All Chinese documentation, inline comments, and variable descriptions have been meticulously revised.We have rewritten the README.md file to include detailed, step-by-step instructions, environment dependencies (R package versions), and exact execution orders to effortlessly reproduce the analytical pipeline.The updated and cleaned repository has been synced to both GitHub (https://github.com/tys980915/code.git) and the Zenodo archive (https://doi.org/10.5281/zenodo.21936844). We hope these revisions adequately address your concerns and significantly improve the code's accessibility.

Reviewer #2 (Remarks to the Author):

The authors have made substantial and careful revisions that fully address my previous concerns. The manuscript now presents a compelling, well-supported, and innovative mechanism linking glycerophospholipid metabolism to hepatocyte pyroptosis via the DR5-FXR-CHPT1 axis following major partial hepatectomy. The evidence is now solid, and the conclusions are appropriately supported. I have no further concerns and recommend acceptance of this revised manuscript for publication in Nature Communications.

Response: We would like to thank the reviewer for valuable suggestions and comments during the previous revision of the manuscript.
